# Supplementary material for: Prevalence and risk factors of lower extremity disease in high risk groups in Malawi: a stratified cross-sectional study
Source: BMJ Open. 2022 Aug 10;12(8):e055501. doi: 10.1136/bmjopen-2021-055501 (PMC9379496; doi:10.1136/bmjopen-2021-055501)
Supplement: Supplementary data [file bmjopen-2021-055501supp001.pdf]

Appendix

Appendix table 1: Crude prevalence of factors associated with LED

		OUTCOME						
	N	LED prevalence (95% CI) n=194	Absent pulse prevalence (95% CI) n=54	Amputation Prevalence (95% CI) n=7	Ulcer Prevalence (95% CI) n=13	Claudication Prevalence (95% CI) n=81	Neuropathy Prevalence (95% CI) n=103	ABPI<0.9 Prevalence (95% CI) n=15
**Total**								
	806	24.1 (21.2—27.2)	6.7 (5.1—8.7)	0.9 (0.3—1.8)	1.1 (0.4— 1.8)	10 (8.1—12.3)	12.8 (10.6—15.3)	1.9 (1.0—3.1)
**Age**								
<50	346	15.9 (12.2—20.2)	4.9 (2.9—7.8)	0.3 (0.0—1.6)	2.3 (1.0—4.5)	9.0 (6.2—12.5)	6.1 (3.8—9.1)	0.3 (0.0—1.6)
50-60	239	27.6 (22.0—33.7)	6.7 (3.9—10.6)	2.1 (0.7—4.8)	0.8 (0.1—3.0)	12.6 (8.6—17.4)	15.1 (10.8—20.2)	0.8 (0.1—3.0)
>60	221	33.0 (26.9—39.7)	9.5 (6.0—14.2)	0.5 (0.0—2.5)	1.4 (0.3—3.9)	9.0 (5.6—13.6)	20.8 (15.7—26.8)	5.4 (2.8—9.3)
**Sex**								
Male	431	15.8 (12.5—19.6)	5.8 (3.8—8.4)	0.5 (0.1—1.7)	1.4 (0.5—3.0)	4.2 (2.5—6.5)	10.2 (7.5—13.5)	0.9 (0.3—2.4)
Female	375	33.6 (28.8— 38.6)	7.7 (5.2—10.9)	1.3 (0.4—3.1)	1.9 (0.8— 3.8)	16.8 (13.2—21.0)	15.7 (12.2—19.8)	2.9 (1.5—5.2)
**Residence**								
Rural	457	17.5 (14.1—21.3)	9.0 (6.5—12.0)	1.1 (0.4—2.5)	1.8 (0.8—3.4)	2.2 (1.1— 4.0)	13.8 (10.8—17.3)	1.8 (0.8—3.4)
Urban	349	32.7 (27.8—37.9)	3.7 (2.0—6.3)	0.6 (0.1— 2.1)	1.4 (0.5—3.3)	20.3 (16.2—25.0)	11.5 (8.3—15.3)	2.0 (0.8—4.1)
**Wealth quintiles**								
poorest	108	32.4 (16.2—30.2)	13.0 (7.3—20.8)	0.9 (0.02—5.1)	0.9 (0.02—5.1)	7.5 (2.6—12.9)	25 (17.2—34.3)	5.6 (2.1—11.7)

second	150	22.7 (16.2—30.2)	5.3 (2.3—10.2)	0.0 (0—2.4)	0.6 (0.02—3.7)	7.3 (3.7—12.7)	14.0 (8.9—20.6)	2.0 (0.4—5.7)
third	139	15.8 (10.2—23.0)	6.5 (3.0—11.9)	0.0 (0—2.6)	1.4 (0.2—5.1)	6.5 (3.0—11.9)	8.6 (4.5—14.6)	0.7 (0.0—3.9)
fourth	191	24.1 (18.2—30.8)	4.7 (2.2—8.8)	2.1 (0.6—5.3)	0.5 (0.0—2.9)	13.1 (8.7—18.7)	11.0 (6.9—16.3)	1.6 (0.3—4.5)
wealthiest	218	26.1 (20.4—32.5)	6.4 (3.6—10.5)	0.9 (0.1—3.3)	3.7 (1.6—7.1)	13.3 (9.1—18.5)	10.1 (6.4—15.9)	0.9 (0.1—3.3)
Marital status								
Single	209	34.0 (27.6—40.8)	10.0 (6.3—15.0)	1.0 (0.1—3.4)	1.9 (0.5—4.8)	12.4 (8.2—17.7)	18.7 (13.6—24.6)	3.8 (1.7—7.4)
married	587	20.6 (17.4—24.1)	5.5 (3.8—7.7)	0.8 (0.3—1.9)	1.5 (0.7—2.8)	9.2 (7.0—11.8)	10.7 (8.4—13.5)	1.2 (0.5—2.4)
Educational achievement								
0-5 years primary education	164	25 (18.6—32.3)	7.9 (4.3—13.2)	0 (0.0—2.2)	1.2 (0.1—4.3)	6.7 (3.4—11.7)	16.5 (11.1—23.0)	4.3 (1.7—8.6)
Standard 6-8	329	25.5 (20.9—30.6)	6.7 (4.2—9.9)	0.6 (0.1—2.2)	1.5 (0.5—3.5)	11.9 (8.6—15.8)	12.5 (9.1—16.5)	2.1 (0.9—4.3)
Secondary	230	21.7 (16.6—27.6)	6.1 (3.4—10.0)	1.7 (0.5—4.4)	2.6 (1.0—5.6)	8.2 (5.0—12.6)	12.2 (8.2—17.1)	0.4 (0.0—2.4)
Post-secondary	83	22.9 (14.4—33.4)	6.0 (2.0—13.5)	1.2 (0.0—6.5)	0.0 (0—4.3)	14.5 (7.7—23.9)	8.4 (3.5—16.6)	0.0 (0.0—4.3)
occupation								
homemaker	237	37.1 (31.0—43.6)	7.2 (4.2—11.2)	1.7 (0.5—4.3)	2.5 (0.9—5.4)	18.6 (13.8—24.1)	15.6 (11.2—20.9)	4.2 (2.0—7.6)
Farming/ fishing	296	15.5 (11.6—20.2)	9.1 (6.1—13.0)	0.3 (0.0—1.9)	1.0 (0.2—2.9)	1.7 (0.6—3.9)	12.8 (9.2—17.9)	1.7 (0.6—3.9)
employed	273	22.0 (17.2—27.4)	3.7 (1..8—6.6)	0.7 (0.0—2.6)	1.5 (0.4—3.7)	11.7 (8.2—16.1)	10.3 (6.9—14.5)	0.0 (0—1.3)
Indoor fire								
none	42	7.1 (1.5—19.5)	4.8 (0.6—16.2)	0.0 (0—8.4)	2.4 (0.0—12.6)	4.8 (0.6—16.2)	0.0 (0—8.4)	0.0 (0—8.4)
Fire usually lit Inside the house	39	30.8 (17.0—47.6)	7.7 (1.6—20.9)	0.0 (0—9.0)	2.6 (0.0—13.5)	17.9 (7.5—33.5)	12.8 (4.3—27.4)	0.0 (0—9.0)
Fire usually lit in a separate	725	24.7 (21.6—28.0)	6.8 (5.0—8.8)	1.0 (0.4—2.0)	1.5 (0.8—2.7)	9.9 (7.9—12.3)	13.5 (11.1—16.2)	2.1 (1.2—3.4)

kitchen area								
**Any Smoking history**								
no	487	29.4 (25.4—33.6)	7.6 (5.4—10.3)	1.2 (0.5—2.7)	1.8 (0.8—3.5)	14.0 (11.0—17.4)	14.4 (11.4—17.8)	2.1 (1.0—3.7)
yes	319	16.0 (12.1—20.5)	5.3 (3.1—8.4)	0.3 (0.0—1.7)	1.3 (0.3—3.2)	4.1 (2.2—6.9)	10.3 (7.2—14.2)	1.6 (0.5—3.6)
**Diabetes**								
Non-diabetic	502	19.7 (16.3—23.5)	6.2 (4.2—8.7)	0 (0—0.7)	1.2 (0.4—2.6)	8.2 (5.9—10.9)	10.2 (7.7—13.1)	1.4 (0.6—2.9)
diabetic	273	33.7 (28.1—39.6)	7.7 (4.8—11.5)	2.6 (1.0—5.2)	2.2 (0.8—4.7)	13.9 (10.0—18.6)	18.7 (14.2—23.8)	2.9 (1.3—5.7)
Missing data	31	9.7 (2.0—25.8)	6.5 (0.8—21.4)	0.0 (0—11.2)	3.2 (0.0–16.7)	6.5 (0.8—21.4)	3.2 (0.1—16.7)	0.0 (0.0—11.2)
**Dyslipidaemia**								
no	413	22.8 (18.8—27.1)	6.5 (4.4—9.4)	0.7 (0.2—2.1)	1.2 (0.4—2.8)	10.4 (7.6—13.8)	10.7 (7.8—14.0)	2.4 (1.2—4.4)
yes	344	26.5 (21.9—31.5)	7.0 (4.5—10.2)	1.2 (0.3—3.0)	1.7 (0.6—3.8)	10.2 (7.2—13.9)	15.7 (12.0—20.0)	1.5 (0.5—3.4)
Missing data	49	18.4 (8.8—32.0)	6.1 (1.3—16.9)	0.0 (0.0—7.3)	4.1 (0.5—14.0)	6.1 (1.3—16.9)	10.2 (3.4—22.2)	0.0 (0.0—7.3)
**HIV**								
negative	413	23.0 (19.0—27.4)	7.5 (5.2—10.5)	0.7 (0.2—2.1)	1.2 (0.4—2.8)	10.0 (7.2—13.2)	12.1 (9.1—15.6)	2.4 (1.2—4.4)
positive	247	23.9 (18.7—29.7)	6.1 (3.4—9.8)	0.4 (0.0—2.2)	2.0 (0.7—4.7)	10.1 (6.7—14.6)	12.6 (8.7—`17.3)	0.8 (0.1—2.9)
unknown	146	27.4 (20.3—35.4)	5.5 (2.4—10.5)	0.0 (0—2.5)	0.7 (0.0—3.8)	10.3 (5.9—16.4)	15.1 (9.7—21.9)	2.7 (0.8—6.9)
**hypertension**								
Not-hypertensive	324	21.9 (17.6—26.8)	4.6 (2.6—7.5)	0.9 (0.2—2.7)	2.2 (0.9—4.4)	9.3 (6.3—13.0)	12.7 (9.2—16.8)	0.6 (0.1—2.2)
hypertensive	482	25.5 (21.7—29.7)	8.1 (5.8—10.9)	0.8 (0.2—2.1)	1.2 (0.5—2.7)	10.6 (8.0—13.7)	12.9 (10.0—16.2)	2.7 (1.4—4.6)
**obesity**								

no	634	20.8 (17.7—24.2)	5.8 (4.1—8.0)	0.6 (0.2—1.6)	1.6 (0.8—2.9)	7.3 (5.4—9.6)	11.8 (9.4—14.6)	1.9 (1.0—3.3)
yes	172	36.0 (28.9—43.7)	9.9 (5.9—15.4)	1.7 (0.4—5.0)	1.7 (0.4—5.0)	20.3 (14.6—27.1)	16.3 (11.1—22.7)	1.7 (0.4—5.0)
Number of risk factors								
2	201	14.9 (10.3—20.6)	3.0 (1.1—6.4)	1.0 (0.1—3.5)	1.1 (0.1–3.5)	7.5 (4.2—12.0)	6.5 (3.4—10.8)	0.5 (0.0—2.7)
3	342	23.1 (18.7—27.9)	6.7 (4.3—9.9)	0.3 (0.0—1.6)	2.0 (0.8—4.2)	8.8 (6.0—12.3)	12.0 (8.7—15.9)	2.6 (1.2—4.9)
4	204	32.4 (26.0 —39.2)	9.3 (5.7—14.2)	0.5 (0.0—2.7)	1.0 (0.5—4.9)	14.7 (10.1—20.3)	18.1 (13.1—24.1)	2.0 (0.5—4.9)
≥5	59	32.2 (20.6—45.6)	10.2 (3.8—20.8)	5.1 (1.1—14.1)	0.0 (0.0—6.1)	10.2 (3.8—20.8)	20.3 (11.0—32.8)	1.7 (0.0—9.1)
Prevalence of outcomes by risk factors								
No diabetes and no hypertension	193	14.0 (25.6—42.4)	4.1 (1.8—8.0)	0.0 (0.0—1.9)	1.6 (0.3—4.4)	4.7 (2.2—8.7)	9.3 (5.6—14.3)	0.0 (0.0—1.9)
Diabetes but no hypertension	131	33.6 (25.6—42.4)	5.3 (2.2—10.7)	2.3 (0.5 –6.5)	3.1 (0.8—7.6)	16.0 (10.2—23.5)	17.6 (11.5—25.2)	1.5 (0.2—5.4)
Hypertension but no diabetes	340	22.1 (17.8—26.8)	7.4 (4.8—10.7)	0.0 (0.0 —1.1)	1.2 (0.3—3.0)	10 (7.0—13.7)	10.0 (7.0—13.7)	2.1 (0.8—4.2)
Hypertension and diabetes	142	33.8 (26.1—42.2)	9.9 (5.5—16.0)	2.8 (0.8 —7.1)	1.4 (0.2 —5.0)	12.0 (7.1—18.5)	19.7 (13.5—27.2)	4.2 (1.6—9.0)

Appendix table 2: full regression model of factors associated with Lower extremity disease

	Odds Ratio	Std. Err.	z	P>z	95% confidence interval	Lr test p value
**Age**						
<50	1	ref.				0.0012
50-60	1.91	0.4	2.8	0.0	1.22—2.99	
>60	2.33	0.6	3.4	0.0	1.43—3.8	
**sex**						
Male	1.0	ref.				0.0574
Female	1.69	0.5	1.9	0.1	0.98—2.9	
**site**						
Karonga	1.0	ref.				0.0296
Lilongwe	1.76	0.5	2.2	0.0	1.05—2.94	
**Wealth quintiles**						
poorest	1.93	0.7	1.9	0.1	0.99—3.77	0.3322
second	1.72	0.6	1.7	0.1	0.91—3.27	
third	1.0	ref.				
fourth	1.43	0.4	1.2	0.2	0.78—2.66	
wealthiest	1.61	0.5	1.5	0.1	0.85—3.05	
**Marital status**						
single	1.0	ref.				0.0862
married	0.69	0.1	-1.7	0.1	0.45—1.05	
**Highest attained education**						
years primary education	0.75	0.2	-1.2	0.2	0.46—1.22	0.2630
standard 6-8	1.0	ref.				
secondary	0.78	0.2	-1.1	0.3	0.49—1.22	
post-secondary	0.54	0.2	-1.7	0.1	0.27—1.08	
**Occupation**						
homemaker	1.62	0.5	1.7	0.1	0.93—2.83	0.2026
farming/fishing	1.0	ref.				
employed	1.22	0.3	0.7	0.5	0.7—2.13	
**Indoor smoke**						
No	0.19	0.1	-2.5	0.0	0.05—0.69	0.0105
Yes, inside the house	0.88	0.3	-0.3	0.7	0.4—1.91	
Yes, in a separate kitchen area	1.0	ref.				
**any history of smoking**						
no	1.0	ref.				0.8995
yes	0.96	0.3	-0.1	0.9	0.5—1.84	
**Diabetes**						
No	1.0	ref.				0.3532
Yes	0.95	0.3	-0.2	0.9	0.48—1.86	

Unknown	0.3	0.3	-1.4	0.2	0.06—1.61	
**Dyslipidaemia**						
No	1.0	ref.				0.8425
Yes	0.82	0.3	-0.5	0.6	0.41—1.66	
missing data	0.87	0.5	-0.3	0.8	0.28—2.67	
**HIV status**						
Negative	1.0	ref.				0.4490
Positive	0.98	0.3	-0.1	0.9	0.51—1.88	
Unknown	1.35	0.3	1.2	0.2	0.83—2.2	
**hypertension**						
No	1.0	ref.				0.1768
Yes	0.65	0.2	-1.3	0.2	0.35—1.22	
**obesity**						
No	1.0	ref.				0.9754
Yes	0.99	0.4	0.0	1.0	0.49—2.0	
**CVDRF**						
2	0.62	0.2	-1.3	0.2	0.3—1.3	0.4812
3	1.0	ref.				
4	1.49	0.5	1.1	0.3	0.72—3.07	
≥5	1.54	1.1	0.6	0.5	0.4—5.98	

Stratified analysis for lower extremity disease

Appendix table 3: stratification by sex

	Male	Female
**Age**		
<50	(ref)	(ref)
50-60	1.96 (0.91—4.22)	1.97 (1.08—3.6)
>60	2.41 (1.08—5.37)	2 (1.0*—4.01)
**site**		
Karonga	(ref)	(ref)
Lilongwe	1.84 (0.79—4.31)	1.65 (0.82—3.34)
**Wealth quintiles**		
poorest	1.92 (0.6—6.19)	2.04 (0.86—4.88)
second	2.32 (0.87—6.18)	1.4 (0.58—3.39)
third	(ref)	(ref)
fourth	1.74 (0.65—4.64)	1.51 (0.66—3.46)
wealthiest	1.23 (0.42—3.6)	2.03 (0.87—4.72)
**Marital status**		
single	(ref)	(ref)
married	0.39 (0.18—0.86)	0.81 (0.47—1.39)
**Highest attained education**		
0-5 years prima..	1.01 (0.41—2.48)	0.69 (0.36—1.3)
standard 6-8	(ref)	(ref)
secondary	0.86 (0.42—1.73)	0.68 (0.36—1.28)
post-secondary	0.62 (0.19—2.06)	0.43 (0.17—1.09)
**Occupation**		
homemaker	1.09 (0.44—2.72)	2.19 (0.98—4.89)
farming/fishing	(ref)	(ref)
employed	0.91 (0.4—2.06)	1.87 (0.8—4.38)
**Indoor fire**		
No	(empty)	0.28 (0.07—1.17)
Yes, inside the house	0.66 (0.17—2.59)	1.06 (0.39—2.92)
Yes, but in a separate kitchen area.	(ref)	(ref)
**History of smoking**		
no	(ref)	(ref)
yes	0.79 (0.34—1.8)	1.83 (0.52—6.47)
**Diabetes**		
No	(ref)	(ref)
Yes	1.89 (0.71—5.01)	0.38 (0.13—1.18)
Unknown	0.48 (0.05—5.04)	0.18 (0.01—2.69)
**Dyslipidaemia**		

* Rounded up from .9989416

No	(ref)	(ref)
Yes	0.91 (0.32—2.59)	0.48 (0.16—1.49)
missing data	1.05 (0.18—6.21)	0.6 (0.12—2.97)
HIV status		
Negative	(ref)	(ref)
Positive	1.81 (0.74—4.41)	0.38 (0.13—1.13)
Unknown	1.69 (0.8—3.58)	0.93 (0.46—1.85)
hypertension		
No	(ref)	(ref)
Yes	0.52 (0.22—1.24)	0.39 (0.13—1.19)
obesity		
No	(ref)	(ref)
Yes	2.13 (0.64—7.07)	0.5 (0.17—1.48)
CVDRF		
2	0.68 (0.23—1.97)	0.45 (0.14—1.47)
3	(ref)	(ref)
4	1.37 (0.48—3.94)	2.59 (0.82—8.2)
≥5	0.76 (0.09—6.61)	6.48 (0.73—57.42)

Appendix table 4: Stratification by Sex and history of smoking

Variables and variable categories	Male non-smoker	Male smoker	Female non-smoker	Female current smokers[†]
Age				
<50	(ref)	(ref)	(ref)	
50-60	2.74 (0.5—14.91)	1.43 (0.53—3.85)	2.1 (1.15—3.85)	
>60	4.53 (0.62—32.88)	1.86 (0.68—5.06)	1.95 (0.96—3.94)	
Site				
Karonga	(ref)	(ref)	(ref)	
Lilongwe	0.63 (0.12—3.41)	3.38 (1.07—10.62)	1.82 (0.88—3.78)	
Wealth Quintile				
poorest	6.26 (0.26—150.93)	1.58 (0.35—7.09)	1.71 (0.7—4.18)	
second	4.03 (0.18—89)	2.85 (0.9—9.05)	1.38 (0.56—3.43)	
third	(ref)	(ref)	(ref)	
fourth	4.67 (0.28—76.66)	1.27 (0.41—3.99)	1.24 (0.54—2.87)	
wealthiest	14.33 (0.91—225.09)	0.72 (0.16—3.21)	1.62 (0.69—3.81)	
Marital status				
single	(ref)	(ref)	(ref)	
married	0.04 (0.0—0.56)	0.58 (0.21—1.58)	0.91 (0.52—1.58)	
Education				
years primary education	4.93 (0.17—139.91)	0.88 (0.33—2.35)	0.64 (0.33—1.24)	
standard 6-8	(ref)	(ref)	(ref)	
secondary	3.02 (0.57—15.97)	0.62 (0.24—1.57)	0.78 (0.41—1.49)	
post-secondary	1.35 (0.19—9.71)	0.75 (0.07—8.19)	0.47 (0.19—1.18)	
Occupation				
homemaker	1.82 (0.29—11.26)	0.72 (0.2—2.6)	2.09 (0.9—4.88)	
farming/fishing	(ref)	(ref)	(ref)	
employed	0.43 (0.07—2.8)	1.1 (0.38—3.23)	1.68 (0.69—4.08)	
Indoor smoke				
No	(empty)	(empty)	(empty)	
Yes, inside the house	(empty)	0.82 (0.17—3.98)	1.02 (0.37—2.8)	
a separate kitchen area	(ref)	(ref)	(ref)	
diabetes				
No	(ref)	(ref)	(ref)	
Yes	1.57 (0.11—22.08)	3.79 (0.94—15.32)	0.35 (0.1—1.22)	
Unknown	(empty)	0.58 (0.02—16.66)	0.24 (0.02—3.85)	
Dyslipidaemia				
No	(ref)	(ref)	(ref)	
Yes	0.17 (0.01—3.9)	0.79 (0.2—3.14)	0.45 (0.13—1.53)	
missing data	0.28 (0.01—5.91)	0.7 (0.03—14.21)	0.57 (0.1—3.22)	
HIV status				

[†] No observations

Negative	(ref)	(ref)	(ref)	
Positive	1.82 (0.12—28.15)	2 (0.65—6.15)	0.41 (0.13—1.33)	
Unknown	2.01 (0.41—9.92)	1.74 (0.65—4.71)	0.99 (0.48—2.02)	
hypertension				
No	(ref)	(ref)	(ref)	
Yes	0.65 (0.06—6.93)	0.35 (0.1—1.15)	0.37 (0.11—1.25)	
obesity				
No	(ref)	(ref)	(ref)	
Yes	0.59 (0.04—9.2)	6.62 (1.06—41.46)	0.52 (0.16—1.72)	
CVDRF				
2	0.66 (0.04—11.7)	0.46 (0.11—1.85)	0.41 (0.11—1.49)	
3	(ref)	(ref)	(ref)	
4	20.29 (0.76—539.61)	0.51 (0.11—2.41)	2.67 (0.76—9.37)	
5	62.07 (0.17—22604.06)	0.26 (0.01—7.99)	6.71 (0.61—73.19)	

Appendix table 5: Stratification by obesity and wealth quintiles

Variables and variable categories	non obese WQ1	Non obese wq2	Non obese wq3	Non obese WQ4	Non obese WQ5	Obese WQ1[‡]	Obese WQ2[§]	Obese WQ3	Obese WQ4	Obese WQ5
**Age**										
<50	(ref)	(ref)	(ref)	(ref)	(ref)			1.45E-31 (.—.)	(ref)	(ref)
50-60	43.49 (1.51—1248.98)	4.57 (1.07—19.58)	0.8 (0.11—6.03)	2.63 (0.77—8.93)	0.85 (0.23—3.06)			5.54E-47 (.—.)	1.44 (0.07—30.58)	3.48 (0.5—24.14)
>60	12.11 (0.89—164.53)	4.43 (1.03—19.09)	0.42 (0.04—4.69)	3.82 (1.03—14.23)	2.18 (0.49—9.74)				1.91 (0.06—57.17)	2.65 (0.33—21.42)
**sex**										
Male	(ref)	(ref)	(ref)	(ref)	(ref)			(ref)	(empty)	(ref)
Female	3.29 (0.44—24.85)	4.32 (0.64—28.94)	0.62 (0.03—13.88)	1.26 (0.32—4.93)	1.67 (0.45—6.18)			8.6E+107 (.—.)	(omitted)	1.9 (0.19—18.76)
**site**										
Karonga	(ref)	(ref)	(ref)	(ref)	(ref)			(ref)	(empty)	(ref)
Lilongwe	12.72 (0.89—182.11)	3.95 (0.65—24.04)	2.64 (0.35—19.71)	2.49 (0.73—8.5)	0.53 (0.11—2.43)			2.6E+15 (.—.)	(omitted)	5.05 (0.13—202.03)
**Marital status**										
single	(ref)	(ref)	(ref)	(ref)	(ref)			(ref)	(ref)	(ref)
married	1.39 (0.29—6.64)	0.95 (0.28—3.28)	0.29 (0.04—2.15)	0.5 (0.14—1.7)	0.64 (0.14—2.9)			8.6E+107 (.—.)	0.53 (0.03—8.15)	0.14 (0.02—1.04)
**Highest attained education**										
0—5 years primary education	11.09 (1.5—82.05)	0.89 (0.28—2.81)	0.34 (0.02—4.67)	0.76 (0.15—3.75)	2.09 (0.21—21.31)			8.1E-78 (.—.)	0.26 (0.0—16.1)	0.3 (0.02—5.47)
standard 6-8	(ref)	(ref)	(ref)	(ref)	(ref)			(ref)	(ref)	(ref)
secondary	57.88 (1.61—2080.34)	1.4 (0.3—6.59)	1.42 (0.27—7.35)	0.71 (0.24—2.11)	2.24 (0.63—7.94)			(empty)	0.34 (0.01—8.42)	0.49 (0.07—3.34)
post-secondary	42.93 (0.52—3528.5)	(empty)	(empty)	1.37 (0.08—24.48)	1.35 (0.27—6.7)				(empty)	0.37 (0.06—2.51)

‡ No observations

§ No observations

occupation										
homemaker	0.8 (0.11—5.7)	0.61 (0.12—3.13)	21.78 (0.94—504.37)	0.79 (0.15—4.1)	5.97 (0.83—42.82)			4.7E+61 (.—.)	2.00 (0.04—100.23)	0.73 (0.0—155.92)
farming/fishing	(ref)	(ref)	(ref)	(ref)	(ref)			(ref)	(empty)	(ref)
employed	2.15 (0.29—16.04)	1.43 (0.23—9.04)	13.15 (0.9—192.03)	0.66 (0.17—2.58)	1.33 (0.23—7.83)			1.5E+154 (.—.)	(omitted)	0.19 (0.0—34.71)
Indoor smoke										
No	(empty)	0.26 (0.02—3.55)	(empty)	(empty)	(empty)				(empty)	0.75 (0.04—15.21)
Yes, inside the house	(empty)	2.91 (0.27—30.92)	(empty)	3.89 (0.65—23.19)	0.12 (0.01—1.5)				(empty)	1.64 (0.18—15.18)
Yes, in a separate kitchen area	(ref)	(ref)	(ref)	(ref)	(ref)			(ref)	(ref)	(ref)
History of Smoke										
no	(ref)	(ref)	(ref)	(ref)	(ref)			(ref)	(ref)	(ref)
yes	0.29 (0.0—17.25)	3.07 (0.33—28.35)	0.12 (0.0—4.39)	0.94 (0.17—5.11)	0.26 (0.04—1.65)			4.1E+169 (.—.)	(empty)	0.54 (0.01—23.36)
Diabetes										
No	(ref)	(ref)	(ref)	(ref)	(ref)			(ref)	(ref)	(ref)
Yes	0.13 (0—16.29)	2.43 (0.29—20.13)	0.17 (0.0—9.5)	2.54 (0.54—12)	1.15 (0.19—7.16)			1.8E+46 (.—.)	0.07 (0.0—14.48)	1.15 (0.09—14.87)
Unknown	(empty)	(empty)	2.1E-08 (0.1—22.1)	7.2E+05 (0.0—.)	(empty)				(empty)	
Dyslipidaemia										
No	(ref)	(ref)	(ref)	(ref)	(ref)			(ref)	(ref)	(ref)
Yes	0.06 (0.0—4.64)	0.58 (0.07—4.84)	0.06 (0.0—1.92)	3.03 (0.53—17.14)	0.06 (0.0—1.19)			2.6E+07 (.—.)	0.31 (0.0—22.6)	0.93 (0.03—26.3)
missing data	(empty)	(empty)	7.7E+07 (0.0—.)	2.0E-06 (0.0—.)	0.18 (0.02—2.11)			(empty)	(empty)	0.48 (0—64.61)
HIV status										
Negative	(ref)	(ref)	(ref)	(ref)	(ref)			(ref)	(ref)	(ref)
Positive	0.08 (0.0—6.74)	1.15 (0.17—7.63)	0.12 (0.0—4.26)	1.94 (0.37—10.03)	1.41 (0.24—8.31)			1.45E-31 (.—.)	0.24 (0—22.56)	1.98 (0.14—27.98)
Unknown	2.1 (0.33—13.38)	1.66 (0.4—6.81)	1.33 (0.08—22.1)	2.04 (0.5—8.3)	1.63 (0.27—9.77)			1.45E-31 (.—.)	0.39 (0.02—7.7)	1.79 (0.23—13.66)

Hypertension										
No	(ref)	(ref)	(ref)	(ref)	(ref)			(ref)	(ref)	(ref)
Yes	0.11 (0.0—9.9)	0.7 (0.1—4.87)	0.03 (0.0—0.91)	0.85 (0.21—3.47)	0.6 (0.09—4.13)			1.81E+46 (.—.)	0.33 (0.01—19.43)	1.41 (0.14—14.27)
CVDRF										
2	0.03 (0.0—1.88)	0.8 (0.1—6.22)	0.0 (0.0—0.29)	1.59 (0.28—9.08)	1.29 (0.17—9.96)					
3	(ref)	(ref)	(ref)	(ref)	(ref)			(ref)	(ref)	(ref)
4	49.06 (0.47—5153.28)	1.87 (0.18—18.98)	2.7 (0.06—112.27)	0.92 (0.18—4.78)	16.97 (1.14—253.16)			1.81E+46 (.—.)	1.17 (0.0—154.19	0.58 (0.02—18.29)
≥5	(empty)	(empty)	(empty)	(empty)	(empty)			(omitted)	6.75 (0.0—24323.16)	0.48 (0.0—105.76)

Appendix table 6: Stratification by highest attained education and obesity

Variables and variable categories	Non-obese & 0-5 years elementary education	Non-obese & 6-8 elementary education	Non obese & secondary education	Non-obese & post-secondary education[**]	Obese & 0-5 years elementary education[††]	Obese & 6-8 years elementary education	Obese & secondary education	Obese & post-secondary education[‡‡§§]
**Age**								
<50	(ref)	(ref)	(ref)	(ref)	(ref)	(ref)	(ref)	(ref)
50-60	10.76 (1.45—79.81)	1.33 (0.57—3.15)	2.18 (0.66—7.22)	.	2.07e+19 (.—.)	2.96 (0.6—14.49)	12.52 (0.3—525.67)	1.14E-57 (.—.)
>60	5.85 (0.9—38)	1.65 (0.63—4.29)	6.74 (1.83—24.92)	.	264.2 (.—.)	3.12 (0.43—22.52)	5.03 (0.02—1686.17)	1.64E+56 (.—.)
**Sex**								
Male	(ref)	(ref)	(ref)	(ref)	(omitted)	(ref)	(ref)	(ref)
Female	7.38 (0.9—60.6)	2.73 (0.77—9.71)	1.28 (0.35—4.69)	.		0.47 (0.03—8.69)	0.84 (0.02—45.4)	4.96E+56 (.—.)
**Site**								
Karonga	(ref)	(ref)	(ref)	(empty)	(ref)	(ref)	(ref)	(ref)
Lilongwe	5.38 (0.91—31.62)	3.06 (1.12—8.35)	0.88 (0.26—3.04)	(omitted)	3.6E+22 (.—.)	2.01 (0.23—17.74)	1.06 (0.01—97.43)	3.8E+107 (.—.)
**Wealth quintiles**								
poorest	25.05 (1.9—330.22)	1.59 (0.42—6.0)	1.72 (0.23—12.74)	.	1.8E+21 (.—.)	0.3 (0.03—3.47)	(empty)	(empty)
second	7.82 (0.71—86.4)	3.3 (1.02—10.68)	1.56 (0.28—8.67)		3.1E+42 (.—.)	0.37 (0.03—4.01)	13.09 (0.03—5949.07)	(empty)
third	(ref)	(ref)	(ref)	(empty)	(ref)	(ref)	(ref)	(empty)
fourth	2.4 (0.15—37.21)	1.85 (0.64—5.37)	1.96 (0.49—7.89)	(empty)	3.9E-21 (.—.)	0.22 (0.02—2.3)	0.24 (0—38.03)	(empty)
wealthiest	2.09 (0.1—42.23)	0.84 (0.24—2.92)	3.39 (0.75—15.3)	(omitted)	7.8 (.—.)	0.44 (0.04—4.71)	11.69 (0.09—1444.24)	(omitted)
**Marital status**								

[**] Site, wealth quintiles and occupation omitted because of collinearity
[††] Sex, occupation and history of smoking omitted because of collinearity
[‡‡] Wealth and occupation have no observations
[§§] History of smoking and CVDRF omitted because of collinearity

single	(ref)	(ref)	(ref)	(ref)	(ref)	(ref)	(ref)	(ref)
married	1.11 (0.28—4.43)	1.32 (0.54—3.23)	0.31 (0.11—0.91)	0.0 (.—.)	4.6E-22 (.—.)	0.69 (0.11—4.38)	0.13 (0.01—2.61)	7.38E+17
occupation								
homemaker	2.51 (0.56—11.17)	1.66 (0.54—5.08)	0.69 (0.17—2.84)	0.0 (.—.)	4.0 e+19 (.—.)	2.78 (0.29—26.69)	0.3 (0.01—12.69)	1.04E+95 (.—.)
farming/fishing	(ref)	(ref)	(ref)	(empty)	(empty)	(ref)	(empty)	(empty)
employed	1.87 (0.27—12.76)	1.83 (0.67—4.99)	0.5 (0.12—2.03)	1 (omitted)	1 **(omitted)**	1.71 (0.15—19.93)	1 (omitted)	(omitted)
Indoor smoke								
No	0.96 (0.04—21.49)	(Empty)	(Empty)	(empty)	(empty)	(empty)	(empty)	4.62E+74 (.—.)
Yes, inside the house	1.93 (0.21—17.91)	1.88 (0.42—8.4)	0.11 (0.01—1.59)	(empty)		0.93 (0.05—18.44)	5.99 (0.04—988.69)	4.72E+94 (.—.)
Yes, in a separate kitchen area	(ref)	(ref)	(ref)	(ref)	(ref)	(ref)	(ref)	(ref)
Smoking history								
no	(ref)	(ref)	(ref)	(ref)	(ref)	(ref)	(ref)	(ref)
yes	10.25 (0.96—109.45)	1.29 (0.29—5.66)	0.25 (0.05—1.3)	0.0 (.—.)	(empty)	0.35 (0.01—14.51)	9.43E+07 (0.0—.)	(empty)
Diabetes								
No	(ref)	(ref)	(ref)	(ref)	(ref)	(ref)	(ref)	(ref)
Yes	1.06 (0.1—11.91)	0.88 (0.21—3.71)	1.28 (0.26—6.33)	0.0 (.—.)	1.2E-20 (.—.)	0.53 (0.05—5.5)	1.4e+07 (0.0—.)	2.0E+163 (.—.)
Unknown	(empty)	0.22 (0.01—5.99)	(empty)	(empty)		(empty)	(empty)	
Dyslipidaemia								
No	(ref)	(ref)	(ref)	(ref)	(ref)	(ref)	(ref)	(ref)
Yes	1.05 (0.12—9.23)	0.43 (0.11—1.75)	2.43 (0.38—15.56)	0.0 (.—.)	1.4E+23 (.—.)	1 (0.05—21.22)	32.63 (0.17—6363.24)	1.5E-127 (.—.)
missing data	0.41 (0.02—7.53)	3.39 (0.18—65.18)	0.14 (0.01—2.05)	(empty)		(empty)	109.64 (0.06—205197.3)	(empty)
HIV status								
Negative	(ref)	(ref)	(ref)	(ref)	(ref)	(ref)	(ref)	(ref)
Positive	0.85 (0.08—9.02)	0.6 (0.14—2.55)	2.5 (0.62—10.07)	.	5.5E+23 (.—.)	0.88 (0.05—17.25)	24.16 (0.59—992.53)	1.6E+105 (.—.)

Unknown	0.87 (0.15—5.16)	1.34 (0.52—3.45)	2.37 (0.6—9.4)	.	(empty)	0.75 (0.14—3.89)	157.01 (0.5—49479.48)	8.25E-77 (.—.)
Hypertension								
No	(ref)	(ref)	(ref)	(ref)	(ref)	(ref)	(ref)	(ref)
Yes	1.58 (0.23—10.99)	0.51 (0.13—1.97)	0.68 (0.13—3.57)	0.0 (.—.)	4.4E-38 (.—.)	0.44 (0.04—5.13)	2.37 (0.06—95.27)	6.1E+132 (.—.)
CVDRF								
2	0.53 (0.05—5.26)	0.18 (0.04—0.8)	2.39 (0.45—12.74)	.				(ref)
3	(ref)	(ref)	(ref)	(ref)	(ref)	(ref)	(ref)	2.43E+94 (.—.)
4	2.88 (0.28—29.86)	2.42 (0.57—10.31)	0.62 (0.1—3.8)	.	1.4E-22 (.—.)	0.99 (0.04—23.46)	27.92 (0.04—17774.42)	(omitted)
≥5		(empty)	21.02 (0.37—1193.6)	(empty)	4.4e-07 (.—.)	1.95 (0.01—423.84)	1.44 (0.0—2718.99)	1.14E-57 (.—.)
