# Supplementary material for: Prevalence and risk factors of lower extremity disease in high risk groups in Malawi: a stratified cross-sectional study
Source: BMJ Open. 2022 Aug 10;12(8):e055501. doi: 10.1136/bmjopen-2021-055501 (PMC9379496; doi:10.1136/bmjopen-2021-055501)
Supplement: Author's manuscript [file bmjopen-2021-055501.draft_revisions.pdf]

BMJ Open is committed to open peer review. As part of this commitment we make the peer review history of every article we publish publicly available.

When an article is published we post the peer reviewers' comments and the authors' responses online. We also post the versions of the paper that were used during peer review. These are the versions that the peer review comments apply to.

The versions of the paper that follow are the versions that were submitted during the peer review process. They are not the versions of record or the final published versions. They should not be cited or distributed as the published version of this manuscript.

BMJ Open is an open access journal and the full, final, typeset and author-corrected version of record of the manuscript is available on our site with no access controls, subscription charges or pay-per-view fees (http://bmjopen.bmj.com).

If you have any questions on BMJ Open's open peer review process please email info.bmjopen@bmj.com

BMJ Open

The prevalence and risk factors of lower extremity disease in high risk groups in Malawi: a stratified cross-sectional study

Journal:	*BMJ Open*
Manuscript ID	bmjopen-2021-055501
Article Type:	Original research
Date Submitted by the Author:	19-Jul-2021
Complete List of Authors:	Kasenda, Stephen; Malawi Epidemiology and Intervention Research Unit<br>Crampin, Amelia; Malawi Epidemiology and Intervention Research Unit; London School of Hygiene & Tropical Medicine<br>Davies, Justine ; King's College London, Global Health<br>Malava, Jullita; Malawi Epidemiology and Intervention Research Unit<br>Manganizithe, Stella; Malawi Epidemiology and Intervention Research Unit<br>Kumambala, Annie; Malawi Epidemiology and Intervention Research Unit<br>Sandford, Becky; Guy's and St Thomas' NHS Foundation Trust
Keywords:	EPIDEMIOLOGY, CARDIOLOGY, Cardiac Epidemiology < CARDIOLOGY, Hypertension < CARDIOLOGY

SCHOLARONE™
Manuscripts

I, the Submitting Author has the right to grant and does grant on behalf of all authors of the Work (as defined in the below author licence), an exclusive licence and/or a non-exclusive licence for contributions from authors who are: i) UK Crown employees; ii) where BMJ has agreed a CC-BY licence shall apply, and/or iii) in accordance with the terms applicable for US Federal Government officers or employees acting as part of their official duties; on a worldwide, perpetual, irrevocable, royalty-free basis to BMJ Publishing Group Ltd ("BMJ") its licensees and where the relevant Journal is co-owned by BMJ to the co-owners of the Journal, to publish the Work in this journal and any other BMJ products and to exploit all rights, as set out in our licence.

The Submitting Author accepts and understands that any supply made under these terms is made by BMJ to the Submitting Author unless you are acting as an employee on behalf of your employer or a postgraduate student of an affiliated institution which is paying any applicable article publishing charge ("APC") for Open Access articles. Where the Submitting Author wishes to make the Work available on an Open Access basis (and intends to pay the relevant APC), the terms of reuse of such Open Access shall be governed by a Creative Commons licence – details of these licences and which Creative Commons licence will apply to this Work are set out in our licence referred to above.

Other than as permitted in any relevant BMJ Author's Self Archiving Policies, I confirm this Work has not been accepted for publication elsewhere, is not being considered for publication elsewhere and does not duplicate material already published. I confirm all authors consent to publication of this Work and authorise the granting of this licence.

For peer review only

The prevalence and risk factors of lower extremity disease in high risk groups in Malawi: a stratified cross-sectional study

Author and author affiliations

Stephen Kasenda[1], Amelia C. Crampin[1,2,3], Justine Davies[4,5,6*], Jullita Kenala Malava[1], Stella Manganizithe[1], Annie Kumambala[1], Becky Sandford[7]

Author affiliations

[1] Malawi Epidemiology and Intervention Research Unit, Lilongwe, Malawi

[2] London School of Hygiene & Tropical Medicine, London, UK

[3] University of Glasgow, Institute of Health and Wellbeing, Glasgow, UK

[4] Institute of Applied Health Research, University of Birmingham, UK

[5] Centre for Global Surgery, Department of Global Health, Stellenbosch University, Cape Town

[6] Medical Research Council/Wits University Rural Public Health and Health Transitions Research Unit, Faculty of Health Sciences, School of Public Health, University of the Witwatersrand, Johannesburg, South Africa

[7] Guy's and St Thomas' NHS Trust, London, UK

*Corresponding author

Author contact details and ORCID

SK: Stephen.kasenda@meiru.mw**;** 0000-0001-9208-4122

AC: mia.crampin@lshtm.ac.uk; 0000-0002-1513-4330

JD: J.Davies.6@bham.ac.uk; 0000-0001-6834-1838

JM: jullita.malava@meiru.mw;

SM: stellamanganizithe@gmail.com

AM: anniekumambala@gmail.com

For peer review only

BS: Becky.Sandford@gstt.nhs.uk; 0000-0001-6337-936X

For peer review only

Abstract

Objective: Low and middle income countries face a disproportionate burden of cardiovascular diseases. However, amongst cardiovascular diseases, burden of and associations with lower extremity disease (peripheral arterial disease and/or diabetic foot disease) is neglected. We investigated the prevalence and factors associated with lower extremity disease (LED) among individuals known to have cardiovascular risk factors in Malawi, a low income country with a significant prevalence of cardiovascular disease risk factors.

Design: This was a stratified cross-sectional study.

Setting: This study was conducted in urban Lilongwe area 25, and the rural Karonga Health and Demographic Surveillance Site.

Participants: Participants were at least 18 years old and had been identified to have two or more known risk factors for cardiovascular disease (CVDRF).

Main outcome measures: Lower extremity disease—determined by the presence of one of the following: neuropathy (as assessed by a 10g monofilament), arterial disease (absent peripheral pulses, claudication as assessed by the Edinburgh claudication

For peer review only - http://bmjopen.bmj.com/site/about/guidelines.xhtml

questionnaire, or ankle brachial pulse index <0.9), previous amputation or ulceration of the lower limbs.

Results: There were 806 individuals enrolled into the study. Mean age was 52.5 years; 53.5% of participants were male (n=431) and 56.7% (n=457) were from the rural site. Nearly a quarter (24.1%; 95%: 21.2—27.2) of the participants had at least one symptom or sign of LED. 12.8% had neuropathy, 6.7% had absent pulses, 10.0% had claudication, 1.9% had ABPI<0.9, 0.9% had an amputation, and 1.1% had lower limb ulcers. LED had statistically significant association with increasing age, urban residence, and use of indoor fires.

Conclusions: This study demonstrated that a quarter of individuals with two or more CVDRFs have evidence of LED and 2.4% have an amputation or signs of limb threatening ulceration or amputation. Further epidemiological and health systems research is warranted to prevent LED and limb loss.

Strengths and limitations of this study

- Little is known about lower extremity disease in Malawi
- The use of multiple parameters to ensured more accurate estimation of LED prevalence
- calcification in vessels may result in false negative ABPI results

Data availability statement: Data are available on reasonable request

Word count: 3000

Supplemental material: For detailed data on the distribution

Keywords: Malawi, peripheral artery disease, cardiovascular diseases, HIV, hypertension, diabetes mellitus, obesity

Funding: This work was supported by King's Health Partners and Development Challenge Fund (MC_PC_16048). The baseline survey was funded by the Wellcome Trust (098610/Z/12/Z and 098610/B/12/A). The funders had no role in the study design;

For peer review only - http://bmjopen.bmj.com/site/about/guidelines.xhtml

collection, analysis, and interpretation of data; or report writing. The corresponding author had full access to the data and the final responsibility to submit for publication.

Competing interests: None declared.

Author contributions:

SK: data collection, analysis, drafting and revising manuscript,

AC: data collection, design, analysis and revising manuscript

JD: design, analysis and revising manuscript

SM: data collection and revising manuscript

AM: data collection and revising manuscript

BS: design, analysis and revising manuscript

For peer review only - http://bmjopen.bmj.com/site/about/guidelines.xhtml

Introduction

Peripheral vascular disease, peripheral neuropathies, and diabetic foot disease share a common group of overlapping risk factors, namely cardiovascular risk factors (CVDRFs) such as hypertension, diabetes, smoking, obesity, and hypercholesterolaemia. Infectious agents, particularly HIV also contribute to increased risk of peripheral vascular disease(1). Resources required to optimise preventative strategies, treat complications, and offer rehabilitation after surgery for peripheral vascular disease, peripheral neuropathies, and diabetic foot disease are largely the same whether the underlying disease process was predominantly ischaemic, infective, or neuropathic. As such, we have considered these conditions as one entity representing a spectrum of problems under the term 'Lower extremity disease" (LED). Individuals with LED are at risk of lower limb amputation, particularly in cases where there is significant ischaemic necrosis or infection. Amputation can be a life-saving procedure, relieving pain and sepsis, but may also be associated with negative physical, psychological, social, and economic consequences(2,3).

With increasing prevalence of CVDRF in lower and middle income countries (LMIC) superimposed on a background prevalence of chronic infections, it is strongly suspected that associated risk of lower limb amputation will become increasingly important for individuals, families, health systems, and economies(4). Evidence from LMICs is lacking, but suggestive that burden of amputations related to LED conditions will grow. Almost 70% of people with peripheral arterial disease live in LMICs; African countries in particular show a high population-prevalence of peripheral arterial disease (e.g. 33% in a population of people >65 years old in Central Africa)(5–7). Studies in people who have managed to

For peer review only - http://bmjopen.bmj.com/site/about/guidelines.xhtml

access CVDRF clinics in referral hospitals also show a large number of people have peripheral artery disease; for example, in Ghana, 27% of nearly 1000 people with diabetes had clinically measurable peripheral arterial disease(8). In our pilot study of 191 patients attending a hospital clinic in Blantyre, Malawi, peripheral arterial disease was seen in 8.5% of 45-64 year olds and 17% of people aged 65 and over(9). Reliable data on population prevalence of diabetic foot ulceration are lacking, however, studies show that in people with diabetes who have accessed services, prevalence ranged from 4.0 to 9.9%; it could be far higher if people who had not accessed services were included(10).

To develop health systems to manage and prevent LED and its consequences requires knowledge of its prevalence. The aim of this study was to investigate the population prevalence of LED amongst high risk individuals in rural and urban Malawi.

Methods

Study setting

The study was conducted in Area 25 in urban Lilongwe, and the rural Karonga Health and Demographic Surveillance Site (HDSS). The Karonga site has a population of approximately 45,000 and the economy is mainly subsistence farming. Area 25 (Lilongwe) has an approximate total population of 65,000 with a mixed economy.

Study design and participants

We conducted a stratified cross-sectional study of prevalence of “lower extremity disease” (LED) defined as neuropathy and/or vasculopathy in adults.

Identification of participants

Participants were at least 18 years old and had been identified to have two or more known risk factors for LED during a population wide survey of cardio-metabolic conditions conducted between 2013 and 2017 in the study areas(11). Presence of hypertension,

For peer review only - http://bmjopen.bmj.com/site/about/guidelines.xhtml

diabetes mellitus, HIV, tobacco smoking, obesity, and age at least 40 years were used to characterise high risk individuals.

Data collection and definitions

Data on participant socio-demographic characteristics (sex, date of birth, marital status, highest attained education, use of indoor fire, and household wealth) and presence of CVDRFs (history of smoking, diabetes mellitus, hypertension, dyslipidaemia, and HIV status using previous laboratory diagnosis) were extracted from the initial baseline study. Methods used to capture data and definitions used in that baseline survey have been published elsewhere(11). In brief, diabetes mellitus was defined as a fasting blood glucose of at least 7.0 mmol/L (as determined by a Beckman Coulter AU480 Chemistry Analyser) or self-report of a previous diagnosis of the condition by a health professional regardless of the drug history. Hypertension was defined as a blood pressure of at least 140/90 mmHg or currently on antihypertensive medication. Participants with a body mass index (BMI) of at least 30 kg/m^2 were classified as having obesity. Participants were defined as smokers if they reported smoking at least one cigarette per day in the immediate past six months preceding the survey. Participants were defined as HIV-positive if they had previously been tested antibody positive by the study team or self-reported HIV-positivity.

For the current study, participants were interviewed and examined in their own homes and data collectors were blind to the participant's previous medical history. In cases where the potential participant was missed during the first visit and it was established that they had neither left the study area nor died, the household was visited at least three times before declaring the potential participant as "missed". All participants were interviewed to determine if they had any changes in cardiovascular disease risk profile. The Edinburgh claudication questionnaire was used to determine the presence of claudication and participants who reported the presence of clinical CVDRFs were asked additional questions to regarding access to care for the relevant conditions they have.

For peer review only - http://bmjopen.bmj.com/site/about/guidelines.xhtml

Participants were examined to determine the presence of leg or foot ulcers, palpable peripheral pulses (brachial, posterior tibial, and dorsalis pedis), and/ or lower limb amputations bilaterally. Neuropen® 10g monofilaments were used to test for presence of peripheral neuropathy on the plantar surfaces great toe as well as over the metatarsal head of the great toe, 3rd and 5th toes of both feet. Portable sphygmomanometers (Welch Allyn DS55 Durashock hand aneroid model) and handheld continuous wave Doppler ultrasound devices (Bistos Hi.dop BT-200 vascular Doppler with 8 MHZ probe) were used to determine Ankle Brachial Pressure Index (ABPI). ABPI for each participant was determined according to the methods recommended by Aboyans et. al(12,13). A low ABPI reading suggests peripheral arterial disease, with the blood pressure reduced at the ankle compared with the arm which implies stenosis within the arterial tree supplying the lower limbs. Each participant was considered to have low ABPI if they had ABPI less than 0.9 on any or both sides.

Outcomes

The primary outcome of interest was the presence of any symptom or sign suggestive of lower extremity disease, termed "LED prevalence" (history of claudication, limb ulcers, loss of sensation on the plantar surfaces of feet, or Ankle Brachial Pressure Index [ABPI] less than 0.9). Secondary outcomes were these as individual outcomes.

Statistical analysis

Given the dearth of information on the prevalence of LED in the local setting, we based our sample size calculation on pilot data from a study of 90 people with diabetes living in the Malawi HDSS, which suggested that the prevalence of LED was around 10%. Considering people with diabetes to be at high risk and similar to those chosen for our study, we calculated a sample of 912 people would be required to detect a prevalence of LED of 10% with a CI of 6-14%.

For peer review only - http://bmjopen.bmj.com/site/about/guidelines.xhtml

Prevalence of the primary and secondary outcomes, socio-demographic characteristics of the study participants, and CVDRFs are described as mean (SD) and count (%). Logistic regression and likelihood ratio tests were done to determine associations between the primary outcome and independent variables. Socio-demographic characteristics and presence of CVDRFs were considered as a priori independent variables of interest in our analysis. The association between number of CVDRFs and the crude prevalence of the outcome of interest was also investigated.

Likelihood ratio testing was done to determine the presence of interactions between the independent variables. Stratified analyses of the full regression model were done for variables that were identified as having statistically significant interactions. All statistical analysis was done using Stata 15 statistical package.

Role of the funding source

Funding for this study was provided by King's Health Partners and Development Challenge Fund (MC_PC_16048), King's College London, UK. The baseline survey was funded by the Wellcome Trust (098610/Z/12/Z and 098610/B/12/A). The funders had no role in the study design; collection, analysis, and interpretation of data; or report writing. The corresponding author had full access to the data and the final responsibility to submit for publication.

Ethical approvals

Ethical approval for the baseline survey was granted by the Malawi National Health Sciences Research Committee (NHRSC) protocol number #1072, and London School of Hygiene and Tropical Medicine (LSHTM) Ethics Committee protocol number #6303. Ethical approval for the lower extremity disease study was granted by NHSRC approval number #2090 and King's College London BDM Research Ethics Panel (reference: LRS-17/18-7983). Data was collected from potential participants who gave written informed consent. Additional consent was sought from the participants for utilization of their previously obtained HIV results in data analysis for this study.

For peer review only - http://bmjopen.bmj.com/site/about/guidelines.xhtml

Patient and public involvement

There was no patient and public involvement in this study.

Results

A total of 829 people were visited, of whom 806 (97.2%) consented to be recruited (Figure 1). Most of the participants were at least 50 years age (mean age 52.5; 95%CI: 51.7—53.3), male (53.5%; n=431), from the rural site (56.7%; n=457), employed (70.6%; n=569), married (74.1%; n=597), and had no more than primary education (61.1%; n=493). Cooking with biomass fuel was reported by 94.8% (n=764) of the participants (Table 1). The majority 88.1% (n=710) of participants, had at least two clinical risk factors for LED (hypertension, obesity, HIV, diabetes, and dyslipidaemia), and the remaining 96 participants were selected based on having age greater than 40 years and a history of smoking tobacco (Figure 2 & Table 1). The mean age of the rural participants was higher than that of the urban participants (53.5 years [95%CI: 52.4—54.6] compared to 51.2 years [95%CI: 50.1—52.3]; P=0.005). Missing data accounted for not more than 6.1% of all the data on any variable.

Nearly a quarter (24.9%; 95%: 22.0—28.1) of the participants had at least one symptom or sign of LED (Table 2). Peripheral neuropathy (N=103; 12.8%) and claudication (N=81; 10 %) were the most common features of LED, and signs of limb threatening disease [i.e. ulcers (N=13; 1.1 %) and lower limb amputations (N=7; 0.9%)] were the least prevalent, affecting 2.4% (n=19) of participants. ABPI less than 0.9 was recorded among 32 (4.0%) participants, and 391 (48.5%) had ABPI greater than 1.2. (Appendix table 1) Four participants had ABPI less than 0.9 on one side & ABPI greater than 1.2 on the contralateral side. In view of the reduced ABPI <0.9, which is more likely to be a true positive, these individuals were categorised as having low ABPI. Those with ABPI >1.2 on both sides were grouped together with participants with ABPI between 0.9 and 1.2. At least 25% of participants with each CVDRF, apart from smoking, had LED. LED prevalence rates of over 30% were recorded among participants who were urban residents, female, aged over 50 years, single, indoor users of biomass fuel, or

For peer review only - http://bmjopen.bmj.com/site/about/guidelines.xhtml

homemakers. The prevalence of LED was directly proportional to the number of CVDRFs with the highest prevalence of LED [35.6%; 95%CI: 23.6—49.1] being recorded among participants that had five or more risk factors (table 2).

In the full regression model (figure 3), statistically significantly increased odds of LED were found in participants aged at least 50 years ((OR: 1.19; 95%CI: 1.22—2.99) among those aged 50-60, and OR: 2.33; 95%CI: 1.43—3.8 among those over 60 years age) and urban residents (OR: 1.76; 95%CI: 1.05—2.94) (Appendix table 2). Cooking with methods other than biomass burning was negatively associated with LED [OR: 0.19; 95%CI: 0.05—0.69]. The following sets of variables had some statistically significant interactions that affected the odds of the LED: (I) sex and history of smoking, (II) obesity and highest attained education, and (III) obesity and participant's wealth quintile, as computed in the full model. The results of these interactions are presented in Appendix tables 3- 6.

Discussion

The aim of this study was to describe the prevalence of lower extremity disease (LED) amongst individuals with known cardiovascular disease risk factors (CVDRF) in rural and urban Malawi. Of 806 individuals studied, a large proportion had at least one sign of LED, with claudication and peripheral neuropathy being the most common. Prevalence of LED was directly related to the number of risk factors with advancing age, urban living, and poverty demonstrating the greatest association. Although amputations and active ulceration were less frequently seen, 2.5% of individuals in this study had these conditions which are indicative of active or previous limb threatening disease.

Provision of health care services to manage cardiovascular risk factors or care for patients with LED are limited in Malawi and tend to be focussed around large urban centres(14). Health literacy also tends to be poor amongst those living with chronic conditions, with patients and families often understanding little about their condition or the longer term consequences of inadequate management(15). Provision of surgical services for the management of LED (e.g. vascular reconstructive techniques, amputation and

For peer review only - http://bmjopen.bmj.com/site/about/guidelines.xhtml

rehabilitation services) are limited throughout southern Africa and this field has not been viewed as a priority amongst competing healthcare development needs(16,17).

In poorly resourced health systems where people present late with disease, amputation rates and subsequent mortality is high. For example, a small study in Tanzania showed that 33% of people admitted to hospital with diabetic foot disease were managed by amputation and mortality was nearly 50%(18). By comparison, in the UK 17% of people presenting with disease underwent amputation within 1 year of presentation with no reported mortality(19). After limb-loss, poor post-operative care and the dearth of services for rehabilitation, prostheses, and mobility aids result in further mortality and morbidity(20). The devastating effects are tragic given there are simple and cheap strategies – including preventing and treating CVDRF and regular monitoring for signs of LED in high risk individuals with early referral to specialist services - to reduce deleterious outcomes.

Although this is one of the few studies to look at population prevalence of LED, (7)the findings of this study are in keeping with previously published work(8,21). For example, population prevalence of vascular disease has been reported at over 30% in those over 65 years(7).

An interesting 'U' shaped association between LED and wealth was observed, with the greatest prevalence of disease in the poorest and the wealthiest groups. The prevalence of hypertension and diabetes in these groups was also significantly higher at the extremes of wealth than in the middle 3 quintiles, which may go some way to explaining the observations. There may be other factors, beyond the scope of this study to characterise, such as lack of access to skilled healthcare services for optimum management of these comorbidities in the poorer communities(22), and potentially other social drivers towards worse ill-health such as sedentary lifestyle and westernised dietary habits amongst the wealthier group, which somewhat negate improved access to healthcare for those who are more wealthy(23–25). It will be important to fully understand these associations in future work in order to understand which interventions may be the most effective in preventing LED amongst different population groups.

For peer review only - http://bmjopen.bmj.com/site/about/guidelines.xhtml

Smoking status in this study was not shown to be a significant factor associated with LED, in contrast to a well-established body of evidence from high income countries(26). The reasons for this are not clear, however smoking is not as common in Malawi as in some high income countries and the cut off for being defined as a smoker in this study was more than 6 cigarettes per week, which is a relatively low level of consumption.

This was a large scale population based study, highlighting those at risk of limb loss amongst a group with known CVDRF. In focussing on population level data, the study reduces the potential for bias towards those who have already engaged with healthcare services. This is an advantage over much of the published literature to date, and provides a greater understanding of the scale of LED across different population groups in Malawi. The use of multiple approaches to evaluate the presence of LED is also a strength of this study, recognising that no single assessment is either sensitive or specific in isolation(4).

There are potential limitations to the conclusions to be drawn from this study. The use of ABPI and the Edinburgh Claudication Questionnaire as surrogate markers for peripheral vascular disease, whilst both validated techniques, do have drawbacks. Claudication, in itself, may never progress to represent a limb threatening condition, however can be a good marker of more generalised cardiovascular ill-health and therefore highlight opportunities for optimisation of best medical therapy in order to reduce cardiovascular mortality(27). ABPI may be confounded by calcification in vessels, resulting in falsely elevated ratios; this is particularly seen amongst diabetic individuals. The inclusion of those with ABPI>1.2 into the group with ABPI 0.9-1.2 may have resulted in some false negative results, neglecting to report arterial disease which was in fact present. Whilst toe brachial pressure index (TBPI) may be a more reliable observation in this group(28), there is little in the published data on TBPI studies in SSA and therefore interpretation of the findings from this study in the context of previously published work would have been more limited had this technique been utilised. Finally, we failed to achieve the sample size for this study, however the proportion of participants affected by LED was far greater than we had estimated and would have required a lower number of participants to reliably detect. In a retrospective calculation, 460 participants would have been required to detect

For peer review only - http://bmjopen.bmj.com/site/about/guidelines.xhtml

a prevalence of LED of 24% with the same width of confidence interval as in our initial power calculation.

In conclusion, this study demonstrated that 1 in 4 individuals with two or more cardiovascular risk factors have evidence of lower extremity disease and 2.5% already had active signs of limb threatening ulceration or previous amputation. An interesting U-shaped association with wealth was observed. Future work will need to explore the social determinants of health in relation to LED in order to guide health system interventions appropriately; the solutions may be complex and it is likely that there will not be a ‘one size fits all’ approach to preventing limb loss in this region.

For peer review only

For peer review only - http://bmjopen.bmj.com/site/about/guidelines.xhtml

References

1. Pillay B, Ramdial P, Naidoo D. HIV-associated large-vessel vasculopathy: a review of the current and emerging clinicopathological spectrum in vascular surgical practice: review article. Cardiovasc J Afr. 2015 Apr 30;26(2):70–81.

2. Madsen UR, Baath C, Berthelsen CB, Hommel A. Age and health-related quality of life, general self-efficacy, and functional level 12 months following dysvascular major lower limb amputation: a prospective longitudinal study. Disabil Rehabil. 2019 Nov 20;41(24):2900–9.

3. Taghipour H, Moharamzad Y, Mafi AR, Amini A, Naghizadeh MM, Soroush MR, et al. Quality of Life Among Veterans With War-Related Unilateral Lower Extremity Amputation: A Long-Term Survey in a Prosthesis Center in Iran: J Orthop Trauma. 2009 Aug;23(7):525–30.

4. Johnston LE, Stewart BT, Yangni-Angate H, Veller M, Upchurch GR, Gyedu A, et al. Peripheral Arterial Disease in Sub-Saharan Africa: A Review. JAMA Surg. 2016 Jun 1;151(6):564.

5. Fowkes FGR, Rudan D, Rudan I, Aboyans V, Denenberg JO, McDermott MM, et al. Comparison of global estimates of prevalence and risk factors for peripheral artery disease in 2000 and 2010: a systematic review and analysis. The Lancet. 2013 Oct;382(9901):1329–40.

6. Joshi R, Jan S, Wu Y, MacMahon S. Global Inequalities in Access to Cardiovascular Health Care. J Am Coll Cardiol. 2008 Dec;52(23):1817–25.

7. Guerchet M, Aboyans V, Mbelesso P, Mouanga AM, Salazar J, Bandzouzi B, et al. Epidemiology of Peripheral Artery Disease in Elder General Population of Two Cities of Central Africa: Bangui and Brazzaville. Eur J Vasc Endovasc Surg. 2012 Aug;44(2):164–9.

For peer review only

8. Yeboah K, Puplampu P, Ainuson J, Akpalu J, Gyan B, Amoah AGB. Peripheral artery disease and exertional leg symptoms in diabetes patients in Ghana. BMC Cardiovasc Disord. 2016 Dec;16(1):1–9.

9. Chisala P, Sandford B. Peripheral arterial disease among high-risk patients at a tertiary hospital in Blantyre, Malawi: A 2-phase observational study. East Cent Afr J Surg. 2020;25(3).

10. Okello S, Millard A, Owori R, Asiimwe SB, Siedner MJ, Rwebembera J, et al. Prevalence of lower extremity Peripheral artery disease among adult diabetes patients in Southwestern Uganda. BMC Cardiovasc Disord. 2014 Dec;14(1):75.

11. Price AJ, Crampin AC, Amberbir A, Kayuni-Chihana N, Musicha C, Tafatatha T, et al. Prevalence of obesity, hypertension, and diabetes, and cascade of care in sub-Saharan Africa: a cross-sectional, population-based study in rural and urban Malawi. Lancet Diabetes Endocrinol. 2018 Mar;6(3):208–22.

12. Aboyans V. The intra- and interobserver variability of ankle–arm blood pressure index according to its mode of calculation. J Clin Epidemiol. 2003 Mar;56(3):215–20.

13. Aboyans V, Criqui MH, Abraham P, Allison MA, Creager MA, Diehm C, et al. Measurement and Interpretation of the Ankle-Brachial Index: A Scientific Statement From the American Heart Association. Circulation. 2012 Dec 11;126(24):2890–909.

14. Chikowe I, Mwapasa V, Kengne AP. Analysis of rural health centres preparedness for the management of diabetic patients in Malawi. BMC Res Notes. 2018 Dec;11(1):267.

15. Assayed AA, Muula AS, Nyirenda MJ. The quality of care of diabetic patients in rural Malawi: A case of Mangochi district. Malawi Med J. 2014;6.

For peer review only

16. Veller MG. Education in vascular surgery–critical issues: A Southern African perspective. J Vasc Surg. 2008 Dec 1;48(6):84S-86S.

17. Magnusson L, Ahlström G, Ramstrand N, Fransson EI. Malawian prosthetic and orthotic users' mobility and satisfaction with their lower limb assistive device. J Rehabil Med. 2013 Apr;45(4):385–91.

18. Gulam-Abbas Z, Lutale JK, Morbach S, Archibald LK. Clinical outcome of diabetes patients hospitalized with foot ulcers, Dar es Salaam, Tanzania. Diabet Med. 2002 Jul;19(7):575–9.

19. Paisey RB, Abbott A, Levenson R, Harrington A, Browne D, Moore J, et al. Diabetes-related major lower limb amputation incidence is strongly related to diabetic foot service provision and improves with enhancement of services: peer review of the South-West of England. Diabet Med. 2018 Jan;35(1):53–62.

20. Guest JF, Fuller GW, Vowden P. Diabetic foot ulcer management in clinical practice in the UK: costs and outcomes. Int Wound J. 2018;15(1):43–52.

21. Zhang P, Lu J, Jing Y, Tang S, Zhu D, Bi Y. Global epidemiology of diabetic foot ulceration: a systematic review and meta-analysis. Ann Med. 2017 Feb 17;49(2):106–16.

22. Angwenyi V, Aantjes C, Kajumi M, De Man J, Criel B, Bunders-Aelen J. Patients experiences of self-management and strategies for dealing with chronic conditions in rural Malawi. Van Bogaert P, editor. PLOS ONE. 2018 Jul 2;13(7):e0199977.

23. Geldsetzer P, Manne-Goehler J, Marcus M-E, Ebert C, Zhumadilov Z, Wesseh CS, et al. The state of hypertension care in 44 low-income and middle-income countries: a cross-sectional study of nationally representative individual-level data from 1·1 million adults. The Lancet. 2019 Aug;394(10199):652–62.

For peer review only - http://bmjopen.bmj.com/site/about/guidelines.xhtml

24. Manne-Goehler J, Geldsetzer P, Agoudavi K, Andall-Brereton G, Aryal KK, Bicaba BW, et al. Health system performance for people with diabetes in 28 low- and middle-income countries: A cross-sectional study of nationally representative surveys. Wareham NJ, editor. PLOS Med. 2019 Mar 1;16(3):e1002751.

25. Kreatsoulas C, Frcpc SSA. The impact of social determinants on cardiovascular disease. 2010;26:6.

26. How Tobacco Smoke Causes Disease: The Biology and Behavioral Basis for Smoking-Attributable Disease. U.S. Department of Health and Human Services, Centers for Disease Control and Prevention, National Center for Chronic Disease Prevention and Health Promotion, Office on Smoking and Health; 2010.

27. Bevan GH, White Solaru KT. Evidence-Based Medical Management of Peripheral Artery Disease. Arterioscler Thromb Vasc Biol. 2020 Mar;40(3):541–53.

28. Williams DT, Harding KG, Price P. An Evaluation of the Efficacy of Methods Used in Screening for Lower-Limb Arterial Disease in Diabetes. Diabetes Care. 2005 Sep 1;28(9):2206–10.

For peer review only - http://bmjopen.bmj.com/site/about/guidelines.xhtml

Table 1: Summary of participant characteristics

Variables	N (%)	Both sites		Karonga (rural)		Lilongwe (urban)	
		men	women	men	women	men	women
TOTAL		431	375	303	154	128	221
Age							
Under 50	346(42.9)	199(46.2)	147(39.2)	138 (45.5)	51 (33.1)	61 (47.7)	96 (43.4)
50—60	239(29.7)	113(26.2)	126(33.6)	80 (26.4)	49 (31.8)	33 (25.8)	77 (34.8)
Over 60	221(27.4)	119(27.6)	102(27.2)	85 (28.1)	54 (35.1)	34 (26.6)	48 (21.7)
Wealth quintiles							
poorest	108 (13.4)	41 (9.5)	67 (17.9)	34 (11.2)	51 (33.1)	7 (5.5)	16 (7.2)
second	150 (18.6)	83 (19.3)	67 (17.9)	80 (26.4)	46 (29.9)	3 (2.3)	21 (9.5)
third	139 (17.3)	85 (19.7)	54 (14.4)	77 (25.4)	29 (18.8)	8 (6.3)	25 (11.3)
fourth	191 (23.7)	103 (23.9)	88 (23.5)	66 (21.8)	18 (11.7)	37 (28.9)	70 (31.7)
wealthiest	218 (27.1)	119 (27.6)	99 (26.4)	46 (15.2)	10 (6.5)	73 (57)	89 (40.3)
Marital status							
single	209 (25.9)	54 (12.5)	155 (41.3)	36 (11.9)	82 (53.3)	18 (14.1)	73 (33.0)
married	597 (74.1)	377 (87.5)	220 (58.7)	267 (88.1)	72 (46.8)	110 (85.9)	148 (67.0)
highest attained education							
0—5yrs primary education	164 (20.4)	59 (13.7)	105 (28)	48 (15.8)	66 (42.9)	11 (8.6)	39 (17.7)
Standard 6-8	329 (40.8)	184(42.7)	145 (38.7)	156 (51.5)	65 (42.2)	28 (21.9)	80 (36.2)
secondary	230 (28.5)	141 (32.7)	89 (23.7)	94 (31.0)	21 (13.6)	47 (36.7)	68 (30.8)
Post-secondary	83 (10.3)	47 (10.9)	36 (9.6)	5 (1.7)	2 (1.3)	42 (32.8)	34 (15.4)
Occupation							
homemaker	237 (29.4)	74 (17.2)	163 (43.5)	32 (10.6)	34 (22.1)	42 (32.8)	129 (58.4)

For peer review only - http://bmjopen.bmj.com/site/about/guidelines.xhtml

Farming/fishing	296 (36.7)	209 (48.5)	87 (23.2)	208 (68.7)	84 (54.6)	1 (0.8)	3 (1.36)
employed	273 (33.9)	148 (34.3)	125 (33.3)	63 (20.8)	36 (23.4)	85 (66.4)	89 (40.3)
**Indoor fire**							
no	42 (5.2)	26 (6.0)	16 (4.3)	17 (5.6)	6 (3.9)	9 (7.0)	10 (4.5)
Yes, inside the house	39 (4.8)	19 (4.4)	20 (5.3)	5 (1.7)	3 (2.0)	14 (10.9)	17 (7.7)
Yes, but in a separate kitchen	725 (90.0)	386 (89.6)	339 (90.4)	281 (92.7)	145 (94.2)	105 (82)	194 (87.8)
**Smoking history**							
Non- smokers	487 (60.4)	130 (30.2)	357 (95.2)	72 (23.8)	142 (92.2)	58 (45.3)	215 (97.3)
Smokers	319 (39.6)	301 (69.8)	18 (4.8)	231 (76.2)	12 (7.8)	70 (54.7)	6 (2.7)
**Diabetes**							
Non-diabetic	502 (62.3)	324 (75.2)	324 (75.2)	251 (82.8)	88 (57.1)	73 (57)	90 (40.7)
Diabetic	273 (33.9)	87 (20.2)	87 (20.2)	42 (13.9)	62 (40.3)	45 (35.2)	124 (56.1)
Missing data	31 (3.9)	20 (4.6)	20 (4.6)	10 (3.3)	4 (2.6)	10 (7.8)	7 (3.2)
**Dyslipidaemia**							
yes	344 (42.7)	238 (55.2)	175 (46.7)	177 (58.4)	73 (47.4)	61 (47.7)	102 (46.2)
no	413 (51.2)	165 (38.3)	179 (47.7)	114 (37.6)	74 (48.1)	51 (39.8)	105 (47.5)
Missing data	49 (6.1)	28 (6.5)	21 (5.6)	12 (4.0)	7 (4.6)	16 (12.5)	14 (6.3)
**Hypertension**							
hypertensive	482 (59.8)	220 (51.0)	104 (27.7)	171 (56.4)	40 (26.0)	49 (38.3)	64 (29.0)
Not hypertensive	324 (40.2)	211 (49.0)	271 (72.3)	132 (43.6)	114 (74.0)	79 (61.7)	157 (71.0)
**Mean blood pressure (SD)**							
	139.1 (38.2)	136(41.6)	142.6 (33.7)	137 (47)	149.1 (43.6)	133.7 (24.1)	138.1 (23.6)
**HIV**							

20

For peer review only - http://bmjopen.bmj.com/site/about/guidelines.xhtml

negative	413 (51.2)	237 (55.0)	176 (46.9)	170 (56.1)	80 (52.0)	67 (52.3)	96 (43.4)
positive	247 (30.7)	107 (24.8)	140 (37.3)	78 (25.7)	57 (37.0)	29 (22.7)	83 (37.6)
unknown	146 (18.1)	87 (20.2)	59 (15.7)	55 (18.2)	17 (11.0)	32 (25)	42 (19)
**Obesity**							
No	634 (78.7)	402 (93.3)	232 (61.9)	292 (96.4)	113 (73.4)	110 (85.9)	119 (53.9)
Yes	172 (21.3)	29 (6.7)	143 (38.1)	11 (3.6)	41 (26.6)	18 (14.1)	102 (46.2)

For peer review only

Table 2: prevalence of LED in total and by participant characteristics

Variables	N	LED prevalence (95% CI) n=194
**Total**	806	24.1 (21.2—27.2)
**Age**		
<50	346	15.9 (12.2—20.2)
50-60	239	27.6 (22.0—33.7)
>60	221	33.0 (26.9—39.7)
**Sex**		
Male	431	15.8 (12.5—19.6)
Female	375	33.6 (28.8— 38.6)
**Residence**		
Rural	457	17.5 (14.1—21.3)
Urban	349	32.7 (27.8—37.9)
**Wealth quintiles**		
poorest	108	32.4 (16.2—30.2)
second	150	22.7 (16.2—30.2)
third	139	15.8 (10.2—23.0)
fourth	191	24.1 (18.2—30.8)
wealthiest	218	26.1 (20.4—32.5)
**Marital status**		
Single	209	34.0 (27.6—40.8)
married	587	20.6 (17.4—24.1)
**Educational achievement**		
0-5 years primary education	164	25 (18.6—32.3)
Standard 6-8	329	25.5 (20.9—30.6)
Secondary	230	21.7 (16.6—27.6)
Post-secondary	83	22.9 (14.4—33.4)
**occupation**		
homemaker	237	37.1 (31.0—43.6)
Farming/ fishing	296	15.5 (11.6—20.2)
employed	273	22.0 (17.2—27.4)
**Indoor fire**		
none	42	7.1 (1.5—19.5)
Fire usually lit Inside the house	39	30.8 (17.0—47.6)

For peer review only - http://bmjopen.bmj.com/site/about/guidelines.xhtml

Fire usually lit in a separate kitchen area	725	24.7 (21.6—28.0)
**Any Smoking history**		
no	487	29.4 (25.4—33.6)
yes	319	16.0 (12.1—20.5)
**Diabetes**		
Non-diabetic	502	19.7 (16.3—23.5)
diabetic	273	33.7 (28.1—39.6)
Missing data	31	9.7 (2.0—25.8)
**Dyslipidaemia**		
no	413	22.8 (18.8—27.1)
yes	344	26.5 (21.9—31.5)
Missing data	49	18.4 (8.8—32.0)
**HIV**		
negative	413	23.0 (19.0—27.4)
positive	247	23.9 (18.7—29.7)
unknown	146	27.4 (20.3—35.4)
**hypertension**		
Not-hypertensive	324	21.9 (17.6—26.8)
hypertensive	482	25.5 (21.7—29.7)
**obesity**		
no	634	20.8 (17.7—24.2)
yes	172	36.0 (28.9—43.7)
**Number of risk factors**		
2	201	14.9 (10.3—20.6)
3	342	23.1 (18.7—27.9)
4	204	32.4 (26.0 —39.2)
≥5	59	32.2 (20.6—45.6)
**Prevalence of outcomes by risk factors**		
No diabetes and no hypertension	193	14.0 (25.6—42.4)
Diabetes but no hypertension	131	33.6 (25.6—42.4)
Hypertension but no diabetes	340	22.1 (17.8—26.8)
Hypertension and diabetes	142	33.8 (26.1—42.2)

For peer review only - http://bmjopen.bmj.com/site/about/guidelines.xhtml

For peer review only

Figure Legends

Figure 1: Flowcharts of study participation by site

Figure 2: three major risk factors and multi-morbidity across the study population

Figure 3: Coefficient plot of factors associated with lower extremity disease

For peer review only - http://bmjopen.bmj.com/site/about/guidelines.xhtml

FIGURE 1: Flowcharts of study participation by site

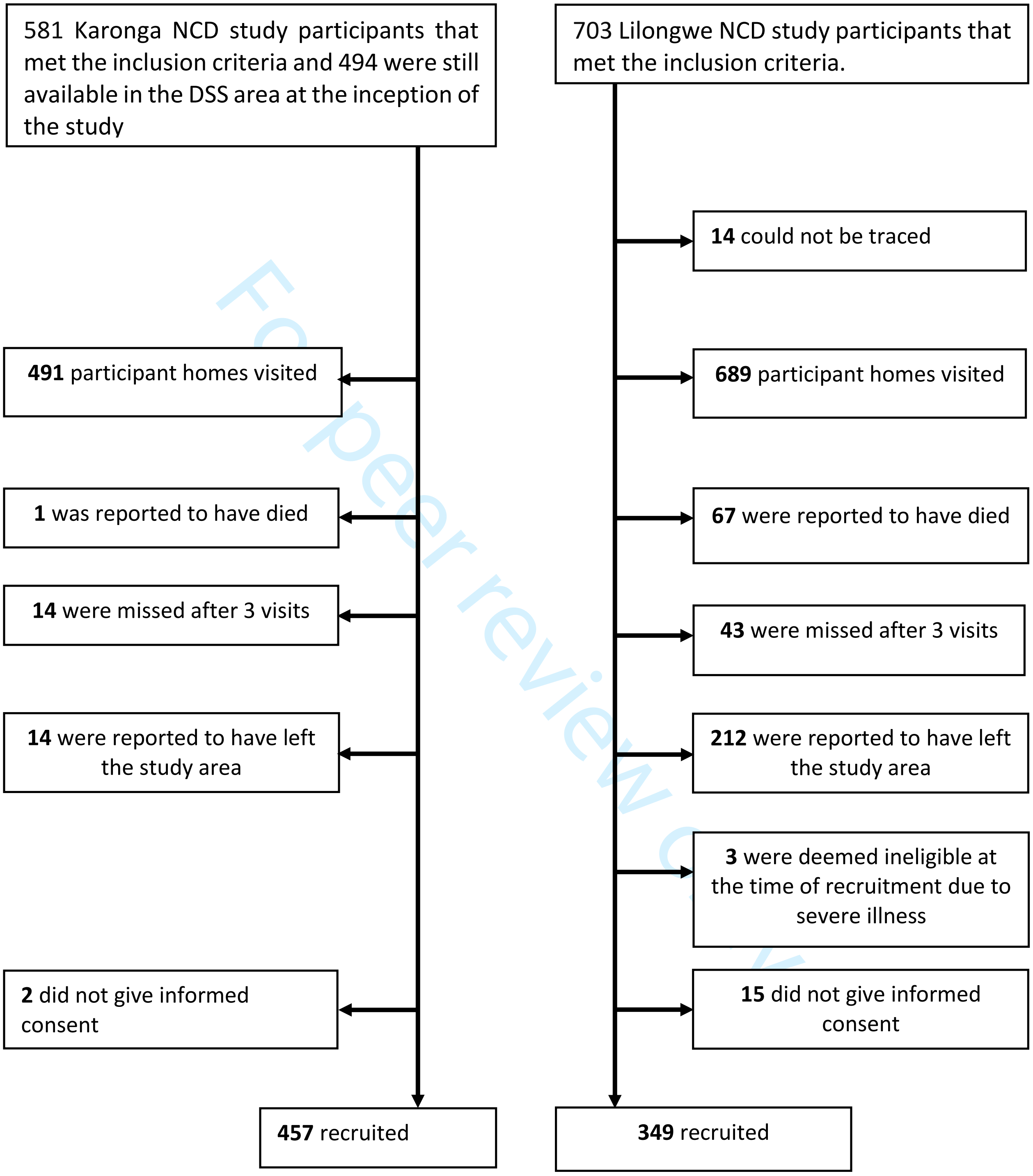

FIGURE 2: three major risk factors and multi-morbidity across the study population

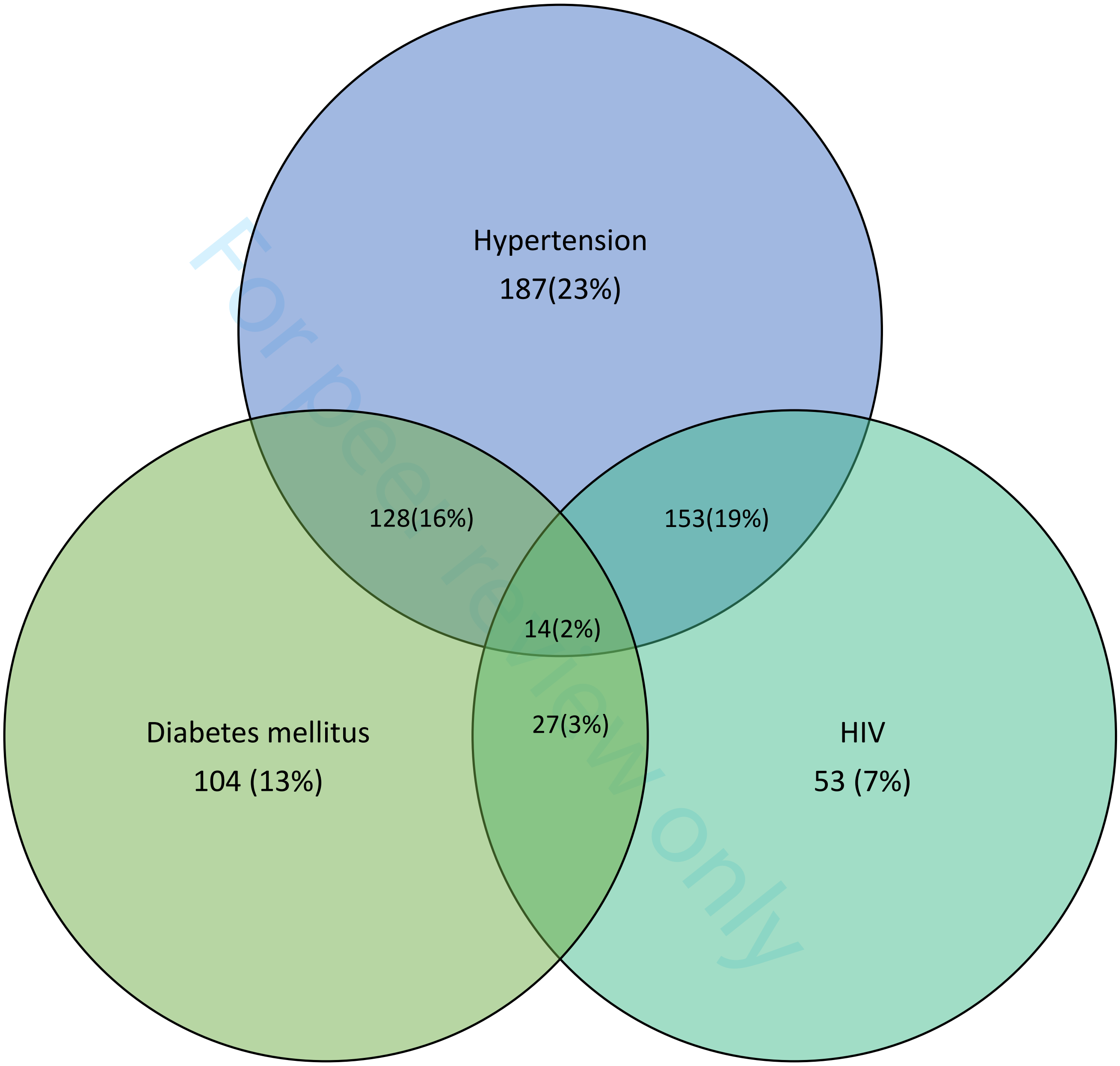

1

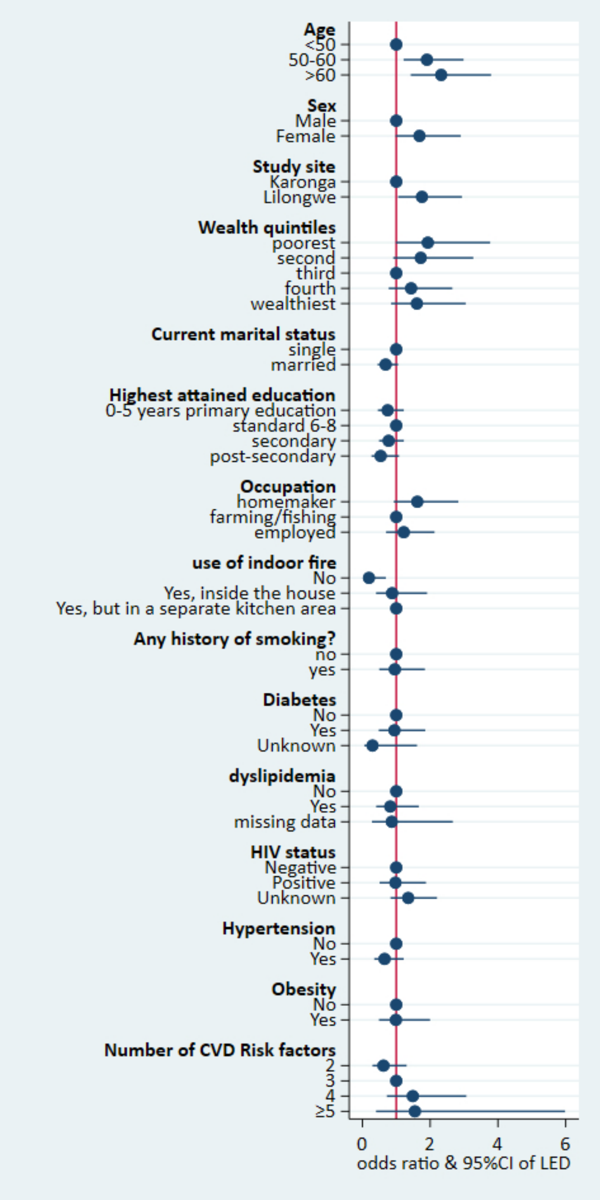

Coefficient plot of factors associated with lower extremity disease

125x250mm (96 x 96 DPI)

Appendix

Appendix table 1: Crude prevalence of factors associated with LED

		OUTCOME						
	N	LED prevalence (95% CI) n=194	Absent pulse prevalence (95% CI) n=54	Amputation Prevalence (95% CI) n=7	Ulcer Prevalence (95% CI) n=13	Claudication Prevalence (95% CI) n=81	Neuropathy Prevalence (95% CI) n=103	ABPI<0.9 Prevalence (95% CI) n=18
**Total**								
	806	24.1 (21.2—27.2)	6.7 (5.1—8.7)	0.9 (0.3—1.8)	1.1 (0.4— 1.8)	10 (8.1—12.3)	12.8 (10.6—15.3)	1.9 (1.0—3.1)
**Age**								
<50	346	15.9 (12.2—20.2)	4.9 (2.9—7.8)	0.3 (0.0—1.6)	2.3 (1.0—4.5)	9.0 (6.2—12.5)	6.1 (3.8—9.1)	0.3 (0.0—1.6)
50-60	239	27.6 (22.0—33.7)	6.7 (3.9—10.6)	2.1 (0.7—4.8)	0.8 (0.1—3.0)	12.6 (8.6—17.4)	15.1 (10.8—20.2)	0.8 (0.1—3.0)
>60	221	33.0 (26.9—39.7)	9.5 (6.0—14.2)	0.5 (0.0—2.5)	1.4 (0.3—3.9)	9.0 (5.6—13.6)	20.8 (15.7—26.8)	5.4 (2.8—9.3)
**Sex**								
Male	431	15.8 (12.5—19.6)	5.8 (3.8—8.4)	0.5 (0.1—1.7)	1.4 (0.5—3.0)	4.2 (2.5—6.5)	10.2 (7.5—13.5)	0.9 (0.3—2.4)
Female	375	33.6 (28.8— 38.6)	7.7 (5.2—10.9)	1.3 (0.4—3.1)	1.9 (0.8— 3.8)	16.8 (13.2—21.0)	15.7 (12.2—19.8)	2.9 (1.5—5.2)
**Residence**								
Rural	457	17.5 (14.1—21.3)	9.0 (6.5—12.0)	1.1 (0.4—2.5)	1.8 (0.8—3.4)	2.2 (1.1— 4.0)	13.8 (10.8—17.3)	1.8 (0.8—3.4)
Urban	349	32.7 (27.8—37.9)	3.7 (2.0—6.3)	0.6 (0.1— 2.1)	1.4 (0.5—3.3)	20.3 (16.2—25.0)	11.5 (8.3—15.3)	2.0 (0.8—4.1)
**Wealth quintiles**								
poorest	108	32.4 (16.2—30.2)	13.0 (7.3—20.8)	0.9 (0.02—5.1)	0.9 (0.02—5.1)	7.5 (2.6—12.9)	25 (17.2—34.3)	5.6 (2.1—11.7)

For peer review only

second	150	22.7 (16.2—30.2)	5.3 (2.3—10.2)	0.0 (0—2.4)	0.6 (0.02—3.7)	7.3 (3.7—12.7)	14.0 (8.9—20.6)	2.0 (0.4—5.7)
third	139	15.8 (10.2—23.0)	6.5 (3.0—11.9)	0.0 (0—2.6)	1.4 (0.2—5.1)	6.5 (3.0—11.9)	8.6 (4.5—14.6)	0.7 (0.0—3.9)
fourth	191	24.1 (18.2—30.8)	4.7 (2.2—8.8)	2.1 (0.6—5.3)	0.5 (0.0—2.9)	13.1 (8.7—18.7)	11.0 (6.9—16.3)	1.6 (0.3—4.5)
wealthiest	218	26.1 (20.4—32.5)	6.4 (3.6—10.5)	0.9 (0.1—3.3)	3.7 (1.6—7.1)	13.3 (9.1—18.5)	10.1 (6.4—15.9)	0.9 (0.1—3.3)
Marital status								
Single	209	34.0 (27.6—40.8)	10.0 (6.3—15.0)	1.0 (0.1—3.4)	1.9 (0.5—4.8)	12.4 (8.2—17.7)	18.7 (13.6—24.6)	3.8 (1.7—7.4)
married	587	20.6 (17.4—24.1)	5.5 (3.8—7.7)	0.8 (0.3—1.9)	1.5 (0.7—2.8)	9.2 (7.0—11.8)	10.7 (8.4—13.5)	1.2 (0.5—2.4)
Educational achievement								
0-5 years primary education	164	25 (18.6—32.3)	7.9 (4.3—13.2)	0 (0.0—2.2)	1.2 (0.1—4.3)	6.7 (3.4—11.7)	16.5 (11.1—23.0)	4.3 (1.7—8.6)
Standard 6-8	329	25.5 (20.9—30.6)	6.7 (4.2—9.9)	0.6 (0.1—2.2)	1.5 (0.5—3.5)	11.9 (8.6—15.8)	12.5 (9.1—16.5)	2.1 (0.9—4.3)
Secondary	230	21.7 (16.6—27.6)	6.1 (3.4—10.0)	1.7 (0.5—4.4)	2.6 (1.0—5.6)	8.2 (5.0—12.6)	12.2 (8.2—17.1)	0.4 (0.0—2.4)
Post-secondary	83	22.9 (14.4—33.4)	6.0 (2.0—13.5)	1.2 (0.0—6.5)	0.0 (0—4.3)	14.5 (7.7—23.9)	8.4 (3.5—16.6)	0.0 (0.0—4.3)
occupation								
homemaker	237	37.1 (31.0—43.6)	7.2 (4.2—11.2)	1.7 (0.5—4.3)	2.5 (0.9—5.4)	18.6 (13.8—24.1)	15.6 (11.2—20.9)	4.2 (2.0—7.6)
Farming/ fishing	296	15.5 (11.6—20.2)	9.1 (6.1—13.0)	0.3 (0.0—1.9)	1.0 (0.2—2.9)	1.7 (0.6—3.9)	12.8 (9.2—17.9)	1.7 (0.6—3.9)
employed	273	22.0 (17.2—27.4)	3.7 (1..8—6.6)	0.7 (0.0—2.6)	1.5 (0.4—3.7)	11.7 (8.2—16.1)	10.3 (6.9—14.5)	0.0 (0—1.3)
Indoor fire								
none	42	7.1 (1.5—19.5)	4.8 (0.6—16.2)	0.0 (0—8.4)	2.4 (0.0—12.6)	4.8 (0.6—16.2)	0.0 (0—8.4)	0.0 (0—8.4)
Fire usually lit Inside the house	39	30.8 (17.0—47.6)	7.7 (1.6—20.9)	0.0 (0—9.0)	2.6 (0.0—13.5)	17.9 (7.5—33.5)	12.8 (4.3—27.4)	0.0 (0—9.0)
Fire usually lit in a separate	725	24.7 (21.6—28.0)	6.8 (5.0—8.8)	1.0 (0.4—2.0)	1.5 (0.8—2.7)	9.9 (7.9—12.3)	13.5 (11.1—16.2)	2.1 (1.2—3.4)

For peer review only

kitchen area								
**Any Smoking history**								
no	487	29.4 (25.4—33.6)	7.6 (5.4—10.3)	1.2 (0.5—2.7)	1.8 (0.8—3.5)	14.0 (11.0—17.4)	14.4 (11.4—17.8)	2.1 (1.0—3.7)
yes	319	16.0 (12.1—20.5)	5.3 (3.1—8.4)	0.3 (0.0—1.7)	1.3 (0.3—3.2)	4.1 (2.2—6.9)	10.3 (7.2—14.2)	1.6 (0.5—3.6)
**Diabetes**								
Non-diabetic	502	19.7 (16.3—23.5)	6.2 (4.2—8.7)	0 (0—0.7)	1.2 (0.4—2.6)	8.2 (5.9—10.9)	10.2 (7.7—13.1)	1.4 (0.6—2.9)
diabetic	273	33.7 (28.1—39.6)	7.7 (4.8—11.5)	2.6 (1.0—5.2)	2.2 (0.8—4.7)	13.9 (10.0—18.6)	18.7 (14.2—23.8)	2.9 (1.3—5.7)
Missing data	31	9.7 (2.0—25.8)	6.5 (0.8—21.4)	0.0 (0—11.2)	3.2 (0.0–16.7)	6.5 (0.8—21.4)	3.2 (0.1—16.7)	0.0 (0.0—11.2)
**Dyslipidaemia**								
no	413	22.8 (18.8—27.1)	6.5 (4.4—9.4)	0.7 (0.2—2.1)	1.2 (0.4—2.8)	10.4 (7.6—13.8)	10.7 (7.8—14.0)	2.4 (1.2—4.4)
yes	344	26.5 (21.9—31.5)	7.0 (4.5—10.2)	1.2 (0.3—3.0)	1.7 (0.6—3.8)	10.2 (7.2—13.9)	15.7 (12.0—20.0)	1.5 (0.5—3.4)
Missing data	49	18.4 (8.8—32.0)	6.1 (1.3—16.9)	0.0 (0.0—7.3)	4.1 (0.5—14.0)	6.1 (1.3—16.9)	10.2 (3.4—22.2)	0.0 (0.0—7.3)
**HIV**								
negative	413	23.0 (19.0—27.4)	7.5 (5.2—10.5)	0.7 (0.2—2.1)	1.2 (0.4—2.8)	10.0 (7.2—13.2)	12.1 (9.1—15.6)	2.4 (1.2—4.4)
positive	247	23.9 (18.7—29.7)	6.1 (3.4—9.8)	0.4 (0.0—2.2)	2.0 (0.7—4.7)	10.1 (6.7—14.6)	12.6 (8.7—`17.3)	0.8 (0.1—2.9)
unknown	146	27.4 (20.3—35.4)	5.5 (2.4—10.5)	0.0 (0—2.5)	0.7 (0.0—3.8)	10.3 (5.9—16.4)	15.1 (9.7—21.9)	2.7 (0.8—6.9)
**hypertension**								
Not-hypertensive	324	21.9 (17.6—26.8)	4.6 (2.6—7.5)	0.9 (0.2—2.7)	2.2 (0.9—4.4)	9.3 (6.3—13.0)	12.7 (9.2—16.8)	0.6 (0.1—2.2)
hypertensive	482	25.5 (21.7—29.7)	8.1 (5.8—10.9)	0.8 (0.2—2.1)	1.2 (0.5—2.7)	10.6 (8.0—13.7)	12.9 (10.0—16.2)	2.7 (1.4—4.6)
**obesity**								

no	634	20.8 (17.7—24.2)	5.8 (4.1—8.0)	0.6 (0.2—1.6)	1.6 (0.8—2.9)	7.3 (5.4—9.6)	11.8 (9.4—14.6)	1.9 (1.0—3.3)
yes	172	36.0 (28.9—43.7)	9.9 (5.9—15.4)	1.7 (0.4—5.0)	1.7 (0.4—5.0)	20.3 (14.6—27.1)	16.3 (11.1—22.7)	1.7 (0.4—5.0)
Number of risk factors								
2	201	14.9 (10.3—20.6)	3.0 (1.1—6.4)	1.0 (0.1—3.5)	1.1 (0.1–3.5)	7.5 (4.2—12.0)	6.5 (3.4—10.8)	0.5 (0.0—2.7)
3	342	23.1 (18.7—27.9)	6.7 (4.3—9.9)	0.3 (0.0—1.6)	2.0 (0.8—4.2)	8.8 (6.0—12.3)	12.0 (8.7—15.9)	2.6 (1.2—4.9)
4	204	32.4 (26.0 —39.2)	9.3 (5.7—14.2)	0.5 (0.0—2.7)	1.0 (0.5—4.9)	14.7 (10.1—20.3)	18.1 (13.1—24.1)	2.0 (0.5—4.9)
≥5	59	32.2 (20.6—45.6)	10.2 (3.8—20.8)	5.1 (1.1—14.1)	0.0 (0.0—6.1)	10.2 (3.8—20.8)	20.3 (11.0—32.8)	1.7 (0.0—9.1)
Prevalence of outcomes by risk factors								
No diabetes and no hypertension	193	14.0 (25.6—42.4)	4.1 (1.8—8.0)	0.0 (0.0—1.9)	1.6 (0.3—4.4)	4.7 (2.2—8.7)	9.3 (5.6—14.3)	0.0 (0.0—1.9)
Diabetes but no hypertension	131	33.6 (25.6—42.4)	5.3 (2.2—10.7)	2.3 (0.5 –6.5)	3.1 (0.8—7.6)	16.0 (10.2—23.5)	17.6 (11.5—25.2)	1.5 (0.2—5.4)
Hypertension but no diabetes	340	22.1 (17.8—26.8)	7.4 (4.8—10.7)	0.0 (0.0 —1.1)	1.2 (0.3—3.0)	10 (7.0—13.7)	10.0 (7.0—13.7)	2.1 (0.8—4.2)
Hypertension and diabetes	142	33.8 (26.1—42.2)	9.9 (5.5—16.0)	2.8 (0.8 —7.1)	1.4 (0.2 —5.0)	12.0 (7.1—18.5)	19.7 (13.5—27.2)	4.2 (1.6—9.0)

For peer review only

Appendix table 2: full regression model of factors associated with Lower extremity disease

	Odds Ratio	Std. Err.	z	P>z	95% confidence interval	Lr test p value
**Age**						
<50	1	ref.				0.0012
50-60	1.91	0.4	2.8	0.0	1.22—2.99	
>60	2.33	0.6	3.4	0.0	1.43—3.8	
**sex**						
Male	1.0	ref.				0.0574
Female	1.69	0.5	1.9	0.1	0.98—2.9	
**site**						
Karonga	1.0	ref.				0.0296
Lilongwe	1.76	0.5	2.2	0.0	1.05—2.94	
**Wealth quintiles**						
poorest	1.93	0.7	1.9	0.1	0.99—3.77	0.3322
second	1.72	0.6	1.7	0.1	0.91—3.27	
third	1.0	ref.				
fourth	1.43	0.4	1.2	0.2	0.78—2.66	
wealthiest	1.61	0.5	1.5	0.1	0.85—3.05	
**Marital status**						
single	1.0	ref.				0.0862
married	0.69	0.1	-1.7	0.1	0.45—1.05	
**Highest attained education**						
years primary education	0.75	0.2	-1.2	0.2	0.46—1.22	0.2630
standard 6-8	1.0	ref.				
secondary	0.78	0.2	-1.1	0.3	0.49—1.22	
post-secondary	0.54	0.2	-1.7	0.1	0.27—1.08	
**Occupation**						
homemaker	1.62	0.5	1.7	0.1	0.93—2.83	0.2026
farming/fishing	1.0	ref.				
employed	1.22	0.3	0.7	0.5	0.7—2.13	
**Indoor smoke**						
No	0.19	0.1	-2.5	0.0	0.05—0.69	0.0105
Yes, inside the house	0.88	0.3	-0.3	0.7	0.4—1.91	
Yes, in a separate kitchen area	1.0	ref.				
**any history of smoking**						
no	1.0	ref.				0.8995
yes	0.96	0.3	-0.1	0.9	0.5—1.84	
**Diabetes**						
No	1.0	ref.				0.3532
Yes	0.95	0.3	-0.2	0.9	0.48—1.86	

For peer review only

Unknown	0.3	0.3	-1.4	0.2	0.06—1.61	
**Dyslipidaemia**						
No	1.0	ref.				0.8425
Yes	0.82	0.3	-0.5	0.6	0.41—1.66	
missing data	0.87	0.5	-0.3	0.8	0.28—2.67	
**HIV status**						
Negative	1.0	ref.				0.4490
Positive	0.98	0.3	-0.1	0.9	0.51—1.88	
Unknown	1.35	0.3	1.2	0.2	0.83—2.2	
**hypertension**						
No	1.0	ref.				0.1768
Yes	0.65	0.2	-1.3	0.2	0.35—1.22	
**obesity**						
No	1.0	ref.				0.9754
Yes	0.99	0.4	0.0	1.0	0.49—2.0	
**CVDRF**						
2	0.62	0.2	-1.3	0.2	0.3—1.3	0.4812
3	1.0	ref.				
4	1.49	0.5	1.1	0.3	0.72—3.07	
≥5	1.54	1.1	0.6	0.5	0.4—5.98	

For peer review only

Stratified analysis for lower extremity disease

Appendix table 3: stratification by sex

	Male	Female
**Age**		
<50	(ref)	(ref)
50-60	1.96 (0.91—4.22)	1.97 (1.08—3.6)
>60	2.41 (1.08—5.37)	2 (1.0*—4.01)
**site**		
Karonga	(ref)	(ref)
Lilongwe	1.84 (0.79—4.31)	1.65 (0.82—3.34)
**Wealth quintiles**		
poorest	1.92 (0.6—6.19)	2.04 (0.86—4.88)
second	2.32 (0.87—6.18)	1.4 (0.58—3.39)
third	(ref)	(ref)
fourth	1.74 (0.65—4.64)	1.51 (0.66—3.46)
wealthiest	1.23 (0.42—3.6)	2.03 (0.87—4.72)
**Marital status**		
single	(ref)	(ref)
married	0.39 (0.18—0.86)	0.81 (0.47—1.39)
**Highest attained education**		
0-5 years prima..	1.01 (0.41—2.48)	0.69 (0.36—1.3)
standard 6-8	(ref)	(ref)
secondary	0.86 (0.42—1.73)	0.68 (0.36—1.28)
post-secondary	0.62 (0.19—2.06)	0.43 (0.17—1.09)
**Occupation**		
homemaker	1.09 (0.44—2.72)	2.19 (0.98—4.89)
farming/fishing	(ref)	(ref)
employed	0.91 (0.4—2.06)	1.87 (0.8—4.38)
**Indoor fire**		
No	(empty)	0.28 (0.07—1.17)
Yes, inside the house	0.66 (0.17—2.59)	1.06 (0.39—2.92)
Yes, but in a separate kitchen area.	(ref)	(ref)
**History of smoking**		
no	(ref)	(ref)
yes	0.79 (0.34—1.8)	1.83 (0.52—6.47)
**Diabetes**		
No	(ref)	(ref)
Yes	1.89 (0.71—5.01)	0.38 (0.13—1.18)
Unknown	0.48 (0.05—5.04)	0.18 (0.01—2.69)
**Dyslipidaemia**		

* Rounded up from .9989416

For peer review

No	(ref)	(ref)
Yes	0.91 (0.32—2.59)	0.48 (0.16—1.49)
missing data	1.05 (0.18—6.21)	0.6 (0.12—2.97)
HIV status		
Negative	(ref)	(ref)
Positive	1.81 (0.74—4.41)	0.38 (0.13—1.13)
Unknown	1.69 (0.8—3.58)	0.93 (0.46—1.85)
hypertension		
No	(ref)	(ref)
Yes	0.52 (0.22—1.24)	0.39 (0.13—1.19)
obesity		
No	(ref)	(ref)
Yes	2.13 (0.64—7.07)	0.5 (0.17—1.48)
CVDRF		
2	0.68 (0.23—1.97)	0.45 (0.14—1.47)
3	(ref)	(ref)
4	1.37 (0.48—3.94)	2.59 (0.82—8.2)
≥5	0.76 (0.09—6.61)	6.48 (0.73—57.42)

For peer review only

Appendix table 4: Stratification by Sex and history of smoking

Variables and variable categories	Male non-smoker	Male smoker	Female non-smoker	Female current smokers[†]
Age				
<50	(ref)	(ref)	(ref)	
50-60	2.74 (0.5—14.91)	1.43 (0.53—3.85)	2.1 (1.15—3.85)	
>60	4.53 (0.62—32.88)	1.86 (0.68—5.06)	1.95 (0.96—3.94)	
Site				
Karonga	(ref)	(ref)	(ref)	
Lilongwe	0.63 (0.12—3.41)	3.38 (1.07—10.62)	1.82 (0.88—3.78)	
Wealth Quintile				
poorest	6.26 (0.26—150.93)	1.58 (0.35—7.09)	1.71 (0.7—4.18)	
second	4.03 (0.18—89)	2.85 (0.9—9.05)	1.38 (0.56—3.43)	
third	(ref)	(ref)	(ref)	
fourth	4.67 (0.28—76.66)	1.27 (0.41—3.99)	1.24 (0.54—2.87)	
wealthiest	14.33 (0.91—225.09)	0.72 (0.16—3.21)	1.62 (0.69—3.81)	
Marital status				
single	(ref)	(ref)	(ref)	
married	0.04 (0.0—0.56)	0.58 (0.21—1.58)	0.91 (0.52—1.58)	
Education				
years primary education	4.93 (0.17—139.91)	0.88 (0.33—2.35)	0.64 (0.33—1.24)	
standard 6-8	(ref)	(ref)	(ref)	
secondary	3.02 (0.57—15.97)	0.62 (0.24—1.57)	0.78 (0.41—1.49)	
post-secondary	1.35 (0.19—9.71)	0.75 (0.07—8.19)	0.47 (0.19—1.18)	
Occupation				
homemaker	1.82 (0.29—11.26)	0.72 (0.2—2.6)	2.09 (0.9—4.88)	
farming/fishing	(ref)	(ref)	(ref)	
employed	0.43 (0.07—2.8)	1.1 (0.38—3.23)	1.68 (0.69—4.08)	
Indoor smoke				
No	(empty)	(empty)	(empty)	
Yes, inside the house	(empty)	0.82 (0.17—3.98)	1.02 (0.37—2.8)	
a separate kitchen area	(ref)	(ref)	(ref)	
diabetes				
No	(ref)	(ref)	(ref)	
Yes	1.57 (0.11—22.08)	3.79 (0.94—15.32)	0.35 (0.1—1.22)	
Unknown	(empty)	0.58 (0.02—16.66)	0.24 (0.02—3.85)	
Dyslipidaemia				
No	(ref)	(ref)	(ref)	
Yes	0.17 (0.01—3.9)	0.79 (0.2—3.14)	0.45 (0.13—1.53)	
missing data	0.28 (0.01—5.91)	0.7 (0.03—14.21)	0.57 (0.1—3.22)	
HIV status				

[†] No observations

Negative	(ref)	(ref)	(ref)	
Positive	1.82 (0.12—28.15)	2 (0.65—6.15)	0.41 (0.13—1.33)	
Unknown	2.01 (0.41—9.92)	1.74 (0.65—4.71)	0.99 (0.48—2.02)	
hypertension				
No	(ref)	(ref)	(ref)	
Yes	0.65 (0.06—6.93)	0.35 (0.1—1.15)	0.37 (0.11—1.25)	
obesity				
No	(ref)	(ref)	(ref)	
Yes	0.59 (0.04—9.2)	6.62 (1.06—41.46)	0.52 (0.16—1.72)	
CVDRF				
2	0.66 (0.04—11.7)	0.46 (0.11—1.85)	0.41 (0.11—1.49)	
3	(ref)	(ref)	(ref)	
4	20.29 (0.76—539.61)	0.51 (0.11—2.41)	2.67 (0.76—9.37)	
5	62.07 (0.17—22604.06)	0.26 (0.01—7.99)	6.71 (0.61—73.19)	

For peer review only

Appendix table 5: Stratification by obesity and wealth quintiles

Variables and variable categories	non obese WQ1	Non obese wq2	Non obese wq3	Non obese WQ4	Non obese WQ5	Obese WQ1[‡]	Obese WQ2[§]	Obese WQ3	Obese WQ4	Obese WQ5
**Age**										
<50	(ref)	(ref)	(ref)	(ref)	(ref)			1.45E-31 (.—.)	(ref)	(ref)
50-60	43.49 (1.51—1248.98)	4.57 (1.07—19.58)	0.8 (0.11—6.03)	2.63 (0.77—8.93)	0.85 (0.23—3.06)			5.54E-47 (.—.)	1.44 (0.07—30.58)	3.48 (0.5—24.14)
>60	12.11 (0.89—164.53)	4.43 (1.03—19.09)	0.42 (0.04—4.69)	3.82 (1.03—14.23)	2.18 (0.49—9.74)				1.91 (0.06—57.17)	2.65 (0.33—21.42)
**sex**										
Male	(ref)	(ref)	(ref)	(ref)	(ref)			(ref)	(empty)	(ref)
Female	3.29 (0.44—24.85)	4.32 (0.64—28.94)	0.62 (0.03—13.88)	1.26 (0.32—4.93)	1.67 (0.45—6.18)			8.6E+107 (.—.)	(omitted)	1.9 (0.19—18.76)
**Site**										
Karonga	(ref)	(ref)	(ref)	(ref)	(ref)			(ref)	(empty)	(ref)
Lilongwe	12.72 (0.89—182.11)	3.95 (0.65—24.04)	2.64 (0.35—19.71)	2.49 (0.73—8.5)	0.53 (0.11—2.43)			2.6E+15 (.—.)	(omitted)	5.05 (0.13—202.03)
**Marital status**										
single	(ref)	(ref)	(ref)	(ref)	(ref)			(ref)	(ref)	(ref)
married	1.39 (0.29—6.64)	0.95 (0.28—3.28)	0.29 (0.04—2.15)	0.5 (0.14—1.7)	0.64 (0.14—2.9)			8.6E+107 (.—.)	0.53 (0.03—8.15)	0.14 (0.02—1.04)
**Highest attained education**										
0—5 years primary education	11.09 (1.5—82.05)	0.89 (0.28—2.81)	0.34 (0.02—4.67)	0.76 (0.15—3.75)	2.09 (0.21—21.31)			8.1E-78 (.—.)	0.26 (0.0—16.1)	0.3 (0.02—5.47)
standard 6-8	(ref)	(ref)	(ref)	(ref)	(ref)			(ref)	(ref)	(ref)
secondary	57.88 (1.61—2080.34)	1.4 (0.3—6.59)	1.42 (0.27—7.35)	0.71 (0.24—2.11)	2.24 (0.63—7.94)			(empty)	0.34 (0.01—8.42)	0.49 (0.07—3.34)
post-secondary	42.93 (0.52—3528.5)	(empty)	(empty)	1.37 (0.08—24.48)	1.35 (0.27—6.7)				(empty)	0.37 (0.06—2.51)

[‡] No observations
[§] No observations

11

For peer review only

**occupation**										
homemaker	0.8 (0.11—5.7)	0.61 (0.12—3.13)	21.78 (0.94—504.37)	0.79 (0.15—4.1)	5.97 (0.83—42.82)			4.7E+61 (.—.)	2.00 (0.04—100.23)	0.73 (0.0—155.92)
farming/fishing	(ref)	(ref)	(ref)	(ref)	(ref)			(ref)	(empty)	(ref)
employed	2.15 (0.29—16.04)	1.43 (0.23—9.04)	13.15 (0.9—192.03)	0.66 (0.17—2.58)	1.33 (0.23—7.83)			1.5E+154 (.—.)	(omitted)	0.19 (0.0—34.71)
**Indoor smoke**										
No	(empty)	0.26 (0.02—3.55)	(empty)	(empty)	(empty)				(empty)	0.75 (0.04—15.21)
Yes, inside the house	(empty)	2.91 (0.27—30.92)	(empty)	3.89 (0.65—23.19)	0.12 (0.01—1.5)				(empty)	1.64 (0.18—15.18)
Yes, in a separate kitchen area	(ref)	(ref)	(ref)	(ref)	(ref)			(ref)	(ref)	(ref)
**History of smoke**										
no	(ref)	(ref)	(ref)	(ref)	(ref)			(ref)	(ref)	(ref)
yes	0.29 (0.0—17.25)	3.07 (0.33—28.35)	0.12 (0.0—4.39)	0.94 (0.17—5.11)	0.26 (0.04—1.65)			4.1E+169 (.—.)	(empty)	0.54 (0.01—23.36)
**Diabetes**										
No	(ref)	(ref)	(ref)	(ref)	(ref)			(ref)	(ref)	(ref)
Yes	0.13 (0—16.29)	2.43 (0.29—20.13)	0.17 (0.0—9.5)	2.54 (0.54—12)	1.15 (0.19—7.16)			1.8E+46 (.—.)	0.07 (0.0—14.48)	1.15 (0.09—14.87)
Unknown	(empty)	(empty)	2.1E-08 (0.1—22.1)	7.2E+05 (0.0—.)	(empty)				(empty)	
**Dyslipidaemia**										
No	(ref)	(ref)	(ref)	(ref)	(ref)			(ref)	(ref)	(ref)
Yes	0.06 (0.0—4.64)	0.58 (0.07—4.84)	0.06 (0.0—1.92)	3.03 (0.53—17.14)	0.06 (0.0—1.19)			2.6E+07 (.—.)	0.31 (0.0—22.6)	0.93 (0.03—26.3)
missing data	(empty)	(empty)	7.7E+07 (0.0—.)	2.0E-06 (0.0—.)	0.18 (0.02—2.11)			(empty)	(empty)	0.48 (0—64.61)
**HIV status**										
Negative	(ref)	(ref)	(ref)	(ref)	(ref)			(ref)	(ref)	(ref)
Positive	0.08 (0.0—6.74)	1.15 (0.17—7.63)	0.12 (0.0—4.26)	1.94 (0.37—10.03)	1.41 (0.24—8.31)			1.45E-31 (.—.)	0.24 (0—22.56)	1.98 (0.14—27.98)
Unknown	2.1 (0.33—13.38)	1.66 (0.4—6.81)	1.33 (0.08—22.1)	2.04 (0.5—8.3)	1.63 (0.27—9.77)			1.45E-31 (.—.)	0.39 (0.02—7.7)	1.79 (0.23—13.66)

For peer review only - http://bmjopen.bmj.com/site/about/guidelines.xhtml

Hypertension										
No	(ref)	(ref)	(ref)	(ref)	(ref)			(ref)	(ref)	(ref)
Yes	0.11 (0.0—9.9)	0.7 (0.1—4.87)	0.03 (0.0—0.91)	0.85 (0.21—3.47)	0.6 (0.09—4.13)			1.81E+46 (.—.)	0.33 (0.01—19.43)	1.41 (0.14—14.27)
CVDRF										
2	0.03 (0.0—1.88)	0.8 (0.1—6.22)	0.0 (0.0—0.29)	1.59 (0.28—9.08)	1.29 (0.17—9.96)					
3	(ref)	(ref)	(ref)	(ref)	(ref)			(ref)	(ref)	(ref)
4	49.06 (0.47—5153.28)	1.87 (0.18—18.98)	2.7 (0.06—112.27)	0.92 (0.18—4.78)	16.97 (1.14—253.16)			1.81E+46 (.—.)	1.17 (0.0—154.19	0.58 (0.02—18.29)
5	(empty)	(empty)	(empty)	(empty)	(empty)			(omitted)	6.75 (0.0—24323.16)	0.48 (0.0—105.76)

For peer review only

Appendix table 6: Stratification by highest attained education and obesity

Variables and variable categories	Non-obese & 0-5 years elementary education	Non-obese & 6-8 elementary education	Non obese & secondary education	Non-obese & post-secondary education[**]	Obese & 0-5 years elementary education[††]	Obese & 6-8 years elementary education	Obese & secondary education	Obese & post-secondary education[‡‡§§]
**Age**								
<50	(ref)	(ref)	(ref)	(ref)	(ref)	(ref)	(ref)	(ref)
50-60	10.76 (1.45—79.81)	1.33 (0.57—3.15)	2.18 (0.66—7.22)	.	2.07e+19 (.—.)	2.96 (0.6—14.49)	12.52 (0.3—525.67)	1.14E-57 (.—.)
>60	5.85 (0.9—38)	1.65 (0.63—4.29)	6.74 (1.83—24.92)	.	264.2 (.—.)	3.12 (0.43—22.52)	5.03 (0.02—1686.17)	1.64E+56 (.—.)
**Sex**								
Male	(ref)	(ref)	(ref)	(ref)	(omitted)	(ref)	(ref)	(ref)
Female	7.38 (0.9—60.6)	2.73 (0.77—9.71)	1.28 (0.35—4.69)	.		0.47 (0.03—8.69)	0.84 (0.02—45.4)	4.96E+56 (.—.)
**Site**								
Karonga	(ref)	(ref)	(ref)	(empty)	(ref)	(ref)	(ref)	(ref)
Lilongwe	5.38 (0.91—31.62)	3.06 (1.12—8.35)	0.88 (0.26—3.04)	(omitted)	3.6E+22 (.—.)	2.01 (0.23—17.74)	1.06 (0.01—97.43)	3.8E+107 (.—.)
**Wealth quintiles**								
poorest	25.05 (1.9—330.22)	1.59 (0.42—6.0)	1.72 (0.23—12.74)	.	1.8E+21 (.—.)	0.3 (0.03—3.47)	(empty)	(empty)
second	7.82 (0.71—86.4)	3.3 (1.02—10.68)	1.56 (0.28—8.67)		3.1E+42 (.—.)	0.37 (0.03—4.01)	13.09 (0.03—5949.07)	(empty)
third	(ref)	(ref)	(ref)	(empty)	(ref)	(ref)	(ref)	(empty)
fourth	2.4 (0.15—37.21)	1.85 (0.64—5.37)	1.96 (0.49—7.89)	(empty)	3.9E-21 (.—.)	0.22 (0.02—2.3)	0.24 (0—38.03)	(empty)
wealthiest	2.09 (0.1—42.23)	0.84 (0.24—2.92)	3.39 (0.75—15.3)	(omitted)	7.8 (.—.)	0.44 (0.04—4.71)	11.69 (0.09—1444.24)	(omitted)
**Marital status**								

[**] Site, wealth quintiles and occupation omitted because of collinearity
[††] Sex, occupation and history of smoking omitted because of collinearity
[‡‡] Wealth and occupation have no observations
[§§] History of smoking and CVDRF omitted because of collinearity

For peer review only

single	(ref)	(ref)	(ref)	(ref)	(ref)	(ref)	(ref)	(ref)
married	1.11 (0.28—4.43)	1.32 (0.54—3.23)	0.31 (0.11—0.91)	**0.0 (.—.)**	4.6E-22 (.—.)	0.69 (0.11—4.38)	0.13 (0.01—2.61)	7.38E+17
**occupation**								
homemaker	2.51 (0.56—11.17)	1.66 (0.54—5.08)	0.69 (0.17—2.84)	**0.0 (.—.)**	4.0 e+19 (.—.)	2.78 (0.29—26.69)	0.3 (0.01—12.69)	1.04E+95 (.—.)
farming/fishing	(ref)	(ref)	(ref)	(empty)	(empty)	(ref)	(empty)	(empty)
employed	1.87 (0.27—12.76)	1.83 (0.67—4.99)	0.5 (0.12—2.03)	**1 (omitted)**	1 **(omitted)**	1.71 (0.15—19.93)	1 (omitted)	(omitted)
**Indoor smoke**								
No	0.96 (0.04—21.49)	(Empty)	(Empty)	(empty)	(empty)	(empty)	(empty)	4.62E+74 (.—.)
Yes, inside the house	1.93 (0.21—17.91)	1.88 (0.42—8.4)	0.11 (0.01—1.59)	(empty)		0.93 (0.05—18.44)	5.99 (0.04—988.69)	4.72E+94 (.—.)
Yes, in a separate kitchen area	(ref)	(ref)	(ref)	(ref)	(ref)	(ref)	(ref)	(ref)
**Smoking history**								
no	(ref)	(ref)	(ref)	(ref)	(ref)	(ref)	(ref)	(ref)
yes	10.25 (0.96—109.45)	1.29 (0.29—5.66)	0.25 (0.05—1.3)	**0.0 (.—.)**	(empty)	0.35 (0.01—14.51)	9.43E+07 (0.0—.)	(empty)
**Diabetes**								
No	(ref)	(ref)	(ref)	(ref)	(ref)	(ref)	(ref)	(ref)
Yes	1.06 (0.1—11.91)	0.88 (0.21—3.71)	1.28 (0.26—6.33)	**0.0 (.—.)**	1.2E-20 (.—.)	0.53 (0.05—5.5)	1.4e+07 (0.0—.)	2.0E+163 (.—.)
Unknown	(empty)	0.22 (0.01—5.99)	(empty)	(empty)		(empty)	(empty)	
**Dyslipidaemia**								
No	(ref)	(ref)	(ref)	(ref)	(ref)	(ref)	(ref)	(ref)
Yes	1.05 (0.12—9.23)	0.43 (0.11—1.75)	2.43 (0.38—15.56)	**0.0 (.—.)**	1.4E+23 (.—.)	1 (0.05—21.22)	32.63 (0.17—6363.24)	1.5E-127 (.—.)
missing data	0.41 (0.02—7.53)	3.39 (0.18—65.18)	0.14 (0.01—2.05)	(empty)		(empty)	109.64 (0.06—205197.3)	(empty)
**HIV status**								
Negative	(ref)	(ref)	(ref)	(ref)	(ref)	(ref)	(ref)	(ref)
Positive	0.85 (0.08—9.02)	0.6 (0.14—2.55)	2.5 (0.62—10.07)	.	5.5E+23 (.—.)	0.88 (0.05—17.25)	24.16 (0.59—992.53)	1.6E+105 (.—.)

For peer review only - http://bmjopen.bmj.com/site/about/guidelines.xhtml

Unknown	0.87 (0.15—5.16)	1.34 (0.52—3.45)	2.37 (0.6—9.4)	.	(empty)	0.75 (0.14—3.89)	157.01 (0.5—49479.48)	8.25E-77 (.—.)
**Hypertension**								
No	(ref)	(ref)	(ref)	(ref)	(ref)	(ref)	(ref)	(ref)
Yes	1.58 (0.23—10.99)	0.51 (0.13—1.97)	0.68 (0.13—3.57)	0.0 (.—.)	4.4E-38 (.—.)	0.44 (0.04—5.13)	2.37 (0.06—95.27)	6.1E+132 (.—.)
**CVDRF**								
2	0.53 (0.05—5.26)	0.18 (0.04—0.8)	2.39 (0.45—12.74)	.				(ref)
3	(ref)	(ref)	(ref)	(ref)	(ref)	(ref)	(ref)	2.43E+94 (.—.)
4	2.88 (0.28—29.86)	2.42 (0.57—10.31)	0.62 (0.1—3.8)	.	1.4E-22 (.—.)	0.99 (0.04—23.46)	27.92 (0.04—17774.42)	(omitted)
≥5		(empty)	21.02 (0.37—1193.6)	(empty)	4.4e-07 (.—.)	1.95 (0.01—423.84)	1.44 (0.0—2718.99)	1.14E-57 (.—.)

For peer review only

STROBE Statement—Checklist of items that should be included in reports of ***cross-sectional studies***

	Item No	Recommendation	Page No
**Title and abstract**	1	(*a*) Indicate the study's design with a commonly used term in the title or the abstract	1
		(*b*) Provide in the abstract an informative and balanced summary of what was done and what was found	2
**Introduction**			
Background/rationale	2	Explain the scientific background and rationale for the investigation being reported	4
Objectives	3	State specific objectives, including any prespecified hypotheses	5
**Methods**			
Study design	4	Present key elements of study design early in the paper	5
Setting	5	Describe the setting, locations, and relevant dates, including periods of recruitment, exposure, follow-up, and data collection	5
Participants	6	(*a*) Give the eligibility criteria, and the sources and methods of selection of participants	5
Variables	7	Clearly define all outcomes, exposures, predictors, potential confounders, and effect modifiers. Give diagnostic criteria, if applicable	6-7
Data sources/ measurement	8*	For each variable of interest, give sources of data and details of methods of assessment (measurement). Describe comparability of assessment methods if there is more than one group	6
Bias	9	Describe any efforts to address potential sources of bias	11
Study size	10	Explain how the study size was arrived at	7
Quantitative variables	11	Explain how quantitative variables were handled in the analyses. If applicable, describe which groupings were chosen and why	7
Statistical methods	12	(*a*) Describe all statistical methods, including those used to control for confounding	7
		(*b*) Describe any methods used to examine subgroups and interactions	7
		(*c*) Explain how missing data were addressed	
		(*d*) If applicable, describe analytical methods taking account of sampling strategy	7
		(*e*) Describe any sensitivity analyses	
**Results**			
Participants	13*	(a) Report numbers of individuals at each stage of study—eg numbers potentially eligible, examined for eligibility, confirmed eligible, included in the study, completing follow-up, and analysed	8
		(b) Give reasons for non-participation at each stage	
		(c) Consider use of a flow diagram	16
Descriptive data	14*	(a) Give characteristics of study participants (eg demographic, clinical, social) and information on exposures and potential confounders	8
		(b) Indicate number of participants with missing data for each variable of interest	8
Outcome data	15*	Report numbers of outcome events or summary measures	8
Main results	16	(*a*) Give unadjusted estimates and, if applicable, confounder-adjusted estimates and their precision (eg, 95% confidence interval). Make clear which confounders were adjusted for and why they were included	9

For peer review only - http://bmjopen.bmj.com/site/about/guidelines.xhtml

		(*b*) Report category boundaries when continuous variables were categorized	
		(*c*) If relevant, consider translating estimates of relative risk into absolute risk for a meaningful time period	
Other analyses	17	Report other analyses done—eg analyses of subgroups and interactions, and sensitivity analyses	9
**Discussion**			
Key results	18	Summarise key results with reference to study objectives	9
Limitations	19	Discuss limitations of the study, taking into account sources of potential bias or imprecision. Discuss both direction and magnitude of any potential bias	2 & 12
Interpretation	20	Give a cautious overall interpretation of results considering objectives, limitations, multiplicity of analyses, results from similar studies, and other relevant evidence	10-12
Generalisability	21	Discuss the generalisability (external validity) of the study results	10-12
**Other information**			
Funding	22	Give the source of funding and the role of the funders for the present study and, if applicable, for the original study on which the present article is based	8

*Give information separately for exposed and unexposed groups.

Note: An Explanation and Elaboration article discusses each checklist item and gives methodological background and published examples of transparent reporting. The STROBE checklist is best used in conjunction with this article (freely available on the Web sites of PLoS Medicine at http://www.plosmedicine.org/, Annals of Internal Medicine at http://www.annals.org/, and Epidemiology at http://www.epidem.com/). Information on the STROBE Initiative is available at www.strobe-statement.org.

For peer review only

For peer review only - http://bmjopen.bmj.com/site/about/guidelines.xhtml

BMJ Open

The prevalence and risk factors of lower extremity disease in high risk groups in Malawi: a stratified cross-sectional study

Journal:	*BMJ Open*
Manuscript ID	bmjopen-2021-055501.R1
Article Type:	Original research
Date Submitted by the Author:	06-Mar-2022
Complete List of Authors:	Kasenda, Stephen; Malawi Epidemiology and Intervention Research Unit<br>Crampin, Amelia; Malawi Epidemiology and Intervention Research Unit; London School of Hygiene & Tropical Medicine<br>Davies, Justine ; King's College London, Global Health<br>Malava, Jullita; Malawi Epidemiology and Intervention Research Unit<br>Manganizithe, Stella; Malawi Epidemiology and Intervention Research Unit<br>Kumambala, Annie; Malawi Epidemiology and Intervention Research Unit<br>Sandford, Becky; Guy's and St Thomas' NHS Foundation Trust
**Primary Subject Heading**:	Epidemiology
Secondary Subject Heading:	Cardiovascular medicine
Keywords:	EPIDEMIOLOGY, CARDIOLOGY, Cardiac Epidemiology < CARDIOLOGY, Hypertension < CARDIOLOGY

SCHOLARONE™
Manuscripts

I, the Submitting Author has the right to grant and does grant on behalf of all authors of the Work (as defined in the below author licence), an exclusive licence and/or a non-exclusive licence for contributions from authors who are: i) UK Crown employees; ii) where BMJ has agreed a CC-BY licence shall apply, and/or iii) in accordance with the terms applicable for US Federal Government officers or employees acting as part of their official duties; on a worldwide, perpetual, irrevocable, royalty-free basis to BMJ Publishing Group Ltd ("BMJ") its licensees and where the relevant Journal is co-owned by BMJ to the co-owners of the Journal, to publish the Work in this journal and any other BMJ products and to exploit all rights, as set out in our licence.

The Submitting Author accepts and understands that any supply made under these terms is made by BMJ to the Submitting Author unless you are acting as an employee on behalf of your employer or a postgraduate student of an affiliated institution which is paying any applicable article publishing charge ("APC") for Open Access articles. Where the Submitting Author wishes to make the Work available on an Open Access basis (and intends to pay the relevant APC), the terms of reuse of such Open Access shall be governed by a Creative Commons licence – details of these licences and which Creative Commons licence will apply to this Work are set out in our licence referred to above.

Other than as permitted in any relevant BMJ Author's Self Archiving Policies, I confirm this Work has not been accepted for publication elsewhere, is not being considered for publication elsewhere and does not duplicate material already published. I confirm all authors consent to publication of this Work and authorise the granting of this licence.

For peer review only

The prevalence and risk factors of lower extremity disease in high risk groups in Malawi:_a stratified cross-sectional study

Author and author affiliations

Stephen Kasenda[1], Amelia C. Crampin[1,2,3], Justine Davies[4,5,6*], Jullita Kenala Malava[1], Stella Manganizithe[1], Annie Kumambala[1], Becky Sandford[7]

Author affiliations

[1] Malawi Epidemiology and Intervention Research Unit, Lilongwe, Malawi

[2] London School of Hygiene & Tropical Medicine, London, UK

[3] University of Glasgow, Institute of Health and Wellbeing, Glasgow, UK

[4] Institute of Applied Health Research, University of Birmingham, UK

[5] Centre for Global Surgery, Department of Global Health, Stellenbosch University, Cape Town

[6] Medical Research Council/Wits University Rural Public Health and Health Transitions Research Unit, Faculty of Health Sciences, School of Public Health, University of the Witwatersrand, Johannesburg, South Africa

[7] Guy's and St Thomas 'NHS Trust, London, UK

*Corresponding author

Author contact details and ORCID

SK: Stephen.kasenda@meiru.mw**;** 0000-0001-9208-4122

AC: mia.crampin@lshtm.ac.uk; 0000-0002-1513-4330

JD: J.Davies.6@bham.ac.uk; 0000-0001-6834-1838

JM: jullita.malava@meiru.mw;

SM: stellamanganizithe@gmail.com

AM: anniekumambala@gmail.com

BS: Becky.Sandford@gstt.nhs.uk; 0000-0001-6337-936X

For peer review only

For peer review

Abstract

Objective: Low and middle income countries face a disproportionate burden of cardiovascular diseases. However, amongst cardiovascular diseases, burden of and associations with lower extremity disease (peripheral arterial disease and/or diabetic foot disease) is neglected. We investigated the prevalence and factors associated with lower extremity disease (LED) among individuals known to have cardiovascular risk factors in Malawi, a low income country with a significant prevalence of cardiovascular disease risk factors.

Design: This was a stratified cross-sectional study.

Setting: This study was conducted in urban Lilongwe area 25, and the rural Karonga Health and Demographic Surveillance Site.

Participants: Participants were at least 18 years old and had been identified to have two or more known risk factors for cardiovascular disease (CVDRF).

Main outcome measures: Lower extremity disease—determined by the presence of one of the following: neuropathy (as assessed by a 10g monofilament), arterial disease (absent

peripheral pulses, claudication as assessed by the Edinburgh claudication questionnaire, or ankle brachial pulse index <0.9), previous amputation or ulceration of the lower limbs.

Results: There were 806 individuals enrolled into the study. Mean age was 52.5 years; 53.5% of participants were male (n=431) and 56.7% (n=457) were from the rural site. Nearly a quarter (24.1%; 95%: 21.2—27.2) of the participants had at least one symptom or sign of LED. 12.8% had neuropathy, 6.7% had absent pulses, 10.0% had claudication, 1.9% had ABPI<0.9, 0.9% had an amputation, and 1.1% had lower limb ulcers. LED had statistically significant association with increasing age, urban residence, and use of indoor fires.

Conclusions: This study demonstrated that a quarter of individuals with two or more CVDRFs have evidence of LED and 2.4% have an amputation or signs of limb threatening ulceration or amputation. Further epidemiological and health systems research is warranted to prevent LED and limb loss.

Strengths and limitations of this study

- Little is known about lower extremity disease in Malawi
- The use of multiple parameters to ensured more accurate estimation of LED prevalence
- calcification in vessels may result in false negative ABPI results

Data availability statement: Data are available on reasonable request

Word count: 3000

Supplemental material: For detailed data on the distribution

Keywords: Malawi, peripheral artery disease, cardiovascular diseases, HIV, hypertension, diabetes mellitus, obesity

Funding: This work was supported by King's Health Partners and Development Challenge Fund (MC_PC_16048). The baseline survey was funded by the Wellcome Trust (098610/Z/12/Z and 098610/B/12/A). The funders had no role in the study design; collection, analysis, and interpretation of data; or report writing. The corresponding author had full access to the data and the final responsibility to submit for publication.

Competing interests: None declared.

Author contributions:

SK: data collection, analysis, drafting and revising manuscript,

AC: data collection, design, analysis and revising manuscript

JD: design, analysis and revising manuscript

SM: data collection and revising manuscript

AM: data collection and revising manuscript

BS: design, analysis and revising manuscript

Introduction

Peripheral vascular disease and peripheral neuropathies (including the spectrum of diabetic foot disease) share a common group of overlapping risk factors, namely cardiovascular risk factors (CVDRFs) such as hypertension, diabetes, smoking, obesity, and hypercholesterolaemia. Infectious agents, particularly HIV also contribute to increased risk of peripheral vascular disease(1). Resources required to optimise preventative strategies, treat complications, and offer rehabilitation after surgery for peripheral vascular disease and peripheral neuropathies disease are largely the same whether the underlying disease process was predominantly ischaemic or neuropathic. As such, we have considered these conditions as one entity representing a spectrum of problems under the term 'Lower extremity disease" (LED). Individuals with LED are at risk of lower limb amputation, particularly in cases where there is significant ischaemic necrosis infection. Amputation can be a life-saving procedure, relieving pain and sepsis, but may also be associated with negative physical, psychological, social, and economic consequences (2,3).

With increasing prevalence of CVDRF in lower and middle income countries (LMIC) superimposed on a background prevalence of chronic infections, it is strongly suspected that associated risk of lower limb amputation will become increasingly important for individuals, families, health systems, and economies(4). Evidence from LMICs is lacking, but suggestive that burden of amputations related to LED conditions will grow. Almost 70% of people with peripheral arterial disease live in LMICs; African countries in particular show a high population-prevalence of peripheral arterial disease (e.g. 33% in a population of people >65 years old in Central Africa)(5–7). Studies in people who have managed to access CVDRF clinics in referral hospitals also show a large number of people have peripheral artery disease; for example, in Ghana, 27% of nearly 1000 people with diabetes had clinically measurable peripheral arterial disease(8). In our pilot study of 191 patients attending a hospital clinic in Blantyre, Malawi, peripheral arterial disease was seen in 8.5% of 45-64 year olds and 17% of people aged 65 and over(9). Reliable data on population prevalence of diabetic foot ulceration are lacking, however, studies show that in people with diabetes who have accessed services, prevalence ranged from 4.0 to 9.9%; it could be far higher if people who had not accessed services were included(10).

To develop health systems to manage and prevent LED and its consequences requires knowledge of its prevalence. The aim of this study was to investigate the population prevalence of LED amongst high risk individuals in rural and urban Malawi.

Methods

Study setting

The study was conducted in Area 25 in urban Lilongwe, and the rural Karonga Health and Demographic Surveillance Site (HDSS). The Karonga site has a population of approximately 45,000 and the economy is mainly subsistence farming. Area 25 (Lilongwe) has an approximate total population of 65,000 with a mixed economy.

Study design and participants

We conducted a stratified cross-sectional study of prevalence of "lower extremity disease" (LED) defined as neuropathy and/or vasculopathy in all adults of at least 18 years age with two or more cardiovascular disease risk factors. This population was selected to ensure a reasonable sample size of people at highest risk of lower extremity disease.

Identification of participants

We included all eligible participants who were at least 18 years old and had been identified to have two or more known risk factors for LED during a population wide survey of cardio-metabolic conditions conducted between 2013 and 2017 in the study areas (11). Presence of hypertension, diabetes mellitus, HIV, tobacco smoking, obesity, and age at least 40 years were used to characterise high risk individuals.

Data collection and definitions

Data on participant socio-demographic characteristics (sex, date of birth, marital status, highest attained education, use of indoor fire, and household wealth) and presence of CVDRFs (history of smoking, diabetes mellitus, hypertension, dyslipidaemia, and HIV status using previous laboratory diagnosis) were extracted from the initial baseline study. Methods used to capture data and definitions used in that baseline survey have been published elsewhere (11). In brief, diabetes mellitus was defined as a fasting blood glucose of at least 7.0 mmol/L (as determined by a Beckman Coulter AU480 Chemistry Analyser) or self-report of a previous diagnosis of the condition by a health professional regardless of the drug

For peer review only

history. Hypertension was defined as a blood pressure of at least 140/90 mmHg or currently on antihypertensive medication. Participants with a body mass index (BMI) of at least 30 kg/m^2 were classified as having obesity. Participants were defined as smokers if they reported smoking at least one cigarette per day in the immediate past six months preceding the survey. Participants were defined as HIV-positive if they had previously been tested antibody positive by the study team or self-reported HIV-positivity.

For the current study, participants were interviewed and examined in their own homes and data collectors were blind to the participant's previous medical history. In cases where the potential participant was missed during the first visit and it was established that they had neither left the study area nor died, the household was visited at least three times before declaring the potential participant as "missed". All participants were interviewed to determine if they had any changes in cardiovascular disease risk profile. The Edinburgh claudication questionnaire was used to determine the presence of claudication and participants who reported the presence of clinical CVDRFs were asked additional questions to regarding access to care for the relevant conditions they have.

Participants were examined to determine the presence of leg or foot ulcers, palpable peripheral pulses (brachial, posterior tibial, and dorsalis pedis), and/ or lower limb amputations in both extremities. Neuropen® 10g monofilaments were used to test for presence of peripheral neuropathy on the plantar surfaces great toe as well as over the metatarsal head of the great toe, 3rd and 5th toes of both feet. Portable sphygmomanometers (Welch Allyn DS55 Durashock hand aneroid model) and handheld continuous wave Doppler ultrasound devices (Bistos Hi.dop BT-200 vascular Doppler with 8 MHZ probe) were used to determine Ankle Brachial Pressure Index (ABPI). ABPI for each participant was determined according to the methods recommended by Aboyans et. al(12,13). A low ABPI reading suggests peripheral arterial disease, with the blood pressure reduced at the ankle compared with the arm which implies stenosis within the arterial tree supplying the lower limbs. Each participant was considered to have low ABPI if they had ABPI less than 0.9 on any or both sides.

Outcomes

The primary outcome of interest was the presence of any symptom or sign suggestive of lower extremity disease, termed "LED prevalence" (history of claudication, limb ulcers, loss of sensation on the plantar surfaces of feet, or Ankle Brachial Pressure Index [ABPI] less than 0.9). Secondary outcomes were these as individual outcomes.

Statistical analysis

Given the dearth of information on the prevalence of LED in the local setting, we based our sample size calculation on pilot data from a study of 90 people with diabetes living in the Malawi HDSS, which suggested that the prevalence of LED was around 10%. Considering people with diabetes to be at high risk and similar to those chosen for our study, we calculated a sample of 912 people would be required to detect a prevalence of LED of 10% with a CI of 6-14%.

Prevalence of the primary and secondary outcomes, socio-demographic characteristics of the study participants, and CVDRFs are described as mean (SD) and count (%). Logistic regression and likelihood ratio tests were done to determine associations between the primary outcome and independent variables. Socio-demographic characteristics and presence of CVDRFs were considered as a priori independent variables of interest in our analysis. The association between number of CVDRFs and the crude prevalence of the outcome of interest was also investigated.

All variables of interest were added to the regression model and likelihood ratio testing was done to determine the presence of interactions between the independent variables. Stratified analyses of the full regression model were done for all variables that were identified as having statistically significant interactions. All statistical analysis was done using Stata 15 statistical package.

Role of the funding source

Funding for this study was provided by King's Health Partners and Development Challenge Fund (MC_PC_16048), King's College London, UK. The baseline survey was funded by the Wellcome Trust (098610/Z/12/Z and 098610/B/12/A). The funders had no role in the study design; collection, analysis, and interpretation of data; or report writing. The corresponding author had full access to the data and the final responsibility to submit for publication.

Ethical approvals

Ethical approval for the baseline survey was granted by the Malawi National Health Sciences Research Committee (NHRSC) protocol number #1072, and London School of Hygiene and Tropical Medicine (LSHTM) Ethics Committee protocol number #6303. Ethical approval for the lower extremity disease study was granted by NHSRC approval number #2090 and King's College London BDM Research Ethics Panel (reference: LRS-17/18-7983). Data was collected from potential participants who gave written informed consent. Additional consent

was sought from the participants/span> for utilization of their previously obtained HIV results in data analysis for this study.

Patient and public involvement

There was no patient and public involvement in this study.

Results

A total of 829 people were visited, of whom 806 (97.2%) consented to be recruited (Figure 1). Most of the participants were at least 50 years age (mean age 52.5; 95%CI: 51.7—53.3), male (53.5%; n=431), from the rural site (56.7%; n=457), employed (70.6%; n=569), married (74.1%; n=597), and had no more than primary education (61.1%; n=493). Cooking with biomass fuel was reported by 94.8% (n=764) of the participants (Table 1). The majority 88.1% (n=710) of participants, had at least two clinical risk factors for LED (hypertension, obesity, HIV, diabetes, and dyslipidaemia), and the remaining 96 participants were aged at least 40 years and had a history of smoking tobacco (Figure 2 & Table 1). The mean age of the rural participants was higher than that of the urban participants (53.5 years [95%CI: 52.4—54.6] compared to 51.2 years [95%CI: 50.1—52.3]; P=0.005). Missing data accounted for not more than 6.1% of all the data on any variable.

Nearly a quarter (24.9%; 95%: 22.0—28.1) of the participants had at least one symptom or sign of LED (Table 2). Peripheral neuropathy (N=103; 12.8%) and claudication (N=81; 10 %) were the most common features of LED, and signs of limb threatening disease [i.e. ulcers (N=13; 1.1 %) and lower limb amputations (N=7; 0.9%)] were the least prevalent, affecting 2.4% (n=19) of participants. ABPI less than 0.9 was recorded among 32 (4.0%) participants, and 391 (48.5%) had ABPI greater than 1.2. (Appendix table 1) Four participants had ABPI less than 0.9 on one side & ABPI greater than 1.2 on the contralateral side. In view of the reduced ABPI <0.9, which is more likely to be a true positive, these individuals were categorised as having low ABPI. Those with ABPI >1.2 on both sides were grouped together with participants with ABPI between 0.9 and 1.2. At least 25% of participants with each CVDRF, apart from smoking, had LED. LED prevalence rates of over 30% were recorded among participants who were urban residents, female, aged over 50 years, single, indoor users of biomass fuel, or homemakers. The prevalence of LED was directly proportional to

the number of CVDRFs with the highest prevalence of LED [35.6%; 95%CI: 23.6—49.1] being recorded among participants that had five or more risk factors (table 2).

In the full regression model (figure 3), statistically significantly increased odds of LED were found in participants aged at least 50 years ((OR: 1.19; 95%CI: 1.22—2.99) among those aged 50-60, and OR: 2.33; 95%CI: 1.43—3.8 among those over 60 years age) and urban residents (OR: 1.76; 95%CI: 1.05—2.94) (Appendix table 2). Cooking with methods other than biomass burning was negatively associated with LED [OR: 0.19; 95%CI: 0.05—0.69]. The following sets of variables had some statistically significant interactions that affected the odds of the LED: (I) sex and history of smoking, (II) obesity and highest attained education, and (III) obesity and participant's wealth quintile, as computed in the full model. The results of these interactions are presented in Appendix tables 3- 6.

Discussion

The aim of this study was to describe the prevalence of lower extremity disease (LED) amongst individuals with known cardiovascular disease risk factors (CVDRF) in rural and urban Malawi. Of 806 individuals studied, a large proportion had at least one sign of LED, with claudication and peripheral neuropathy being the most common. Prevalence of LED was directly related to the number of risk factors with advancing age, urban living, and poverty demonstrating the greatest association. Although amputations and active ulceration were less frequently seen, 2.5% of individuals in this study had these conditions which are indicative of active or previous limb threatening disease.

Provision of health care services to manage cardiovascular risk factors or care for patients with LED are limited in Malawi and tend to be focused around large urban centres(14). Health literacy also tends to be poor amongst those living with chronic conditions, with patients and families often understanding little about their condition or the longer term consequences of inadequate management (15). Provision of surgical services for the management of LED (e.g. vascular reconstructive techniques, amputation and rehabilitation services) are limited throughout southern Africa and this field has not been viewed as a priority amongst competing healthcare development needs(16,17).

In poorly resourced health systems where people present late with disease, amputation rates and subsequent mortality is high. For example, a small study in Tanzania showed that 33% of people admitted to hospital with diabetic foot disease were managed by amputation and mortality was nearly 50%(18). By comparison, in the UK 17% of people presenting with

disease underwent amputation within 1 year of presentation with no reported mortality (19). After limb-loss, poor post-operative care and the dearth of services for rehabilitation, prostheses, and mobility aids result in further mortality and morbidity (20). The devastating effects are tragic given there are simple and cheap strategies – including preventing and treating CVDRF and regular monitoring for signs of LED in high risk individuals with early referral to specialist services - to reduce deleterious outcomes.

Although this is one of the few studies to look at population prevalence of LED, (7) the findings of this study are in keeping with previously published work(8,21). For example, population prevalence of vascular disease has been reported at over 30% in those over 65 years (7).

An interesting 'U 'shaped association between LED and wealth was observed, with the greatest prevalence of disease in the poorest and the wealthiest groups. The prevalence of hypertension and diabetes in these groups was also significantly higher at the extremes of wealth than in the middle 3 quintiles, which may go some way to explaining the observations. There may be other factors, beyond the scope of this study to characterise, such as lack of access to skilled healthcare services for optimum management of these comorbidities in the poorer communities (22), and potentially other social drivers towards worse ill-health such as sedentary lifestyle and westernised dietary habits amongst the wealthier group, which somewhat negate improved access to healthcare for those who are more wealthy (23–25). It will be important to fully understand these associations in future work in order to understand which interventions may be the most effective in preventing LED amongst different population groups.

Smoking status in this study was not shown to be a significant factor associated with LED, in contrast to a well-established body of evidence from high income countries(26). The reasons for this are not clear, however smoking is not as common in Malawi as in some high income countries and the cut off for being defined as a smoker in this study was more than 6 cigarettes per week, which is a relatively low level of consumption.

This was a large scale population based study, highlighting those at risk of limb loss amongst a group with known CVDRF. In focusing on population level data, the study reduces the potential for bias towards those who have already engaged with healthcare services. This is an advantage over much of the published literature to date, and provides a greater understanding of the scale of LED across different population groups in Malawi. The use of multiple approaches to evaluate the presence of LED is also a strength of this study, recognising that no single assessment is either sensitive or specific in isolation(4).

There are potential limitations to the conclusions to be drawn from this study. The use of ABPI and the Edinburgh Claudication Questionnaire as surrogate markers for peripheral vascular disease, whilst both validated techniques, do have drawbacks. Claudication, in itself, may never progress to represent a limb threatening condition, however can be a good marker of more generalised cardiovascular ill-health and therefore highlight opportunities for optimisation of best medical therapy in order to reduce cardiovascular mortality(27). ABPI may be confounded by calcification in vessels, resulting in falsely elevated ratios; this is particularly seen amongst diabetic individuals. The inclusion of those with ABPI>1.2 into the group with ABPI 0.9-1.2 may have resulted in some false negative results, neglecting to report arterial disease which was in fact present. Whilst toe brachial pressure index (TBPI) may be a more reliable observation in this group(28), there is little in the published data on TBPI studies in SSA and therefore interpretation of the findings from this study in the context of previously published work would have been more limited had this technique been utilised. We also did not enquire about the etiology of ulcers hence it is not possible to establish the etiology of the ulcers. Finally, we failed to achieve the sample size for this study, however the proportion of participants affected by LED was far greater than we had estimated and would have required a lower number of participants to reliably detect. In a retrospective calculation, 460 participants would have been required to detect a prevalence of LED of 24% with the same width of confidence interval as in our initial power calculation.

In conclusion, this study demonstrated that 1 in 4 individuals with two or more cardiovascular risk factors have evidence of lower extremity disease and 2.5% already had active signs of limb threatening ulceration or previous amputation. An interesting U-shaped association with wealth was observed. Future work will need to explore the social determinants of health in relation to LED in order to guide health system interventions appropriately; the solutions may be complex and it is likely that there will not be a ‘one size fits all ’approach to preventing limb loss in this region. It is likely that both therapeutic and preventive services will have to be developed in parallel.

References

1. Pillay B, Ramdial P, Naidoo D. HIV-associated large-vessel vasculopathy: a review of the current and emerging clinicopathological spectrum in vascular surgical practice: review article. Cardiovasc J Afr. 2015 Apr 30;26(2):70–81.

2. Madsen UR, Baath C, Berthelsen CB, Hommel A. Age and health-related quality of life, general self-efficacy, and functional level 12 months following dysvascular major lower limb amputation: a prospective longitudinal study. Disabil Rehabil. 2019 Nov 20;41(24):2900–9.
3. Taghipour H, Moharamzad Y, Mafi AR, Amini A, Naghizadeh MM, Soroush MR, et al. Quality of Life Among Veterans With War-Related Unilateral Lower Extremity Amputation: A Long-Term Survey in a Prosthesis Center in Iran: J Orthop Trauma. 2009 Aug;23(7):525–30.
4. Johnston LE, Stewart BT, Yangni-Angate H, Veller M, Upchurch GR, Gyedu A, et al. Peripheral Arterial Disease in Sub-Saharan Africa: A Review. JAMA Surg. 2016 Jun 1;151(6):564.
5. Fowkes FGR, Rudan D, Rudan I, Aboyans V, Denenberg JO, McDermott MM, et al. Comparison of global estimates of prevalence and risk factors for peripheral artery disease in 2000 and 2010: a systematic review and analysis. The Lancet. 2013 Oct;382(9901):1329–40.
6. Joshi R, Jan S, Wu Y, MacMahon S. Global Inequalities in Access to Cardiovascular Health Care. J Am Coll Cardiol. 2008 Dec;52(23):1817–25.
7. Guerchet M, Aboyans V, Mbelesso P, Mouanga AM, Salazar J, Bandzouzi B, et al. Epidemiology of Peripheral Artery Disease in Elder General Population of Two Cities of Central Africa: Bangui and Brazzaville. Eur J Vasc Endovasc Surg. 2012 Aug;44(2):164–9.
8. Yeboah K, Puplampu P, Ainuson J, Akpalu J, Gyan B, Amoah AGB. Peripheral artery disease and exertional leg symptoms in diabetes patients in Ghana. BMC Cardiovasc Disord. 2016 Dec;16(1):1–9.
9. Chisala P, Sandford B. Peripheral arterial disease among high-risk patients at a tertiary hospital in Blantyre, Malawi: A 2-phase observational study. East Cent Afr J Surg. 2020;25(3).
10. Okello S, Millard A, Owori R, Asiimwe SB, Siedner MJ, Rwebembera J, et al. Prevalence of lower extremity Peripheral artery disease among adult diabetes patients in Southwestern Uganda. BMC Cardiovasc Disord. 2014 Dec;14(1):75.
11. Price AJ, Crampin AC, Amberbir A, Kayuni-Chihana N, Musicha C, Tafatatha T, et al. Prevalence of obesity, hypertension, and diabetes, and cascade of care in sub-Saharan Africa: a cross-sectional, population-based study in rural and urban Malawi. Lancet Diabetes Endocrinol. 2018 Mar;6(3):208–22.

12. Aboyans V. The intra- and interobserver variability of ankle–arm blood pressure index according to its mode of calculation. J Clin Epidemiol. 2003 Mar;56(3):215–20.
13. Aboyans V, Criqui MH, Abraham P, Allison MA, Creager MA, Diehm C, et al. Measurement and Interpretation of the Ankle-Brachial Index: A Scientific Statement From the American Heart Association. Circulation. 2012 Dec 11;126(24):2890–909.
14. Chikowe I, Mwapasa V, Kengne AP. Analysis of rural health centres preparedness for the management of diabetic patients in Malawi. BMC Res Notes. 2018 Dec;11(1):267.
15. Assayed AA, Muula AS, Nyirenda MJ. The quality of care of diabetic patients in rural Malawi: A case of Mangochi district. Malawi Med J. 2014;6.
16. Veller MG. Education in vascular surgery–critical issues: A Southern African perspective. J Vasc Surg. 2008 Dec 1;48(6):84S-86S.
17. Magnusson L, Ahlström G, Ramstrand N, Fransson EI. Malawian prosthetic and orthotic users 'mobility and satisfaction with their lower limb assistive device. J Rehabil Med. 2013 Apr;45(4):385–91.
18. Gulam-Abbas Z, Lutale JK, Morbach S, Archibald LK. Clinical outcome of diabetes patients hospitalized with foot ulcers, Dar es Salaam, Tanzania. Diabet Med. 2002 Jul;19(7):575–9.
19. Paisey RB, Abbott A, Levenson R, Harrington A, Browne D, Moore J, et al. Diabetes-related major lower limb amputation incidence is strongly related to diabetic foot service provision and improves with enhancement of services: peer review of the South-West of England. Diabet Med. 2018 Jan;35(1):53–62.
20. Guest JF, Fuller GW, Vowden P. Diabetic foot ulcer management in clinical practice in the UK: costs and outcomes. Int Wound J. 2018;15(1):43–52.
21. Zhang P, Lu J, Jing Y, Tang S, Zhu D, Bi Y. Global epidemiology of diabetic foot ulceration: a systematic review and meta-analysis. Ann Med. 2017 Feb 17;49(2):106–16.
22. Angwenyi V, Aantjes C, Kajumi M, De Man J, Criel B, Bunders-Aelen J. Patients experiences of self-management and strategies for dealing with chronic conditions in rural Malawi. Van Bogaert P, editor. PLOS ONE. 2018 Jul 2;13(7):e0199977.
23. Geldsetzer P, Manne-Goehler J, Marcus M-E, Ebert C, Zhumadilov Z, Wesseh CS, et al. The state of hypertension care in 44 low-income and middle-income countries: a cross-sectional study of nationally representative individual-level data from 1·1 million adults. The Lancet. 2019 Aug;394(10199):652–62.

24. Manne-Goehler J, Geldsetzer P, Agoudavi K, Andall-Brereton G, Aryal KK, Bicaba BW, et al. Health system performance for people with diabetes in 28 low- and middle-income countries: A cross-sectional study of nationally representative surveys. Wareham NJ, editor. PLOS Med. 2019 Mar 1;16(3):e1002751.

25. Kreatsoulas C, Frcpc SSA. The impact of social determinants on cardiovascular disease. 2010;26:6.

26. How Tobacco Smoke Causes Disease: The Biology and Behavioral Basis for Smoking-Attributable Disease. U.S. Department of Health and Human Services, Centers for Disease Control and Prevention, National Center for Chronic Disease Prevention and Health Promotion, Office on Smoking and Health; 2010.

27. Bevan GH, White Solaru KT. Evidence-Based Medical Management of Peripheral Artery Disease. Arterioscler Thromb Vasc Biol. 2020 Mar;40(3):541–53.

28. Williams DT, Harding KG, Price P. An Evaluation of the Efficacy of Methods Used in Screening for Lower-Limb Arterial Disease in Diabetes. Diabetes Care. 2005 Sep 1;28(9):2206–10.

For peer review only

Table 1: Summary of participant characteristics

Variables	N (%)	Both sites		Karonga (rural)		Lilongwe (urban)	
		men	women	men	women	men	women
**TOTAL**		**431**	**375**	**303**	**154**	**128**	**221**
**Age**							
Under 50	346(42.9)	199 (46.2)	147 (39.2)	138 (45.5)	51 (33.1)	61 (47.7)	96 (43.4)
50—60	239(29.7)	113 (26.2)	126 (33.6)	80 (26.4)	49 (31.8)	33 (25.8)	77 (34.8)
Over 60	221(27.4)	119 (27.6)	102 (27.2)	85 (28.1)	54 (35.1)	34 (26.6)	48 (21.7)
**Wealth quintiles**							
poorest	108 (13.4)	41 (9.5)	67 (17.9)	34 (11.2)	51 (33.1)	7 (5.5)	16 (7.2)
second	150 (18.6)	83 (19.3)	67 (17.9)	80 (26.4)	46 (29.9)	3 (2.3)	21 (9.5)
third	139 (17.3)	85 (19.7)	54 (14.4)	77 (25.4)	29 (18.8)	8 (6.3)	25 (11.3)
fourth	191 (23.7)	103 (23.9)	88 (23.5)	66 (21.8)	18 (11.7)	37 (28.9)	70 (31.7)

wealthiest	218 (27.1)	119 (27.6)	99 (26.4)	46 (15.2)	10 (6.5)	73 (57)	89 (40.3)
**Marital status**							
single	209 (25.9)	54 (12.5)	155 (41.3)	36 (11.9)	82 (53.3)	18 (14.1)	73 (33.0)
married	597 (74.1)	377 (87.5)	220 (58.7)	267 (88.1)	72 (46.8)	110 (85.9)	148 (67.0)
**highest attained education**							
0—5yrs primary education	164 (20.4)	59 (13.7)	105 (28)	48 (15.8)	66 (42.9)	11 (8.6)	39 (17.7)
Standard 6-8	329 (40.8)	184 (42.7)	145 (38.7)	156 (51.5)	65 (42.2)	28 (21.9)	80 (36.2)
secondary	230 (28.5)	141 (32.7)	89 (23.7)	94 (31.0)	21 (13.6)	47 (36.7)	68 (30.8)
Post-secondary	83 (10.3)	47 (10.9)	36 (9.6)	5 (1.7)	2 (1.3)	42 (32.8)	34 (15.4)
**Occupation**							
homemaker	237 (29.4)	74 (17.2)	163 (43.5)	32 (10.6)	34 (22.1)	42 (32.8)	129 (58.4)
Farming/fishing	296 (36.7)	209 (48.5)	87 (23.2)	208 (68.7)	84 (54.6)	1 (0.8)	3 (1.36)
employed	273 (33.9)	148 (34.3)	125 (33.3)	63 (20.8)	36 (23.4)	85 (66.4)	89 (40.3)
**Indoor fire**							

no	42 (5.2)	26 (6.0)	16 (4.3)	17 (5.6)	6 (3.9)	9 (7.0)	10 (4.5)
Yes, inside the house	39 (4.8)	19 (4.4)	20 (5.3)	5 (1.7)	3 (2.0)	14 (10.9)	17 (7.7)
Yes, but in a separate kitchen	725 (90.0)	386 (89.6)	339 (90.4)	281 (92.7)	145 (94.2)	105 (82)	194 (87.8)
**Smoking history**							
Non- smokers	487 (60.4)	130 (30.2)	357 (95.2)	72 (23.8)	142 (92.2)	58 (45.3)	215 (97.3)
Smokers	319 (39.6)	301 (69.8)	18 (4.8)	231 (76.2)	12 (7.8)	70 (54.7)	6 (2.7)
**Diabetes**							
Non-diabetic	502 (62.3)	324 (75.2)	324 (75.2)	251 (82.8)	88 (57.1)	73 (57)	90 (40.7)
Diabetic	273 (33.9)	87 (20.2)	87 (20.2)	42 (13.9)	62 (40.3)	45 (35.2)	124 (56.1)
Missing data	31 (3.9)	20 (4.6)	20 (4.6)	10 (3.3)	4 (2.6)	10 (7.8)	7 (3.2)
**Dyslipidaemia**							
yes	344 (42.7)	238 (55.2)	175 (46.7)	177 (58.4)	73 (47.4)	61 (47.7)	102 (46.2)
no	413 (51.2)	165 (38.3)	179 (47.7)	114 (37.6)	74 (48.1)	51 (39.8)	105 (47.5)
Missing data	49 (6.1)	28 (6.5)	21 (5.6)	12 (4.0)	7 (4.6)	16 (12.5)	14 (6.3)

**Hypertension**							
hypertensive	482 (59.8)	220 (51.0)	104 (27.7)	171 (56.4)	40 (26.0)	49 (38.3)	64 (29.0)
Not hypertensive	324 (40.2)	211 (49.0)	271 (72.3)	132 (43.6)	114 (74.0)	79 (61.7)	157 (71.0)
**Mean blood pressure (SD)**							
	139.1 (38.2)	136 (41.6)	142.6 (33.7)	137 (47)	149.1 (43.6)	133.7 (24.1)	138.1 (23.6)
**HIV**							
negative	413 (51.2)	237 (55.0)	176 (46.9)	170 (56.1)	80 (52.0)	67 (52.3)	96 (43.4)
positive	247 (30.7)	107 (24.8)	140 (37.3)	78 (25.7)	57 (37.0)	29 (22.7)	83 (37.6)
unknown	146 (18.1)	87 (20.2)	59 (15.7)	55 (18.2)	17 (11.0)	32 (25)	42 (19)
**Obesity**							
No	634 (78.7)	402 (93.3)	232 (61.9)	292 (96.4)	113 (73.4)	110 (85.9)	119 (53.9)
Yes	172 (21.3)	29 (6.7)	143 (38.1)	11 (3.6)	41 (26.6)	18 (14.1)	102 (46.2)

For peer review only - http://bmjopen.bmj.com/site/about/guidelines.xhtml

Table 2: prevalence of LED in total and by participant characteristics

Variables	N	LED prevalence (95% CI) n=194
**Total**	806	24.1 (21.2—27.2)
**Age**		
<50	346	15.9 (12.2—20.2)
50-60	239	27.6 (22.0—33.7)
>60	221	33.0 (26.9—39.7)
**Sex**		
Male	431	15.8 (12.5—19.6)
Female	375	33.6 (28.8— 38.6)
**Residence**		
Rural	457	17.5 (14.1—21.3)
Urban	349	32.7 (27.8—37.9)
**Wealth quintiles**		
poorest	108	32.4 (16.2—30.2)
second	150	22.7 (16.2—30.2)
third	139	15.8 (10.2—23.0)
fourth	191	24.1 (18.2—30.8)
wealthiest	218	26.1 (20.4—32.5)
**Marital status**		
Single	209	34.0 (27.6—40.8)
married	587	20.6 (17.4—24.1)
**Educational achievement**		
0-5 years primary education	164	25 (18.6—32.3)
Standard 6-8	329	25.5 (20.9—30.6)
Secondary	230	21.7 (16.6—27.6)
Post-secondary	83	22.9 (14.4—33.4)
**occupation**		
homemaker	237	37.1 (31.0—43.6)
Farming/ fishing	296	15.5 (11.6—20.2)
employed	273	22.0 (17.2—27.4)
**Indoor fire**		
none	42	7.1 (1.5—19.5)

For peer review only - http://bmjopen.bmj.com/site/about/guidelines.xhtml

Fire usually lit Inside the house	39	30.8 (17.0—47.6)
Fire usually lit in a separate kitchen area	725	24.7 (21.6—28.0)
**Any Smoking history**		
no	487	29.4 (25.4—33.6)
yes	319	16.0 (12.1—20.5)
**Diabetes**		
Non-diabetic	502	19.7 (16.3—23.5)
diabetic	273	33.7 (28.1—39.6)
Missing data	31	9.7 (2.0—25.8)
**Dyslipidaemia**		
no	413	22.8 (18.8—27.1)
yes	344	26.5 (21.9—31.5)
Missing data	49	18.4 (8.8—32.0)
**HIV**		
negative	413	23.0 (19.0—27.4)
positive	247	23.9 (18.7—29.7)
unknown	146	27.4 (20.3—35.4)
**hypertension**		
Not-hypertensive	324	21.9 (17.6—26.8)
hypertensive	482	25.5 (21.7—29.7)
**obesity**		
no	634	20.8 (17.7—24.2)
yes	172	36.0 (28.9—43.7)
**Number of risk factors**		
2	201	14.9 (10.3—20.6)
3	342	23.1 (18.7—27.9)
4	204	32.4 (26.0 —39.2)
≥5	59	32.2 (20.6—45.6)
**Prevalence of outcomes by risk factors**		
No diabetes and no hypertension	193	14.0 (25.6—42.4)
Diabetes but no hypertension	131	33.6 (25.6—42.4)

Hypertension but no diabetes	340	22.1 (17.8—26.8)
Hypertension and diabetes	142	33.8 (26.1—42.2)

Figure Legends

Figure 1: Flowcharts of study participation by site

Figure 2: three major risk factors and multi-morbidity across the study population

Figure 3: Coefficient plot of factors associated with lower extremity disease

For peer review only

FIGURE 1: Flowcharts of study participation by site

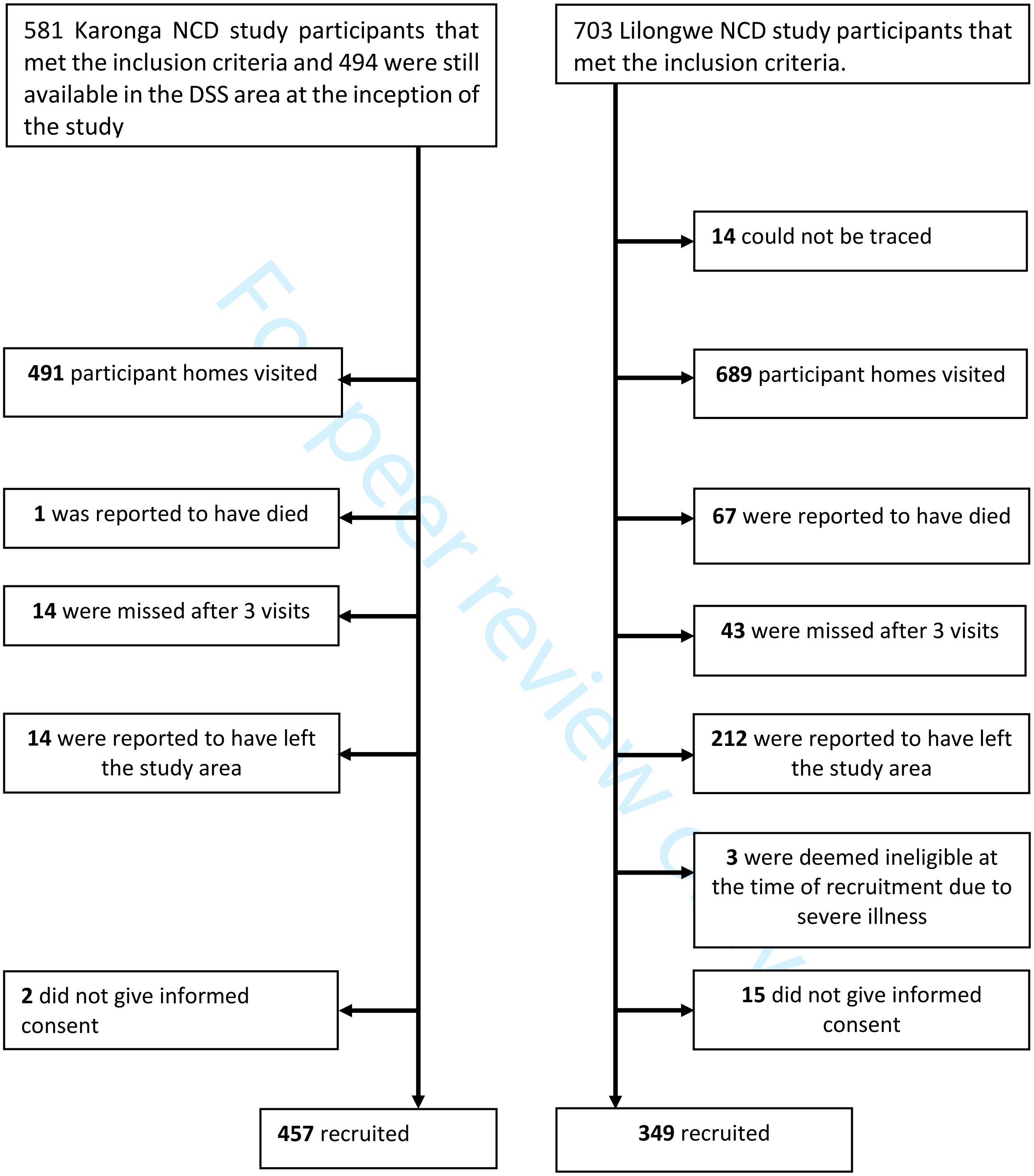

FIGURE 2: three major risk factors and multi-morbidity across the study population

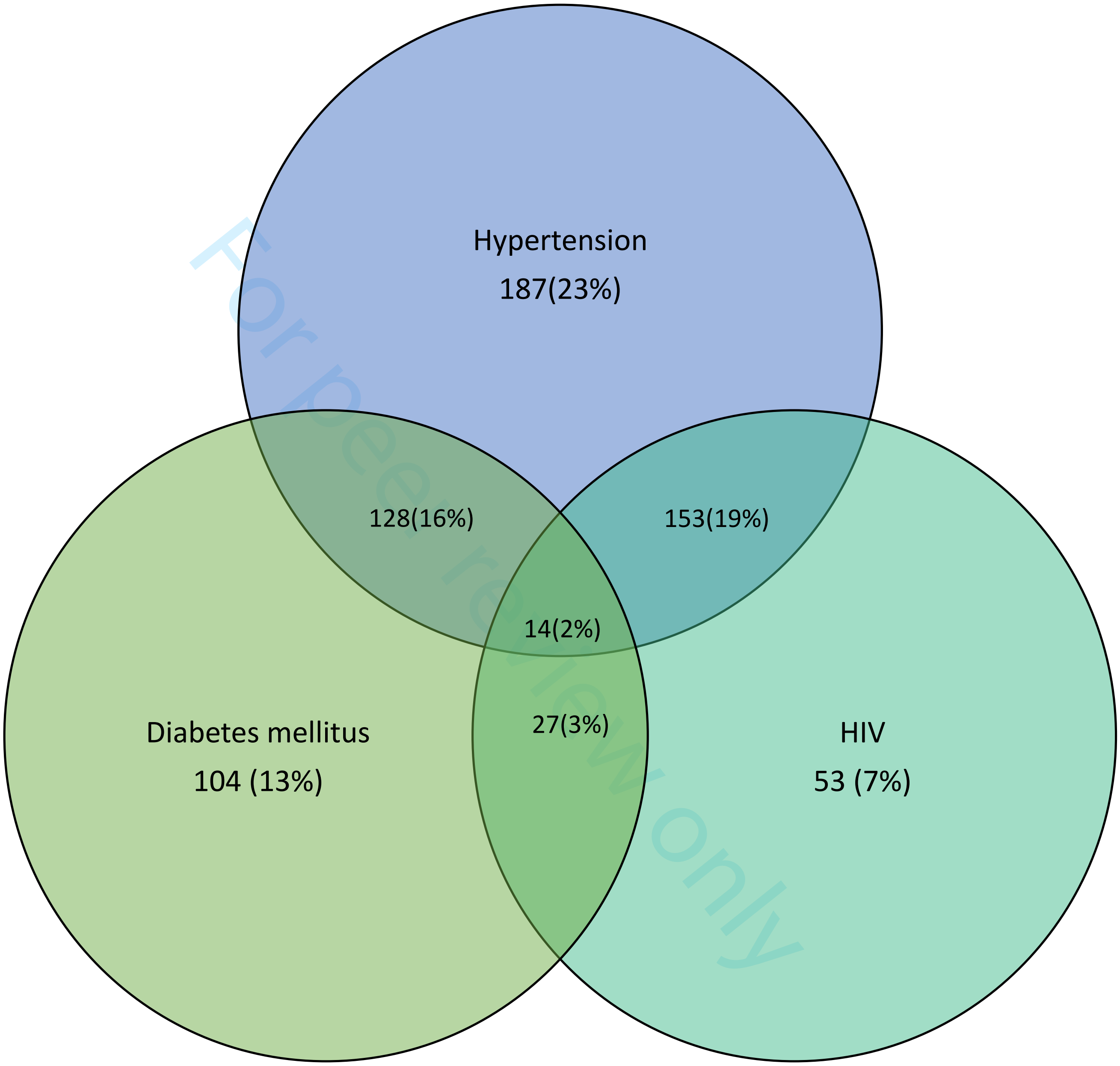

For peer review only - http://bmjopen.bmj.com/site/about/guidelines.xhtml

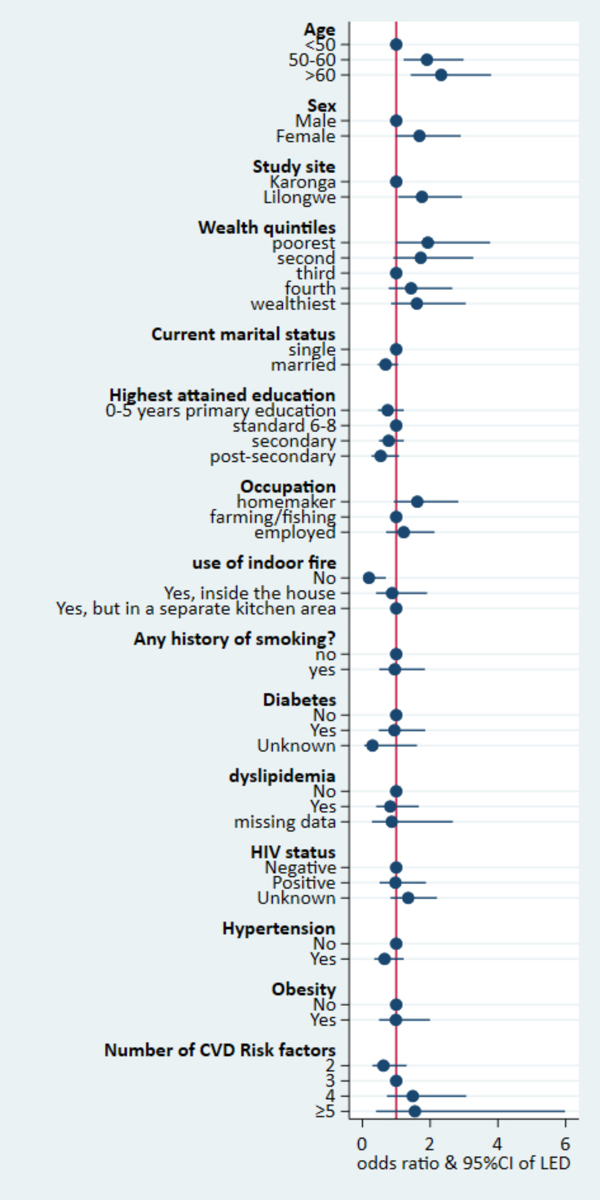

Coefficient plot of factors associated with lower extremity disease

125x250mm (96 x 96 DPI)

Appendices

Table 1: Crude prevalence of factors associated with LED

	N	OUTCOME						
		LED prevalence (95% CI) n=194	Absent pulse prevalence (95% CI) n=54	Amputation Prevalence (95% CI) n=7	Ulcer Prevalence (95% CI) n=13	Claudication Prevalence (95% CI) n=81	Neuropathy Prevalence (95% CI) n=103	ABPI<0.9 Prevalence (95% CI) n=18
**Total**								
	806	24.1 (21.2—27.2)	6.7 (5.1—8.7)	0.9 (0.3—1.8)	1.1 (0.4— 1.8)	10 (8.1—12.3)	12.8 (10.6—15.3)	1.9 (1.0—3.1)
**Age**								
<50	346	15.9 (12.2—20.2)	4.9 (2.9—7.8)	0.3 (0.0—1.6)	2.3 (1.0—4.5)	9.0 (6.2—12.5)	6.1 (3.8—9.1)	0.3 (0.0—1.6)
50-60	239	27.6 (22.0—33.7)	6.7 (3.9—10.6)	2.1 (0.7—4.8)	0.8 (0.1—3.0)	12.6 (8.6—17.4)	15.1 (10.8—20.2)	0.8 (0.1—3.0)
>60	221	33.0 (26.9—39.7)	9.5 (6.0—14.2)	0.5 (0.0—2.5)	1.4 (0.3—3.9)	9.0 (5.6—13.6)	20.8 (15.7—26.8)	5.4 (2.8—9.3)
**Sex**								
Male	431	15.8 (12.5—19.6)	5.8 (3.8—8.4)	0.5 (0.1—1.7)	1.4 (0.5—3.0)	4.2 (2.5—6.5)	10.2 (7.5—13.5)	0.9 (0.3—2.4)
Female	375	33.6 (28.8— 38.6)	7.7 (5.2—10.9)	1.3 (0.4—3.1)	1.9 (0.8— 3.8)	16.8 (13.2—21.0)	15.7 (12.2—19.8)	2.9 (1.5—5.2)
**Residence**								
Rural	457	17.5 (14.1—21.3)	9.0 (6.5—12.0)	1.1 (0.4—2.5)	1.8 (0.8—3.4)	2.2 (1.1— 4.0)	13.8 (10.8—17.3)	1.8 (0.8—3.4)
Urban	349	32.7 (27.8—37.9)	3.7 (2.0—6.3)	0.6 (0.1— 2.1)	1.4 (0.5—3.3)	20.3 (16.2—25.0)	11.5 (8.3—15.3)	2.0 (0.8—4.1)
**Wealth quintiles**								
poorest	108	32.4 (16.2—30.2)	13.0 (7.3—20.8)	0.9 (0.02—5.1)	0.9 (0.02—5.1)	7.5 (2.6—12.9)	25 (17.2—34.3)	5.6 (2.1—11.7)

For peer review only - http://bmjopen.bmj.com/site/about/guidelines.xhtml

second	150	22.7 (16.2—30.2)	5.3 (2.3—10.2)	0.0 (0—2.4)	0.6 (0.02—3.7)	7.3 (3.7—12.7)	14.0 (8.9—20.6)	2.0 (0.4—5.7)
third	139	15.8 (10.2—23.0)	6.5 (3.0—11.9)	0.0 (0—2.6)	1.4 (0.2—5.1)	6.5 (3.0—11.9)	8.6 (4.5—14.6)	0.7 (0.0—3.9)
fourth	191	24.1 (18.2—30.8)	4.7 (2.2—8.8)	2.1 (0.6—5.3)	0.5 (0.0—2.9)	13.1 (8.7—18.7)	11.0 (6.9—16.3)	1.6 (0.3—4.5)
wealthiest	218	26.1 (20.4—32.5)	6.4 (3.6—10.5)	0.9 (0.1—3.3)	3.7 (1.6—7.1)	13.3 (9.1—18.5)	10.1 (6.4—15.9)	0.9 (0.1—3.3)
Marital status								
Single	209	34.0 (27.6—40.8)	10.0 (6.3—15.0)	1.0 (0.1—3.4)	1.9 (0.5—4.8)	12.4 (8.2—17.7)	18.7 (13.6—24.6)	3.8 (1.7—7.4)
married	587	20.6 (17.4—24.1)	5.5 (3.8—7.7)	0.8 (0.3—1.9)	1.5 (0.7—2.8)	9.2 (7.0—11.8)	10.7 (8.4—13.5)	1.2 (0.5—2.4)
Educational achievement								
0-5 years primary education	164	25 (18.6—32.3)	7.9 (4.3—13.2)	0 (0.0—2.2)	1.2 (0.1—4.3)	6.7 (3.4—11.7)	16.5 (11.1—23.0)	4.3 (1.7—8.6)
Standard 6-8	329	25.5 (20.9—30.6)	6.7 (4.2—9.9)	0.6 (0.1—2.2)	1.5 (0.5—3.5)	11.9 (8.6—15.8)	12.5 (9.1—16.5)	2.1 (0.9—4.3)
Secondary	230	21.7 (16.6—27.6)	6.1 (3.4—10.0)	1.7 (0.5—4.4)	2.6 (1.0—5.6)	8.2 (5.0—12.6)	12.2 (8.2—17.1)	0.4 (0.0—2.4)
Post-secondary	83	22.9 (14.4—33.4)	6.0 (2.0—13.5)	1.2 (0.0—6.5)	0.0 (0—4.3)	14.5 (7.7—23.9)	8.4 (3.5—16.6)	0.0 (0.0—4.3)
occupation								
homemaker	237	37.1 (31.0—43.6)	7.2 (4.2—11.2)	1.7 (0.5—4.3)	2.5 (0.9—5.4)	18.6 (13.8—24.1)	15.6 (11.2—20.9)	4.2 (2.0—7.6)
Farming/ fishing	296	15.5 (11.6—20.2)	9.1 (6.1—13.0)	0.3 (0.0—1.9)	1.0 (0.2—2.9)	1.7 (0.6—3.9)	12.8 (9.2—17.9)	1.7 (0.6—3.9)
employed	273	22.0 (17.2—27.4)	3.7 (1..8—6.6)	0.7 (0.0—2.6)	1.5 (0.4—3.7)	11.7 (8.2—16.1)	10.3 (6.9—14.5)	0.0 (0—1.3)
Indoor fire								
none	42	7.1 (1.5—19.5)	4.8 (0.6—16.2)	0.0 (0—8.4)	2.4 (0.0—12.6)	4.8 (0.6—16.2)	0.0 (0—8.4)	0.0 (0—8.4)
Fire usually lit Inside the house	39	30.8 (17.0—47.6)	7.7 (1.6—20.9)	0.0 (0—9.0)	2.6 (0.0—13.5)	17.9 (7.5—33.5)	12.8 (4.3—27.4)	0.0 (0—9.0)
Fire usually lit in a separate	725	24.7 (21.6—28.0)	6.8 (5.0—8.8)	1.0 (0.4—2.0)	1.5 (0.8—2.7)	9.9 (7.9—12.3)	13.5 (11.1—16.2)	2.1 (1.2—3.4)

kitchen area								
**Any Smoking history**								
no	487	29.4 (25.4—33.6)	7.6 (5.4—10.3)	1.2 (0.5—2.7)	1.8 (0.8—3.5)	14.0 (11.0—17.4)	14.4 (11.4—17.8)	2.1 (1.0—3.7)
yes	319	16.0 (12.1—20.5)	5.3 (3.1—8.4)	0.3 (0.0—1.7)	1.3 (0.3—3.2)	4.1 (2.2—6.9)	10.3 (7.2—14.2)	1.6 (0.5—3.6)
**Diabetes**								
Non-diabetic	502	19.7 (16.3—23.5)	6.2 (4.2—8.7)	0 (0—0.7)	1.2 (0.4—2.6)	8.2 (5.9—10.9)	10.2 (7.7—13.1)	1.4 (0.6—2.9)
diabetic	273	33.7 (28.1—39.6)	7.7 (4.8—11.5)	2.6 (1.0—5.2)	2.2 (0.8—4.7)	13.9 (10.0—18.6)	18.7 (14.2—23.8)	2.9 (1.3—5.7)
Missing data	31	9.7 (2.0—25.8)	6.5 (0.8—21.4)	0.0 (0—11.2)	3.2 (0.0–16.7)	6.5 (0.8—21.4)	3.2 (0.1—16.7)	0.0 (0.0—11.2)
**Dyslipidaemia**								
no	413	22.8 (18.8—27.1)	6.5 (4.4—9.4)	0.7 (0.2—2.1)	1.2 (0.4—2.8)	10.4 (7.6—13.8)	10.7 (7.8—14.0)	2.4 (1.2—4.4)
yes	344	26.5 (21.9—31.5)	7.0 (4.5—10.2)	1.2 (0.3—3.0)	1.7 (0.6—3.8)	10.2 (7.2—13.9)	15.7 (12.0—20.0)	1.5 (0.5—3.4)
Missing data	49	18.4 (8.8—32.0)	6.1 (1.3—16.9)	0.0 (0.0—7.3)	4.1 (0.5—14.0)	6.1 (1.3—16.9)	10.2 (3.4—22.2)	0.0 (0.0—7.3)
**HIV**								
negative	413	23.0 (19.0—27.4)	7.5 (5.2—10.5)	0.7 (0.2—2.1)	1.2 (0.4—2.8)	10.0 (7.2—13.2)	12.1 (9.1—15.6)	2.4 (1.2—4.4)
positive	247	23.9 (18.7—29.7)	6.1 (3.4—9.8)	0.4 (0.0—2.2)	2.0 (0.7—4.7)	10.1 (6.7—14.6)	12.6 (8.7—`17.3)	0.8 (0.1—2.9)
unknown	146	27.4 (20.3—35.4)	5.5 (2.4—10.5)	0.0 (0—2.5)	0.7 (0.0—3.8)	10.3 (5.9—16.4)	15.1 (9.7—21.9)	2.7 (0.8—6.9)
**hypertension**								
Not-hypertensive	324	21.9 (17.6—26.8)	4.6 (2.6—7.5)	0.9 (0.2—2.7)	2.2 (0.9—4.4)	9.3 (6.3—13.0)	12.7 (9.2—16.8)	0.6 (0.1—2.2)
hypertensive	482	25.5 (21.7—29.7)	8.1 (5.8—10.9)	0.8 (0.2—2.1)	1.2 (0.5—2.7)	10.6 (8.0—13.7)	12.9 (10.0—16.2)	2.7 (1.4—4.6)
**obesity**								

For peer review only

no	634	20.8 (17.7—24.2)	5.8 (4.1—8.0)	0.6 (0.2—1.6)	1.6 (0.8—2.9)	7.3 (5.4—9.6)	11.8 (9.4—14.6)	1.9 (1.0—3.3)
yes	172	36.0 (28.9—43.7)	9.9 (5.9—15.4)	1.7 (0.4—5.0)	1.7 (0.4—5.0)	20.3 (14.6—27.1)	16.3 (11.1—22.7)	1.7 (0.4—5.0)
Number of risk factors								
2	201	14.9 (10.3—20.6)	3.0 (1.1—6.4)	1.0 (0.1—3.5)	1.1 (0.1–3.5)	7.5 (4.2—12.0)	6.5 (3.4—10.8)	0.5 (0.0—2.7)
3	342	23.1 (18.7—27.9)	6.7 (4.3—9.9)	0.3 (0.0—1.6)	2.0 (0.8—4.2)	8.8 (6.0—12.3)	12.0 (8.7—15.9)	2.6 (1.2—4.9)
4	204	32.4 (26.0 —39.2)	9.3 (5.7—14.2)	0.5 (0.0—2.7)	1.0 (0.5—4.9)	14.7 (10.1—20.3)	18.1 (13.1—24.1)	2.0 (0.5—4.9)
≥5	59	32.2 (20.6—45.6)	10.2 (3.8—20.8)	5.1 (1.1—14.1)	0.0 (0.0—6.1)	10.2 (3.8—20.8)	20.3 (11.0—32.8)	1.7 (0.0—9.1)
Prevalence of outcomes by risk factors								
No diabetes and no hypertension	193	14.0 (25.6—42.4)	4.1 (1.8—8.0)	0.0 (0.0—1.9)	1.6 (0.3—4.4)	4.7 (2.2—8.7)	9.3 (5.6—14.3)	0.0 (0.0—1.9)
Diabetes but no hypertension	131	33.6 (25.6—42.4)	5.3 (2.2—10.7)	2.3 (0.5 –6.5)	3.1 (0.8—7.6)	16.0 (10.2—23.5)	17.6 (11.5—25.2)	1.5 (0.2—5.4)
Hypertension but no diabetes	340	22.1 (17.8—26.8)	7.4 (4.8—10.7)	0.0 (0.0 —1.1)	1.2 (0.3—3.0)	10 (7.0—13.7)	10.0 (7.0—13.7)	2.1 (0.8—4.2)
Hypertension and diabetes	142	33.8 (26.1—42.2)	9.9 (5.5—16.0)	2.8 (0.8 —7.1)	1.4 (0.2 —5.0)	12.0 (7.1—18.5)	19.7 (13.5—27.2)	4.2 (1.6—9.0)

For peer review only

Table 2: full regression model of factors associated with Lower extremity disease

	Odds Ratio	Std. Err.	z	P>z	95% confidence interval	Lr test p value
**Age**						
<50	1	ref.				0.0012
50-60	1.91	0.4	2.8	0.0	1.22—2.99	
>60	2.33	0.6	3.4	0.0	1.43—3.8	
**sex**						
Male	1.0	ref.				0.0574
Female	1.69	0.5	1.9	0.1	0.98—2.9	
**site**						
Karonga	1.0	ref.				0.0296
Lilongwe	1.76	0.5	2.2	0.0	1.05—2.94	
**Wealth quintiles**						
poorest	1.93	0.7	1.9	0.1	0.99—3.77	0.3322
second	1.72	0.6	1.7	0.1	0.91—3.27	
third	1.0	ref.				
fourth	1.43	0.4	1.2	0.2	0.78—2.66	
wealthiest	1.61	0.5	1.5	0.1	0.85—3.05	
**Marital status**						
single	1.0	ref.				0.0862
married	0.69	0.1	-1.7	0.1	0.45—1.05	
**Highest attained education**						
years primary education	0.75	0.2	-1.2	0.2	0.46—1.22	0.2630
standard 6-8	1.0	ref.				
secondary	0.78	0.2	-1.1	0.3	0.49—1.22	
post-secondary	0.54	0.2	-1.7	0.1	0.27—1.08	
**Occupation**						
homemaker	1.62	0.5	1.7	0.1	0.93—2.83	0.2026
farming/fishing	1.0	ref.				
employed	1.22	0.3	0.7	0.5	0.7—2.13	
**Indoor smoke**						
No	0.19	0.1	-2.5	0.0	0.05—0.69	0.0105
Yes, inside the house	0.88	0.3	-0.3	0.7	0.4—1.91	
Yes, in a separate kitchen area	1.0	ref.				
**any history of smoking**						
no	1.0	ref.				0.8995
yes	0.96	0.3	-0.1	0.9	0.5—1.84	
**Diabetes**						
No	1.0	ref.				0.3532
Yes	0.95	0.3	-0.2	0.9	0.48—1.86	

For peer review only

Unknown	0.3	0.3	-1.4	0.2	0.06—1.61	
**Dyslipidaemia**						
No	1.0	ref.				0.8425
Yes	0.82	0.3	-0.5	0.6	0.41—1.66	
missing data	0.87	0.5	-0.3	0.8	0.28—2.67	
**HIV status**						
Negative	1.0	ref.				0.4490
Positive	0.98	0.3	-0.1	0.9	0.51—1.88	
Unknown	1.35	0.3	1.2	0.2	0.83—2.2	
**hypertension**						
No	1.0	ref.				0.1768
Yes	0.65	0.2	-1.3	0.2	0.35—1.22	
**obesity**						
No	1.0	ref.				0.9754
Yes	0.99	0.4	0.0	1.0	0.49—2.0	
**CVDRF**						
2	0.62	0.2	-1.3	0.2	0.3—1.3	0.4812
3	1.0	ref.				
4	1.49	0.5	1.1	0.3	0.72—3.07	
≥5	1.54	1.1	0.6	0.5	0.4—5.98	

For peer review only

Stratified analysis for lower extremity disease

Table 3: stratification by sex

	Male	Female
**Age**		
<50	(ref)	(ref)
50-60	1.96 (0.91—4.22)	1.97 (1.08—3.6)
>60	2.41 (1.08—5.37)	2 (1.0[*]—4.01)
**site**		
Karonga	(ref)	(ref)
Lilongwe	1.84 (0.79—4.31)	1.65 (0.82—3.34)
**Wealth quintiles**		
poorest	1.92 (0.6—6.19)	2.04 (0.86—4.88)
second	2.32 (0.87—6.18)	1.4 (0.58—3.39)
third	(ref)	(ref)
fourth	1.74 (0.65—4.64)	1.51 (0.66—3.46)
wealthiest	1.23 (0.42—3.6)	2.03 (0.87—4.72)
**Marital status**		
single	(ref)	(ref)
married	0.39 (0.18—0.86)	0.81 (0.47—1.39)
**Highest attained education**		
0-5 years prima..	1.01 (0.41—2.48)	0.69 (0.36—1.3)
standard 6-8	(ref)	(ref)
secondary	0.86 (0.42—1.73)	0.68 (0.36—1.28)
post-secondary	0.62 (0.19—2.06)	0.43 (0.17—1.09)
**Occupation**		
homemaker	1.09 (0.44—2.72)	2.19 (0.98—4.89)
farming/fishing	(ref)	(ref)
employed	0.91 (0.4—2.06)	1.87 (0.8—4.38)
**Indoor fire**		
No	(empty)	0.28 (0.07—1.17)
Yes, inside the house	0.66 (0.17—2.59)	1.06 (0.39—2.92)
Yes, but in a separate kitchen area.	(ref)	(ref)
**History of smoking**		
no	(ref)	(ref)
yes	0.79 (0.34—1.8)	1.83 (0.52—6.47)
**Diabetes**		
No	(ref)	(ref)
Yes	1.89 (0.71—5.01)	0.38 (0.13—1.18)
Unknown	0.48 (0.05—5.04)	0.18 (0.01—2.69)
**Dyslipidaemia**		

[*] Rounded up from .9989416

For peer review only

For peer review only - http://bmjopen.bmj.com/site/about/guidelines.xhtml

No	(ref)	(ref)
Yes	0.91 (0.32—2.59)	0.48 (0.16—1.49)
missing data	1.05 (0.18—6.21)	0.6 (0.12—2.97)
HIV status		
Negative	(ref)	(ref)
Positive	1.81 (0.74—4.41)	0.38 (0.13—1.13)
Unknown	1.69 (0.8—3.58)	0.93 (0.46—1.85)
hypertension		
No	(ref)	(ref)
Yes	0.52 (0.22—1.24)	0.39 (0.13—1.19)
obesity		
No	(ref)	(ref)
Yes	2.13 (0.64—7.07)	0.5 (0.17—1.48)
CVDRF		
2	0.68 (0.23—1.97)	0.45 (0.14—1.47)
3	(ref)	(ref)
4	1.37 (0.48—3.94)	2.59 (0.82—8.2)
≥5	0.76 (0.09—6.61)	6.48 (0.73—57.42)

For peer review only

Table 4: Stratification by Sex and history of smoking

Variables and variable categories	Male non-smoker	Male smoker	Female non-smoker	Female current smokers[†]
**Age**				
<50	(ref)	(ref)	(ref)	
50-60	2.74 (0.5—14.91)	1.43 (0.53—3.85)	2.1 (1.15—3.85)	
>60	4.53 (0.62—32.88)	1.86 (0.68—5.06)	1.95 (0.96—3.94)	
**Site**				
Karonga	(ref)	(ref)	(ref)	
Lilongwe	0.63 (0.12—3.41)	3.38 (1.07—10.62)	1.82 (0.88—3.78)	
**Wealth Quintile**				
poorest	6.26 (0.26—150.93)	1.58 (0.35—7.09)	1.71 (0.7—4.18)	
second	4.03 (0.18—89)	2.85 (0.9—9.05)	1.38 (0.56—3.43)	
third	(ref)	(ref)	(ref)	
fourth	4.67 (0.28—76.66)	1.27 (0.41—3.99)	1.24 (0.54—2.87)	
wealthiest	14.33 (0.91—225.09)	0.72 (0.16—3.21)	1.62 (0.69—3.81)	
**Marital status**				
single	(ref)	(ref)	(ref)	
married	0.04 (0.0—0.56)	0.58 (0.21—1.58)	0.91 (0.52—1.58)	
**Education**				
years primary education	4.93 (0.17—139.91)	0.88 (0.33—2.35)	0.64 (0.33—1.24)	
standard 6-8	(ref)	(ref)	(ref)	
secondary	3.02 (0.57—15.97)	0.62 (0.24—1.57)	0.78 (0.41—1.49)	
post-secondary	1.35 (0.19—9.71)	0.75 (0.07—8.19)	0.47 (0.19—1.18)	
**Occupation**				
homemaker	1.82 (0.29—11.26)	0.72 (0.2—2.6)	2.09 (0.9—4.88)	
farming/fishing	(ref)	(ref)	(ref)	
employed	0.43 (0.07—2.8)	1.1 (0.38—3.23)	1.68 (0.69—4.08)	
**Indoor smoke**				
No	(empty)	(empty)	(empty)	
Yes, inside the house	(empty)	0.82 (0.17—3.98)	1.02 (0.37—2.8)	
a separate kitchen area	(ref)	(ref)	(ref)	
**diabetes**				
No	(ref)	(ref)	(ref)	
Yes	1.57 (0.11—22.08)	3.79 (0.94—15.32)	0.35 (0.1—1.22)	
Unknown	(empty)	0.58 (0.02—16.66)	0.24 (0.02—3.85)	
**Dyslipidaemia**				
No	(ref)	(ref)	(ref)	
Yes	0.17 (0.01—3.9)	0.79 (0.2—3.14)	0.45 (0.13—1.53)	
missing data	0.28 (0.01—5.91)	0.7 (0.03—14.21)	0.57 (0.1—3.22)	
**HIV status**				
Negative	(ref)	(ref)	(ref)	

[†] No observations

Positive	1.82 (0.12—28.15)	2 (0.65—6.15)	0.41 (0.13—1.33)	
Unknown	2.01 (0.41—9.92)	1.74 (0.65—4.71)	0.99 (0.48—2.02)	
**hypertension**				
No	(ref)	(ref)	(ref)	
Yes	0.65 (0.06—6.93)	0.35 (0.1—1.15)	0.37 (0.11—1.25)	
**obesity**				
No	(ref)	(ref)	(ref)	
Yes	0.59 (0.04—9.2)	6.62 (1.06—41.46)	0.52 (0.16—1.72)	
**CVDRF**				
2	0.66 (0.04—11.7)	0.46 (0.11—1.85)	0.41 (0.11—1.49)	
3	(ref)	(ref)	(ref)	
4	20.29 (0.76—539.61)	0.51 (0.11—2.41)	2.67 (0.76—9.37)	
5	62.07 (0.17—22604.06)	0.26 (0.01—7.99)	6.71 (0.61—73.19)	

For peer review only

Table 5: Stratification by obesity and wealth quintiles

Variables and variable categories	non obese— Wealth quintile 1*	Non obese—wealth quintile 2	Non obese—wealth quintile 3	Non obese—wealth quintile 4	Non obese wealth quintile 5	Obese WQ1*‡	Obese WQ2*§	Obese—wealth quintile 3	Obese—wealth quintile 4	Obese—wealth quintile 5
**Age**										
<50	(ref)	(ref)	(ref)	(ref)	(ref)			1.45E-31 (.—.)	(ref)	(ref)
50-60	43.49 (1.51—1248.98)	4.57 (1.07—19.58)	0.8 (0.11—6.03)	2.63 (0.77—8.93)	0.85 (0.23—3.06)			5.54E-47 (.—.)	1.44 (0.07—30.58)	3.48 (0.5—24.14)
>60	12.11 (0.89—164.53)	4.43 (1.03—19.09)	0.42 (0.04—4.69)	3.82 (1.03—14.23)	2.18 (0.49—9.74)				1.91 (0.06—57.17)	2.65 (0.33—21.42)
**Sex**										
Male	(ref)	(ref)	(ref)	(ref)	(ref)			(ref)	(empty)	(ref)
Female	3.29 (0.44—24.85)	4.32 (0.64—28.94)	0.62 (0.03—13.88)	1.26 (0.32—4.93)	1.67 (0.45—6.18)			8.6E+107 (.—.)	(omitted)	1.9 (0.19—18.76)
**Site**										
Karonga	(ref)	(ref)	(ref)	(ref)	(ref)			(ref)	(empty)	(ref)
Lilongwe	12.72 (0.89—182.11)	3.95 (0.65—24.04)	2.64 (0.35—19.71)	2.49 (0.73—8.5)	0.53 (0.11—2.43)			2.6E+15 (.—.)	(omitted)	5.05 (0.13—202.03)
**Marital status**										
Single	(ref)	(ref)	(ref)	(ref)	(ref)			(ref)	(ref)	(ref)
married	1.39 (0.29—6.64)	0.95 (0.28—3.28)	0.29 (0.04—2.15)	0.5 (0.14—1.7)	0.64 (0.14—2.9)			8.6E+107 (.—.)	0.53 (0.03—8.15)	0.14 (0.02—1.04)
**Highest attained education**										
0—5 years primary education	11.09 (1.5—82.05)	0.89 (0.28—2.81)	0.34 (0.02—4.67)	0.76 (0.15—3.75)	2.09 (0.21—21.31)			8.1E-78 (.—.)	0.26 (0.0—16.1)	0.3 (0.02—5.47)
standard 6-8	(ref)	(ref)	(ref)	(ref)	(ref)			(ref)	(ref)	(ref)
secondary	57.88 (1.61—2080.34)	1.4 (0.3—6.59)	1.42 (0.27—7.35)	0.71 (0.24—2.11)	2.24 (0.63—7.94)			(empty)	0.34 (0.01—8.42)	0.49 (0.07—3.34)

* WQ= Wealth Quintile; the number at the end of the abbreviation indicates the stratum of interest

‡ No observations

§ No observations

11

For peer review only

post-secondary	42.93 (0.52—3528.5)	(empty)	(empty)	1.37 (0.08—24.48)	1.35 (0.27—6.7)				(empty)	0.37 (0.06—2.51)
**occupation**										
homemaker	0.8 (0.11—5.7)	0.61 (0.12—3.13)	21.78 (0.94—504.37)	0.79 (0.15—4.1)	5.97 (0.83—42.82)			4.7E+61 (.—.)	2.00 (0.04—100.23)	0.73 (0.0—155.92)
farming/fishing	(ref)	(ref)	(ref)	(ref)	(ref)			(ref)	(empty)	(ref)
employed	2.15 (0.29—16.04)	1.43 (0.23—9.04)	13.15 (0.9—192.03)	0.66 (0.17—2.58)	1.33 (0.23—7.83)			1.5E+154 (.—.)	(omitted)	0.19 (0.0—34.71)
**Indoor smoke**										
No	(empty)	0.26 (0.02—3.55)	(empty)	(empty)	(empty)				(empty)	0.75 (0.04—15.21)
Yes, inside the house	(empty)	2.91 (0.27—30.92)	(empty)	3.89 (0.65—23.19)	0.12 (0.01—1.5)				(empty)	1.64 (0.18—15.18)
a separate kitchen area	(ref)	(ref)	(ref)	(ref)	(ref)			(ref)	(ref)	(ref)
**History of Smoke**										
no	(ref)	(ref)	(ref)	(ref)	(ref)			(ref)	(ref)	(ref)
yes	0.29 (0.0—17.25)	3.07 (0.33—28.35)	0.12 (0.0—4.39)	0.94 (0.17—5.11)	0.26 (0.04—1.65)			4.1E+169 (.—.)	(empty)	0.54 (0.01—23.36)
**Diabetes**										
No	(ref)	(ref)	(ref)	(ref)	(ref)			(ref)	(ref)	(ref)
Yes	0.13 (0—16.29)	2.43 (0.29—20.13)	0.17 (0.0—9.5)	2.54 (0.54—12)	1.15 (0.19—7.16)			1.8E+46 (.—.)	0.07 (0.0—14.48)	1.15 (0.09—14.87)
Unknown	(empty)	(empty)	2.1E-08 (0.1—22.1)	7.2E+05 (0.0—.)	(empty)				(empty)	
**Dyslipidaemia**										
No	(ref)	(ref)	(ref)	(ref)	(ref)			(ref)	(ref)	(ref)
Yes	0.06 (0.0—4.64)	0.58 (0.07—4.84)	0.06 (0.0—1.92)	3.03 (0.53—17.14)	0.06 (0.0—1.19)			2.6E+07 (.—.)	0.31 (0.0—22.6)	0.93 (0.03—26.3)
missing data	(empty)	(empty)	7.7E+07 (0.0—.)	2.0E-06 (0.0—.)	0.18 (0.02—2.11)			(empty)	(empty)	0.48 (0—64.61)
**HIV status**										
Negative	(ref)	(ref)	(ref)	(ref)	(ref)			(ref)	(ref)	(ref)
Positive	0.08 (0.0—6.74)	1.15 (0.17—7.63)	0.12 (0.0—4.26)	1.94 (0.37—10.03)	1.41 (0.24—8.31)			1.45E-31 (.—.)	0.24 (0—22.56)	1.98 (0.14—27.98)
Unknown	2.1 (0.33—13.38)	1.66 (0.4—6.81)	1.33 (0.08—22.1)	2.04 (0.5—8.3)	1.63 (0.27—9.77)			1.45E-31 (.—.)	0.39 (0.02—7.7)	1.79 (0.23—13.66)

For peer review only

**Hypertension**										
No	(ref)	(ref)	(ref)	(ref)	(ref)			(ref)	(ref)	(ref)
Yes	0.11 (0.0—9.9)	0.7 (0.1—4.87)	0.03 (0.0—0.91)	0.85 (0.21—3.47)	0.6 (0.09—4.13)			1.81E+46 (.—.)	0.33 (0.01—19.43)	1.41 (0.14—14.27)
**CVDRF**										
2	0.03 (0.0—1.88)	0.8 (0.1—6.22)	0.0 (0.0—0.29)	1.59 (0.28—9.08)	1.29 (0.17—9.96)					
3	(ref)	(ref)	(ref)	(ref)	(ref)			(ref)	(ref)	(ref)
4	49.06 (0.47—5153.28)	1.87 (0.18—18.98)	2.7 (0.06—112.27)	0.92 (0.18—4.78)	16.97 (1.14—253.16)			1.81E+46 (.—.)	1.17 (0.0—154.19	0.58 (0.02—18.29)
5	(empty)	(empty)	(empty)	(empty)	(empty)			(omitted)	6.75 (0.0—24323.16)	0.48 (0.0—105.76)

For peer review only

Table 6: Stratification by highest attained education and obesity

Variables and variable categories	Non-obese & 0-5 years elementary education	Non-obese & 6-8 elementary education	Non obese & secondary education	Non-obese & post-secondary education[**]	Obese & 0-5 years elementary education[††]	Obese & 6-8 years elementary education	Obese & secondary education	Obese & post-secondary education[‡‡§§]
**Age**								
<50	(ref)	(ref)	(ref)	(ref)	(ref)	(ref)	(ref)	(ref)
50-60	10.76 (1.45—79.81)	1.33 (0.57—3.15)	2.18 (0.66—7.22)	.	2.07e+19 (.—.)	2.96 (0.6—14.49)	12.52 (0.3—525.67)	1.14E-57 (.—.)
>60	5.85 (0.9—38)	1.65 (0.63—4.29)	6.74 (1.83—24.92)	.	264.2 (.—.)	3.12 (0.43—22.52)	5.03 (0.02—1686.17)	1.64E+56 (.—.)
**Sex**								
Male	(ref)	(ref)	(ref)	(ref)	(omitted)	(ref)	(ref)	(ref)
Female	7.38 (0.9—60.6)	2.73 (0.77—9.71)	1.28 (0.35—4.69)	.		0.47 (0.03—8.69)	0.84 (0.02—45.4)	4.96E+56 (.—.)
**Site**								
Karonga	(ref)	(ref)	(ref)	(empty)	(ref)	(ref)	(ref)	(ref)
Lilongwe	5.38 (0.91—31.62)	3.06 (1.12—8.35)	0.88 (0.26—3.04)	(omitted)	3.6E+22 (.—.)	2.01 (0.23—17.74)	1.06 (0.01—97.43)	3.8E+107 (.—.)
**Wealth quintiles**								
poorest	25.05 (1.9—330.22)	1.59 (0.42—6.0)	1.72 (0.23—12.74)	.	1.8E+21 (.—.)	0.3 (0.03—3.47)	(empty)	(empty)
second	7.82 (0.71—86.4)	3.3 (1.02—10.68)	1.56 (0.28—8.67)		3.1E+42 (.—.)	0.37 (0.03—4.01)	13.09 (0.03—5949.07)	(empty)
third	(ref)	(ref)	(ref)	(empty)	(ref)	(ref)	(ref)	(empty)
fourth	2.4 (0.15—37.21)	1.85 (0.64—5.37)	1.96 (0.49—7.89)	(empty)	3.9E-21 (.—.)	0.22 (0.02—2.3)	0.24 (0—38.03)	(empty)
wealthiest	2.09 (0.1—42.23)	0.84 (0.24—2.92)	3.39 (0.75—15.3)	(omitted)	7.8 (.—.)	0.44 (0.04—4.71)	11.69 (0.09—1444.24)	(omitted)
**Marital status**								
single	(ref)	(ref)	(ref)	(ref)	(ref)	(ref)	(ref)	(ref)

[**] Site, wealth quintiles and occupation omitted because of collinearity
[††] Sex, occupation and history of smoking omitted because of collinearity
[‡‡] Wealth and occupation have no observations
[§§] History of smoking and CVDRF omitted because of collinearity

For peer review only - http://bmjopen.bmj.com/site/about/guidelines.xhtml

married	1.11 (0.28—4.43)	1.32 (0.54—3.23)	0.31 (0.11—0.91)	**0.0 (.—.)**	4.6E-22 (.—.)	0.69 (0.11—4.38)	0.13 (0.01—2.61)	7.38E+17
occupation								
homemaker	2.51 (0.56—11.17)	1.66 (0.54—5.08)	0.69 (0.17—2.84)	**0.0 (.—.)**	4.0 e+19 (.—.)	2.78 (0.29—26.69)	0.3 (0.01—12.69)	1.04E+95 (.—.)
farming/fishing	(ref)	(ref)	(ref)	(empty)	(empty)	(ref)	(empty)	(empty)
employed	1.87 (0.27—12.76)	1.83 (0.67—4.99)	0.5 (0.12—2.03)	**1 (omitted)**	1 **(omitted)**	1.71 (0.15—19.93)	1 (omitted)	(omitted)
Indoor smoke								
No	0.96 (0.04—21.49)	(Empty)	(Empty)	(empty)	(empty)	(empty)	(empty)	4.62E+74 (.—.)
Yes, inside the house	1.93 (0.21—17.91)	1.88 (0.42—8.4)	0.11 (0.01—1.59)	(empty)		0.93 (0.05—18.44)	5.99 (0.04—988.69)	4.72E+94 (.—.)
Yes, in a separate kitchen area	(ref)	(ref)	(ref)	(ref)	(ref)	(ref)	(ref)	(ref)
Smoking history								
no	(ref)	(ref)	(ref)	(ref)	(ref)	(ref)	(ref)	(ref)
yes	10.25 (0.96—109.45)	1.29 (0.29—5.66)	0.25 (0.05—1.3)	**0.0 (.—.)**	(empty)	0.35 (0.01—14.51)	9.43E+07 (0.0—.)	(empty)
Diabetes								
No	(ref)	(ref)	(ref)	(ref)	(ref)	(ref)	(ref)	(ref)
Yes	1.06 (0.1—11.91)	0.88 (0.21—3.71)	1.28 (0.26—6.33)	**0.0 (.—.)**	1.2E-20 (.—.)	0.53 (0.05—5.5)	1.4e+07 (0.0—.)	2.0E+163 (.—.)
Unknown	(empty)	0.22 (0.01—5.99)	(empty)	(empty)		(empty)	(empty)	
Dyslipidaemia								
No	(ref)	(ref)	(ref)	(ref)	(ref)	(ref)	(ref)	(ref)
Yes	1.05 (0.12—9.23)	0.43 (0.11—1.75)	2.43 (0.38—15.56)	**0.0 (.—.)**	1.4E+23 (.—.)	1 (0.05—21.22)	32.63 (0.17—6363.24)	1.5E-127 (.—.)
missing data	0.41 (0.02—7.53)	3.39 (0.18—65.18)	0.14 (0.01—2.05)	(empty)		(empty)	109.64 (0.06—205197.3)	(empty)
HIV status								
Negative	(ref)	(ref)	(ref)	(ref)	(ref)	(ref)	(ref)	(ref)
Positive	0.85 (0.08—9.02)	0.6 (0.14—2.55)	2.5 (0.62—10.07)	.	5.5E+23 (.—.)	0.88 (0.05—17.25)	24.16 (0.59—992.53)	1.6E+105 (.—.)

For peer review only

For peer review only - http://bmjopen.bmj.com/site/about/guidelines.xhtml

Unknown	0.87 (0.15—5.16)	1.34 (0.52—3.45)	2.37 (0.6—9.4)	.	(empty)	0.75 (0.14—3.89)	157.01 (0.5—49479.48)	8.25E-77 (.—.)
**Hypertension**								
No	(ref)	(ref)	(ref)	(ref)	(ref)	(ref)	(ref)	(ref)
Yes	1.58 (0.23—10.99)	0.51 (0.13—1.97)	0.68 (0.13—3.57)	0.0 (.—.)	4.4E-38 (.—.)	0.44 (0.04—5.13)	2.37 (0.06—95.27)	6.1E+132 (.—.)
**CVDRF**								
2	0.53 (0.05—5.26)	0.18 (0.04—0.8)	2.39 (0.45—12.74)	.				(ref)
3	(ref)	(ref)	(ref)	(ref)	(ref)	(ref)	(ref)	2.43E+94 (.—.)
4	2.88 (0.28—29.86)	2.42 (0.57—10.31)	0.62 (0.1—3.8)	.	1.4E-22 (.—.)	0.99 (0.04—23.46)	27.92 (0.04—17774.42)	(omitted)
≥5		(empty)	21.02 (0.37—1193.6)	(empty)	4.4e-07 (.—.)	1.95 (0.01—423.84)	1.44 (0.0—2718.99)	1.14E-57 (.—.)

For peer review only

STROBE Statement—Checklist of items that should be included in reports of ***cross-sectional studies***

	Item No	Recommendation	Page No
**Title and abstract**	1	(*a*) Indicate the study's design with a commonly used term in the title or the abstract	1
		(*b*) Provide in the abstract an informative and balanced summary of what was done and what was found	2
**Introduction**			
Background/rationale	2	Explain the scientific background and rationale for the investigation being reported	4
Objectives	3	State specific objectives, including any prespecified hypotheses	5
**Methods**			
Study design	4	Present key elements of study design early in the paper	5
Setting	5	Describe the setting, locations, and relevant dates, including periods of recruitment, exposure, follow-up, and data collection	5
Participants	6	(*a*) Give the eligibility criteria, and the sources and methods of selection of participants	5
Variables	7	Clearly define all outcomes, exposures, predictors, potential confounders, and effect modifiers. Give diagnostic criteria, if applicable	6-7
Data sources/ measurement	8*	For each variable of interest, give sources of data and details of methods of assessment (measurement). Describe comparability of assessment methods if there is more than one group	6
Bias	9	Describe any efforts to address potential sources of bias	11
Study size	10	Explain how the study size was arrived at	7
Quantitative variables	11	Explain how quantitative variables were handled in the analyses. If applicable, describe which groupings were chosen and why	7
Statistical methods	12	(*a*) Describe all statistical methods, including those used to control for confounding	7
		(*b*) Describe any methods used to examine subgroups and interactions	7
		(*c*) Explain how missing data were addressed	
		(*d*) If applicable, describe analytical methods taking account of sampling strategy	7
		(*e*) Describe any sensitivity analyses	
**Results**			
Participants	13*	(a) Report numbers of individuals at each stage of study—eg numbers potentially eligible, examined for eligibility, confirmed eligible, included in the study, completing follow-up, and analysed	8
		(b) Give reasons for non-participation at each stage	
		(c) Consider use of a flow diagram	16
Descriptive data	14*	(a) Give characteristics of study participants (eg demographic, clinical, social) and information on exposures and potential confounders	8
		(b) Indicate number of participants with missing data for each variable of interest	8
Outcome data	15*	Report numbers of outcome events or summary measures	8
Main results	16	(*a*) Give unadjusted estimates and, if applicable, confounder-adjusted estimates and their precision (eg, 95% confidence interval). Make clear which confounders were adjusted for and why they were included	9

For peer review only

		(*b*) Report category boundaries when continuous variables were categorized	
		(*c*) If relevant, consider translating estimates of relative risk into absolute risk for a meaningful time period	
Other analyses	17	Report other analyses done—eg analyses of subgroups and interactions, and sensitivity analyses	9
**Discussion**			
Key results	18	Summarise key results with reference to study objectives	9
Limitations	19	Discuss limitations of the study, taking into account sources of potential bias or imprecision. Discuss both direction and magnitude of any potential bias	2 & 12
Interpretation	20	Give a cautious overall interpretation of results considering objectives, limitations, multiplicity of analyses, results from similar studies, and other relevant evidence	10-12
Generalisability	21	Discuss the generalisability (external validity) of the study results	10-12
**Other information**			
Funding	22	Give the source of funding and the role of the funders for the present study and, if applicable, for the original study on which the present article is based	8

*Give information separately for exposed and unexposed groups.

Note: An Explanation and Elaboration article discusses each checklist item and gives methodological background and published examples of transparent reporting. The STROBE checklist is best used in conjunction with this article (freely available on the Web sites of PLoS Medicine at http://www.plosmedicine.org/, Annals of Internal Medicine at http://www.annals.org/, and Epidemiology at http://www.epidem.com/). Information on the STROBE Initiative is available at www.strobe-statement.org.

For peer review only

BMJ Open

The prevalence and risk factors of lower extremity disease in high risk groups in Malawi: a stratified cross-sectional study

Journal:	*BMJ Open*
Manuscript ID	bmjopen-2021-055501.R2
Article Type:	Original research
Date Submitted by the Author:	19-May-2022
Complete List of Authors:	Kasenda, Stephen; Malawi Epidemiology and Intervention Research Unit<br>Crampin, Amelia; Malawi Epidemiology and Intervention Research Unit; London School of Hygiene & Tropical Medicine<br>Davies, Justine ; King's College London, Global Health<br>Malava, Jullita; Malawi Epidemiology and Intervention Research Unit<br>Manganizithe, Stella; Malawi Epidemiology and Intervention Research Unit<br>Kumambala, Annie; Malawi Epidemiology and Intervention Research Unit<br>Sandford, Becky; Guy's and St Thomas' NHS Foundation Trust
**Primary Subject Heading**:	Epidemiology
Secondary Subject Heading:	Cardiovascular medicine
Keywords:	EPIDEMIOLOGY, CARDIOLOGY, Cardiac Epidemiology < CARDIOLOGY, Hypertension < CARDIOLOGY

SCHOLARONE™
Manuscripts

I, the Submitting Author has the right to grant and does grant on behalf of all authors of the Work (as defined in the below author licence), an exclusive licence and/or a non-exclusive licence for contributions from authors who are: i) UK Crown employees; ii) where BMJ has agreed a CC-BY licence shall apply, and/or iii) in accordance with the terms applicable for US Federal Government officers or employees acting as part of their official duties; on a worldwide, perpetual, irrevocable, royalty-free basis to BMJ Publishing Group Ltd ("BMJ") its licensees and where the relevant Journal is co-owned by BMJ to the co-owners of the Journal, to publish the Work in this journal and any other BMJ products and to exploit all rights, as set out in our licence.

The Submitting Author accepts and understands that any supply made under these terms is made by BMJ to the Submitting Author unless you are acting as an employee on behalf of your employer or a postgraduate student of an affiliated institution which is paying any applicable article publishing charge ("APC") for Open Access articles. Where the Submitting Author wishes to make the Work available on an Open Access basis (and intends to pay the relevant APC), the terms of reuse of such Open Access shall be governed by a Creative Commons licence – details of these licences and which Creative Commons licence will apply to this Work are set out in our licence referred to above.

Other than as permitted in any relevant BMJ Author's Self Archiving Policies, I confirm this Work has not been accepted for publication elsewhere, is not being considered for publication elsewhere and does not duplicate material already published. I confirm all authors consent to publication of this Work and authorise the granting of this licence.

For peer review only

The prevalence and risk factors of lower extremity disease in high risk groups in Malawi: a stratified cross-sectional study

Author and author affiliations

Stephen Kasenda[1], Amelia C. Crampin[1,2,3], Justine Davies[4,5,6*], Jullita Kenala Malava[1], Stella Manganizithe[1], Annie Kumambala[1], Becky Sandford[7]

Author affiliations

[1] Malawi Epidemiology and Intervention Research Unit, Lilongwe, Malawi

[2] London School of Hygiene & Tropical Medicine, London, UK

[3] University of Glasgow, Institute of Health and Wellbeing, Glasgow, UK

[4] Institute of Applied Health Research, University of Birmingham, UK

[5] Centre for Global Surgery, Department of Global Health, Stellenbosch University, Cape Town

[6] Medical Research Council/Wits University Rural Public Health and Health Transitions Research Unit, Faculty of Health Sciences, School of Public Health, University of the Witwatersrand, Johannesburg, South Africa

[7] Guy's and St Thomas' NHS Trust, London, UK

*Corresponding author

Author contact details and ORCID

SK: Stephen.kasenda@meiru.mw; 0000-0001-9208-4122

AC: mia.crampin@lshtm.ac.uk; 0000-0002-1513-4330

JD: J.Davies.6@bham.ac.uk; 0000-0001-6834-1838

JKM: jullita.malava@meiru.mw;

SM: stellamanganizithe@gmail.com

AK: anniekumambala@gmail.com

BS: Becky.Sandford@gstt.nhs.uk; 0000-0001-6337-936X

For peer review only

For peer review

Abstract

Objective: Low and middle income countries face a disproportionate burden of cardiovascular diseases. However, amongst cardiovascular diseases, burden of and associations with lower extremity disease (peripheral arterial disease and/or diabetic foot disease) is neglected. We investigated the prevalence and factors associated with lower extremity disease (LED) among individuals known to have cardiovascular risk factors in Malawi, a low income country with a significant prevalence of cardiovascular disease risk factors.

Design: This was a stratified cross-sectional study.

Setting: This study was conducted in urban Lilongwe area 25, and the rural Karonga Health and Demographic Surveillance Site.

Participants: Participants were at least 18 years old and had been identified to have two or more known risk factors for cardiovascular disease (CVDRF).

Main outcome measures: Lower extremity disease—determined by the presence of one of the following: neuropathy (as assessed by a 10g monofilament), arterial disease (absent

peripheral pulses, claudication as assessed by the Edinburgh claudication questionnaire, or ankle brachial pulse index <0.9), previous amputation or ulceration of the lower limbs.

Results: There were 806 individuals enrolled into the study. Mean age was 52.5 years; 53.5% of participants were male (n=431) and 56.7% (n=457) were from the rural site. Nearly a quarter (24.1%; 95%: 21.2—27.2) of the participants had at least one symptom or sign of LED. 12.8% had neuropathy, 6.7% had absent pulses, 10.0% had claudication, 1.9% had ABPI<0.9, 0.9% had an amputation, and 1.1% had lower limb ulcers. LED had statistically significant association with increasing age, urban residence, and use of indoor fires.

Conclusions: This study demonstrated that a quarter of individuals with two or more CVDRFs have evidence of LED and 2.4% have an amputation or signs of limb threatening ulceration or amputation. Further epidemiological and health systems research is warranted to prevent LED and limb loss.

Strengths and limitations of this study

- This study is the first attempt in Malawi to determine the prevalence of LED.
- This study used multiple parameters to ensure accurate estimation of the prevalence of LED.
- This study included both rural and urban populations to provide a comprehensive picture of LED prevalence.
- Some participants with vascular calcification may have had false negative ABPI results

Data availability statement: Data are available on reasonable request

Word count: 3000

Supplemental material: For detailed data on the distribution

Keywords: Malawi, peripheral artery disease, cardiovascular diseases, HIV, hypertension, diabetes mellitus, obesity

Funding: This work was supported by King's Health Partners and Development Challenge Fund (MC_PC_16048). The baseline survey was funded by the Wellcome Trust (098610/Z/12/Z and 098610/B/12/A). The funders had no role in the study design; collection, analysis, and interpretation of data; or report writing. The corresponding author had full access to the data and the final responsibility to submit for publication.

Competing interests: None declared.

Author contributions:

SK: data collection, analysis, drafting and revising manuscript,

For peer review only - http://bmjopen.bmj.com/site/about/guidelines.xhtml

AC: data collection, design, analysis and revising manuscript
JD: design, analysis and revising manuscript
JKM: data collection and revising manuscript
SM: data collection and revising manuscript
AK: data collection and revising manuscript
BS: design, analysis and revising manuscript

Introduction

Peripheral vascular disease and peripheral neuropathies (including the spectrum of diabetic foot disease) share a common group of overlapping risk factors, namely cardiovascular risk factors (CVDRFs) such as hypertension, diabetes, smoking, obesity, and hypercholesterolaemia. Infectious agents, particularly HIV also contribute to increased risk of peripheral vascular disease(1). Resources required to optimize preventative strategies, treat complications, and offer rehabilitation after surgery for peripheral vascular disease and peripheral neuropathies disease are largely the same whether the underlying disease process was predominantly ischaemic or neuropathic. As such, we have considered these conditions as one entity representing a spectrum of problems under the term 'Lower extremity disease" (LED). Individuals with LED are at risk of lower limb amputation, particularly in cases where there is significant ischaemic necrosis and infection. Amputation can be a life-saving procedure, relieving pain and sepsis, but may also be associated with negative physical, psychological, social, and economic consequences (2,3).

With increasing prevalence of CVDRF in lower and middle income countries (LMIC) superimposed on a background prevalence of chronic infections, it is strongly suspected that associated risk of lower limb amputation will become increasingly important for individuals, families, health systems, and economies(4). Evidence from LMICs is lacking, but suggestive that burden of amputations related to LED conditions will grow. Almost 70% of people with peripheral arterial disease live in LMICs; African countries in particular show a high population-prevalence of peripheral arterial disease (e.g. 33% in a population of people >65 years old in Central Africa)(5–7). Studies in people who have managed to access CVDRF clinics in referral hospitals also show a large number of people have peripheral artery disease; for example, in Ghana, 27% of nearly 1000 people with diabetes had clinically measurable peripheral arterial disease(8). In our pilot study of 191 patients attending a hospital clinic in Blantyre, Malawi, peripheral arterial disease was seen in 8.5% of 45-64 year olds and 17% of people aged 65 and over(9). Reliable data on population prevalence of diabetic foot ulceration are lacking, however, studies show that in people with

diabetes who have accessed services, prevalence ranged from 4.0 to 9.9%; it could be far higher if people who had not accessed services were included(10).

To develop health systems to manage and prevent LED and its consequences requires knowledge of its prevalence. The aim of this study was to investigate the population prevalence of LED amongst high risk individuals in rural and urban Malawi.

Methods

Study setting

The study was conducted in Area 25 in urban Lilongwe, and the rural Karonga Health and Demographic Surveillance Site (HDSS). The Karonga site has a population of approximately 45,000 and the economy is mainly subsistence farming. Area 25 (Lilongwe) has an approximate total population of 65,000 with a mixed economy.

Study design and participants

We conducted a stratified cross-sectional study of prevalence of "lower extremity disease" (LED) defined as neuropathy and/or vasculopathy in all adults of at least 18 years age with two or more cardiovascular disease risk factors. This population was selected to ensure a reasonable sample size of people at highest risk of lower extremity disease.

Identification of participants

We included all eligible participants who were at least 18 years old and had been identified to have two or more known risk factors for LED during a population wide survey of cardio-metabolic conditions conducted between 2013 and 2017 in the study areas (11). Presence of hypertension, diabetes mellitus, HIV, tobacco smoking, obesity, and age at least 40 years were used to characterise high risk individuals.

Data collection and definitions

Data on participant socio-demographic characteristics (sex, date of birth, marital status, highest attained education, use of indoor fire, and household wealth) and presence of CVDRFs (history of smoking, diabetes mellitus, hypertension, dyslipidaemia, and HIV status using previous laboratory diagnosis) were extracted from the initial baseline study. Methods used to capture data and definitions used in that baseline survey have been published

elsewhere (11). In brief, diabetes mellitus was defined as a fasting blood glucose of at least 7.0 mmol/L (as determined by a Beckman Coulter AU480 Chemistry Analyser) or self-report of a previous diagnosis of the condition by a health professional regardless of the drug history. Hypertension was defined as a blood pressure of at least 140/90 mmHg or current use of antihypertensive medication for blood pressure control. Participants with a body mass index (BMI) of at least 30 kg/m^2 were classified as having obesity. Participants were defined as smokers if they reported smoking at least one cigarette per day in the immediate past six months preceding the survey. Participants were defined as HIV-positive if they had previously been tested antibody positive by the study team or self-reported HIV-positivity.

For the current study, participants were interviewed and examined in their own homes and data collectors were blind to the participant's previous medical history. In cases where the potential participant was missed during the first visit and it was established that they had neither left the study area nor died, the household was visited at least three times before declaring the potential participant as "missed". All participants were interviewed to determine if they had any changes in cardiovascular disease risk profile. The Edinburgh claudication questionnaire was used to determine the presence of claudication and participants who reported the presence of clinical CVDRFs were asked additional questions to regarding access to care for the relevant conditions they have.

Participants were examined to determine the presence of leg or foot ulcers, palpable peripheral pulses (brachial, posterior tibial, and dorsalis pedis), and/ or lower limb amputations in both extremities. Neuropen® 10g monofilaments were used to test for presence of peripheral neuropathy on the plantar surfaces great toe as well as over the metatarsal head of the great toe, 3rd and 5th toes of both feet. Portable sphygmomanometers (Welch Allyn DS55 Durashock hand aneroid model) and handheld continuous wave Doppler ultrasound devices (Bistos Hi.dop BT-200 vascular Doppler with 8 MHZ probe) were used to determine Ankle Brachial Pressure Index (ABPI). ABPI for each participant was determined according to the methods recommended by Aboyans et. al(12,13). A low ABPI reading suggests peripheral arterial disease, with the blood pressure reduced at the ankle compared with the arm which implies stenosis within the arterial tree supplying the lower limbs. Each participant was considered to have low ABPI if they had ABPI less than 0.9 on any or both sides.

Outcomes

The primary outcome of interest was the presence of any symptom or sign suggestive of lower extremity disease, termed “LED prevalence” (history of claudication, limb ulcers, loss of sensation on the plantar surfaces of feet, or Ankle Brachial Pressure Index [ABPI] less than 0.9). Secondary outcomes were these as individual outcomes.

Statistical analysis

Given the dearth of information on the prevalence of LED in the local setting, we based our sample size calculation on pilot data from a study of 90 people with diabetes living in the Malawi HDSS, which suggested that the prevalence of LED was around 10%. Considering people with diabetes to be at high risk and similar to those chosen for our study, we calculated a sample of 912 people would be required to detect a prevalence of LED of 10% with a CI of 6-14%.

Prevalence of the primary and secondary outcomes, socio-demographic characteristics of the study participants, and CVDRFs are described as mean (SD) and count (%). Logistic regression and likelihood ratio tests were done to determine associations between the primary outcome and independent variables. Socio-demographic characteristics and presence of CVDRFs were considered as a priori independent variables of interest in our analysis. The association between number of CVDRFs and the crude prevalence of the outcome of interest was also investigated.

All variables of interest were added to the regression model and likelihood ratio testing was done to determine the presence of interactions between the independent variables. Stratified analyses of the full regression model were done for all variables that were identified as having statistically significant interactions. All statistical analysis was done using Stata 15 statistical package.

Role of the funding source

Funding for this study was provided by King’s Health Partners and Development Challenge Fund (MC_PC_16048), King’s College London, UK. The baseline survey was funded by the Wellcome Trust (098610/Z/12/Z and 098610/B/12/A). The funders had no role in the study design; collection, analysis, and interpretation of data; or report writing. The corresponding author had full access to the data and the final responsibility to submit for publication.

Ethical approvals

Ethical approval for the baseline survey was granted by the Malawi National Health Sciences Research Committee (NHRSC) protocol number #1072, and London School of Hygiene and

Tropical Medicine (LSHTM) Ethics Committee protocol number #6303. Ethical approval for the lower extremity disease study was granted by NHSRC approval number #2090 and King's College London BDM Research Ethics Panel (reference: LRS-17/18-7983). Data was collected from potential participants who gave written informed consent. Additional consent was sought from the participants for utilization of their previously obtained HIV results in data analysis for this study.

Patient and public involvement

There was no patient and public involvement in this study.

Results

A total of 829 people were visited, of whom 806 (97.2%) consented to be recruited (Figure 1). Most of the participants were at least 50 years age (mean age 52.5; 95%CI: 51.7—53.3), male (53.5%; n=431), from the rural site (56.7%; n=457), employed (70.6%; n=569), married (74.1%; n=597), and had no more than primary education (61.1%; n=493). Cooking with biomass fuel was reported by 94.8% (n=764) of the participants (Table 1). The majority 88.1% (n=710) of participants, had at least two clinical risk factors for LED (hypertension, obesity, HIV, diabetes, and dyslipidaemia), and the remaining 96 participants were aged at least 40 years and had a history of smoking tobacco (Figure 2 & Table 1). The mean age of the rural participants was higher than that of the urban participants (53.5 years [95%CI: 52.4—54.6] compared to 51.2 years [95%CI: 50.1—52.3]; P=0.005). Missing data accounted for not more than 6.1% of all the data on any variable.

Nearly a quarter (24.9%; 95%: 22.0—28.1) of the participants had at least one symptom or sign of LED (Table 2). Peripheral neuropathy (N=103; 12.8%) and claudication (N=81; 10 %) were the most common features of LED, and signs of limb threatening disease [i.e. ulcers (N=13; 1.1 %) and lower limb amputations (N=7; 0.9%)] were the least prevalent, affecting 2.4% (n=19) of participants. ABPI less than 0.9 was recorded among 32 (4.0%) participants, and 391 (48.5%) had ABPI greater than 1.2. (Appendix table 1) Four participants had ABPI less than 0.9 on one side & ABPI greater than 1.2 on the contralateral side. In view of the reduced ABPI <0.9, which is more likely to be a true positive, these individuals were categorised as having low ABPI. Those with ABPI >1.2 on both sides were grouped together

with participants with ABPI between 0.9 and 1.2. At least 25% of participants with each CVDRF, apart from smoking, had LED. LED prevalence rates of over 30% were recorded among participants who were urban residents, female, aged over 50 years, single, indoor users of biomass fuel, or homemakers. The prevalence of LED was directly proportional to the number of CVDRFs with the highest prevalence of LED [35.6%; 95%CI: 23.6—49.1] being recorded among participants that had five or more risk factors (table 2).

In the full regression model (figure 3), statistically significantly increased odds of LED were found in participants aged at least 50 years ((OR: 1.19; 95%CI: 1.22—2.99) among those aged 50-60, and OR: 2.33; 95%CI: 1.43—3.8 among those over 60 years age) and urban residents (OR: 1.76; 95%CI: 1.05—2.94) (Appendix table 2). Cooking with methods other than biomass burning was negatively associated with LED [OR: 0.19; 95%CI: 0.05—0.69]. The following sets of variables had some statistically significant interactions that affected the odds of the LED: (I) sex and history of smoking, (II) obesity and highest attained education, and (III) obesity and participant's wealth quintile, as computed in the full model. The results of these interactions are presented in Appendix tables 3- 6.

Discussion

The aim of this study was to describe the prevalence of lower extremity disease (LED) amongst individuals with known cardiovascular disease risk factors (CVDRF) in rural and urban Malawi. Of 806 individuals studied, a large proportion had at least one sign of LED, with claudication and peripheral neuropathy being the most common. Prevalence of LED was directly related to the number of risk factors with advancing age, urban living, and poverty demonstrating the greatest association. Although amputations and active ulceration were less frequently seen, 2.5% of individuals in this study had these conditions which are indicative of active or previous limb threatening disease.

Provision of health care services to manage cardiovascular risk factors or care for patients with LED are limited in Malawi and tend to be focused around large urban centres(14). Health literacy also tends to be poor amongst those living with chronic conditions, with patients and families often understanding little about their condition or the longer term consequences of inadequate management (15). Provision of surgical services for the management of LED (e.g. vascular reconstructive techniques, amputation and rehabilitation services) are limited throughout southern Africa and this field has not been viewed as a priority amongst competing healthcare development needs(16,17).

In poorly resourced health systems where people present late with disease, amputation rates and subsequent mortality is high. For example, a small study in Tanzania showed that 33% of people admitted to hospital with diabetic foot disease were managed by amputation and mortality was nearly 50%(18). By comparison, in the UK 17% of people presenting with disease underwent amputation within 1 year of presentation with no reported mortality (19). After limb-loss, poor post-operative care and the dearth of services for rehabilitation, prostheses, and mobility aids result in further mortality and morbidity (20). The devastating effects are tragic given there are simple and cheap strategies – including preventing and treating CVDRF and regular monitoring for signs of LED in high risk individuals with early referral to specialist services - to reduce deleterious outcomes.

Although this is one of the few studies to look at population prevalence of LED, (7) the findings of this study are in keeping with previously published work(8,21). For example, population prevalence of vascular disease has been reported at over 30% in those over 65 years (7).

An interesting ‘U ’shaped association between LED and wealth was observed, with the greatest prevalence of disease in the poorest and the wealthiest groups. The prevalence of hypertension and diabetes in these groups was also significantly higher at the extremes of wealth than in the middle 3 quintiles, which may go some way to explaining the observations. There may be other factors, beyond the scope of this study to characterise, such as lack of access to skilled healthcare services for optimum management of these comorbidities in the poorer communities (22), and potentially other social drivers towards worse ill-health such as sedentary lifestyle and westernised dietary habits amongst the wealthier group, which somewhat negate improved access to healthcare for those who are more wealthy (23–25). It will be important to fully understand these associations in future work in order to understand which interventions may be the most effective in preventing LED amongst different population groups.

Smoking status in this study was not shown to be a significant factor associated with LED, in contrast to a well-established body of evidence from high income countries(26). The reasons for this are not clear, however smoking is not as common in Malawi as in some high income countries and the cut off for being defined as a smoker in this study was more than 6 cigarettes per week, which is a relatively low level of consumption.

This was a large scale population based study, highlighting those at risk of limb loss amongst a group with known CVDRF. In focusing on population level data, the study reduces the potential for bias towards those who have already engaged with healthcare services. This

is an advantage over much of the published literature to date, and provides a greater understanding of the scale of LED across different population groups in Malawi. The use of multiple approaches to evaluate the presence of LED is also a strength of this study, recognising that no single assessment is either sensitive or specific in isolation(4).

There are potential limitations to the conclusions to be drawn from this study. The use of ABPI and the Edinburgh Claudication Questionnaire as surrogate markers for peripheral vascular disease, whilst both validated techniques, do have drawbacks. Claudication, in itself, may never progress to represent a limb threatening condition, however can be a good marker of more generalised cardiovascular ill-health and therefore highlight opportunities for optimisation of best medical therapy in order to reduce cardiovascular mortality(27). ABPI may be confounded by calcification in vessels, resulting in falsely elevated ratios; this is particularly seen amongst diabetic individuals. The inclusion of those with ABPI>1.2 into the group with ABPI 0.9-1.2 may have resulted in some false negative results, neglecting to report arterial disease which was in fact present. Whilst toe brachial pressure index (TBPI) may be a more reliable observation in this group(28), there is little in the published data on TBPI studies in SSA and therefore interpretation of the findings from this study in the context of previously published work would have been more limited had this technique been utilised. We also did not enquire about the aetiology of ulcers hence it is not possible to establish the aetiology of the ulcers. Finally, we failed to achieve the sample size for this study, however the proportion of participants affected by LED was far greater than we had estimated and would have required a lower number of participants to reliably detect. In a retrospective calculation, 460 participants would have been required to detect a prevalence of LED of 24% with the same width of confidence interval as in our initial power calculation.

In conclusion, this study demonstrated that 1 in 4 individuals with two or more cardiovascular risk factors have evidence of lower extremity disease and 2.5% already had active signs of limb threatening ulceration or previous amputation. An interesting U-shaped association with wealth was observed. Future work will need to explore the social determinants of health in relation to LED in order to guide health system interventions appropriately; the solutions may be complex and it is likely that there will not be a ‘one size fits all ’approach to preventing limb loss in this region. It is likely that both therapeutic and preventive services will have to be developed in parallel.

References

1. Pillay B, Ramdial P, Naidoo D. HIV-associated large-vessel vasculopathy: a review of the current and emerging clinicopathological spectrum in vascular surgical practice: review article. Cardiovasc J Afr. 2015 Apr 30;26(2):70–81.
2. Madsen UR, Baath C, Berthelsen CB, Hommel A. Age and health-related quality of life, general self-efficacy, and functional level 12 months following dysvascular major lower limb amputation: a prospective longitudinal study. Disabil Rehabil. 2019 Nov 20;41(24):2900–9.
3. Taghipour H, Moharamzad Y, Mafi AR, Amini A, Naghizadeh MM, Soroush MR, et al. Quality of Life Among Veterans With War-Related Unilateral Lower Extremity Amputation: A Long-Term Survey in a Prosthesis Center in Iran: J Orthop Trauma. 2009 Aug;23(7):525–30.
4. Johnston LE, Stewart BT, Yangni-Angate H, Veller M, Upchurch GR, Gyedu A, et al. Peripheral Arterial Disease in Sub-Saharan Africa: A Review. JAMA Surg. 2016 Jun 1;151(6):564.
5. Fowkes FGR, Rudan D, Rudan I, Aboyans V, Denenberg JO, McDermott MM, et al. Comparison of global estimates of prevalence and risk factors for peripheral artery disease in 2000 and 2010: a systematic review and analysis. The Lancet. 2013 Oct;382(9901):1329–40.
6. Joshi R, Jan S, Wu Y, MacMahon S. Global Inequalities in Access to Cardiovascular Health Care. J Am Coll Cardiol. 2008 Dec;52(23):1817–25.
7. Guerchet M, Aboyans V, Mbelesso P, Mouanga AM, Salazar J, Bandzouzi B, et al. Epidemiology of Peripheral Artery Disease in Elder General Population of Two Cities of Central Africa: Bangui and Brazzaville. Eur J Vasc Endovasc Surg. 2012 Aug;44(2):164–9.
8. Yeboah K, Puplampu P, Ainuson J, Akpalu J, Gyan B, Amoah AGB. Peripheral artery disease and exertional leg symptoms in diabetes patients in Ghana. BMC Cardiovasc Disord. 2016 Dec;16(1):1–9.
9. Chisala P, Sandford B. Peripheral arterial disease among high-risk patients at a tertiary hospital in Blantyre, Malawi: A 2-phase observational study. East Cent Afr J Surg. 2020;25(3).
10. Okello S, Millard A, Owori R, Asiimwe SB, Siedner MJ, Rwebembera J, et al. Prevalence of lower extremity Peripheral artery disease among adult diabetes patients in Southwestern Uganda. BMC Cardiovasc Disord. 2014 Dec;14(1):75.

11. Price AJ, Crampin AC, Amberbir A, Kayuni-Chihana N, Musicha C, Tafatatha T, et al. Prevalence of obesity, hypertension, and diabetes, and cascade of care in sub-Saharan Africa: a cross-sectional, population-based study in rural and urban Malawi. Lancet Diabetes Endocrinol. 2018 Mar;6(3):208–22.

12. Aboyans V. The intra- and interobserver variability of ankle–arm blood pressure index according to its mode of calculation. J Clin Epidemiol. 2003 Mar;56(3):215–20.

13. Aboyans V, Criqui MH, Abraham P, Allison MA, Creager MA, Diehm C, et al. Measurement and Interpretation of the Ankle-Brachial Index: A Scientific Statement From the American Heart Association. Circulation. 2012 Dec 11;126(24):2890–909.

14. Chikowe I, Mwapasa V, Kengne AP. Analysis of rural health centres preparedness for the management of diabetic patients in Malawi. BMC Res Notes. 2018 Dec;11(1):267.

15. Assayed AA, Muula AS, Nyirenda MJ. The quality of care of diabetic patients in rural Malawi: A case of Mangochi district. Malawi Med J. 2014;6.

16. Veller MG. Education in vascular surgery–critical issues: A Southern African perspective. J Vasc Surg. 2008 Dec 1;48(6):84S-86S.

17. Magnusson L, Ahlström G, Ramstrand N, Fransson EI. Malawian prosthetic and orthotic users 'mobility and satisfaction with their lower limb assistive device. J Rehabil Med. 2013 Apr;45(4):385–91.

18. Gulam-Abbas Z, Lutale JK, Morbach S, Archibald LK. Clinical outcome of diabetes patients hospitalized with foot ulcers, Dar es Salaam, Tanzania. Diabet Med. 2002 Jul;19(7):575–9.

19. Paisey RB, Abbott A, Levenson R, Harrington A, Browne D, Moore J, et al. Diabetes-related major lower limb amputation incidence is strongly related to diabetic foot service provision and improves with enhancement of services: peer review of the South-West of England. Diabet Med. 2018 Jan;35(1):53–62.

20. Guest JF, Fuller GW, Vowden P. Diabetic foot ulcer management in clinical practice in the UK: costs and outcomes. Int Wound J. 2018;15(1):43–52.

21. Zhang P, Lu J, Jing Y, Tang S, Zhu D, Bi Y. Global epidemiology of diabetic foot ulceration: a systematic review and meta-analysis. Ann Med. 2017 Feb 17;49(2):106–16.

22. Angwenyi V, Aantjes C, Kajumi M, De Man J, Criel B, Bunders-Aelen J. Patients experiences of self-management and strategies for dealing with chronic conditions in rural Malawi. Van Bogaert P, editor. PLOS ONE. 2018 Jul 2;13(7):e0199977.

23. Geldsetzer P, Manne-Goehler J, Marcus M-E, Ebert C, Zhumadilov Z, Wesseh CS, et al. The state of hypertension care in 44 low-income and middle-income countries: a cross-sectional study of nationally representative individual-level data from 1·1 million adults. The Lancet. 2019 Aug;394(10199):652–62.
24. Manne-Goehler J, Geldsetzer P, Agoudavi K, Andall-Brereton G, Aryal KK, Bicaba BW, et al. Health system performance for people with diabetes in 28 low- and middle-income countries: A cross-sectional study of nationally representative surveys. Wareham NJ, editor. PLOS Med. 2019 Mar 1;16(3):e1002751.
25. Kreatsoulas C, Frcpc SSA. The impact of social determinants on cardiovascular disease. 2010;26:6.
26. How Tobacco Smoke Causes Disease: The Biology and Behavioral Basis for Smoking-Attributable Disease. U.S. Department of Health and Human Services, Centers for Disease Control and Prevention, National Center for Chronic Disease Prevention and Health Promotion, Office on Smoking and Health; 2010.
27. Bevan GH, White Solaru KT. Evidence-Based Medical Management of Peripheral Artery Disease. Arterioscler Thromb Vasc Biol. 2020 Mar;40(3):541–53.
28. Williams DT, Harding KG, Price P. An Evaluation of the Efficacy of Methods Used in Screening for Lower-Limb Arterial Disease in Diabetes. Diabetes Care. 2005 Sep 1;28(9):2206–10.

Table 1: Summary of participant characteristics

Variables	N (%)	Both sites		Karonga (rural)		Lilongwe (urban)	
		men	women	men	women	men	women
**TOTAL**		**431**	**375**	**303**	**154**	**128**	**221**
**Age**							
Under 50	346(42.9)	199(46.2)	147 (39.2)	138(45.5)	51 (33.1)	61 (47.7)	96 (43.4)
50—60	239(29.7)	113 (26.2)	126 (33.6)	80 (26.4)	49 (31.8)	33 (25.8)	77 (34.8)
Over 60	221(27.4)	119 (27.6)	102 (27.2)	85 (28.1)	54 (35.1)	34 (26.6)	48 (21.7)
**Wealth quintiles**							
poorest	108 (13.4)	41 (9.5)	67 (17.9)	34 (11.2)	51 (33.1)	7 (5.5)	16 (7.2)
second	150 (18.6)	83 (19.3)	67 (17.9)	80 (26.4)	46 (29.9)	3 (2.3)	21 (9.5)
third	139 (17.3)	85 (19.7)	54 (14.4)	77 (25.4)	29 (18.8)	8 (6.3)	25 (11.3)
fourth	191 (23.7)	103 (23.9)	88 (23.5)	66 (21.8)	18 (11.7)	37 (28.9)	70 (31.7)
wealthiest	218 (27.1)	119 (27.6)	99 (26.4)	46 (15.2)	10 (6.5)	73 (57)	89 (40.3)
**Marital status**							
single	209 (25.9)	54 (12.5)	155 (41.3)	36 (11.9)	82 (53.3)	18 (14.1)	73 (33.0)
married	597 (74.1)	377 (87.5)	220 (58.7)	267 (88.1)	72 (46.8)	110 (85.9)	148 (67.0)
**highest attained education**							

0—5yrs primary education	164 (20.4)	59 (13.7)	105 (28)	48 (15.8)	66 (42.9)	11 (8.6)	39 (17.7)
Standard 6-8	329 (40.8)	184 (42.7)	145 (38.7)	156 (51.5)	65 (42.2)	28 (21.9)	80 (36.2)
secondary	230 (28.5)	141 (32.7)	89 (23.7)	94 (31.0)	21 (13.6)	47 (36.7)	68 (30.8)
Post-secondary	83 (10.3)	47 (10.9)	36 (9.6)	5 (1.7)	2 (1.3)	42 (32.8)	34 (15.4)
**Occupation**							
homemaker	237 (29.4)	74 (17.2)	163 (43.5)	32 (10.6)	34 (22.1)	42 (32.8)	129 (58.4)
Farming/fishing	296 (36.7)	209 (48.5)	87 (23.2)	208 (68.7)	84 (54.6)	1 (0.8)	3 (1.36)
employed	273 (33.9)	148 (34.3)	125 (33.3)	63 (20.8)	36 (23.4)	85 (66.4)	89 (40.3)
**Indoor fire**							
no	42 (5.2)	26 (6.0)	16 (4.3)	17 (5.6)	6 (3.9)	9 (7.0)	10 (4.5)
Yes, inside the house	39 (4.8)	19 (4.4)	20 (5.3)	5 (1.7)	3 (2.0)	14 (10.9)	17 (7.7)
Yes, but in a separate kitchen	725 (90.0)	386 (89.6)	339 (90.4)	281 (92.7)	145 (94.2)	105 (82)	194 (87.8)
**Smoking history**							
Non- smokers	487 (60.4)	130 (30.2)	357 (95.2)	72 (23.8)	142 (92.2)	58 (45.3)	215 (97.3)
Smokers	319 (39.6)	301 (69.8)	18 (4.8)	231 (76.2)	12 (7.8)	70 (54.7)	6 (2.7)
**Diabetes**							

Non-diabetic	502 (62.3)	324 (75.2)	324 (75.2)	251 (82.8)	88 (57.1)	73 (57)	90 (40.7)
Diabetic	273 (33.9)	87 (20.2)	87 (20.2)	42 (13.9)	62 (40.3)	45 (35.2)	124 (56.1)
Missing data	31 (3.9)	20 (4.6)	20 (4.6)	10 (3.3)	4 (2.6)	10 (7.8)	7 (3.2)
**Dyslipidaemia**							
yes	344 (42.7)	238 (55.2)	175 (46.7)	177 (58.4)	73 (47.4)	61 (47.7)	102 (46.2)
no	413 (51.2)	165 (38.3)	179 (47.7)	114 (37.6)	74 (48.1)	51 (39.8)	105 (47.5)
Missing data	49 (6.1)	28 (6.5)	21 (5.6)	12 (4.0)	7 (4.6)	16 (12.5)	14 (6.3)
**Hypertension**							
hypertensive	482 (59.8)	220 (51.0)	104 (27.7)	171 (56.4)	40 (26.0)	49 (38.3)	64 (29.0)
Not hypertensive	324 (40.2)	211 (49.0)	271 (72.3)	132 (43.6)	114 (74.0)	79 (61.7)	157 (71.0)
**Mean blood pressure (SD)**							
	139.1 (38.2)	136 (41.6)	142.6 (33.7)	137 (47)	149.1 (43.6)	133.7 (24.1)	138.1 (23.6)
**HIV**							
negative	413 (51.2)	237 (55.0)	176 (46.9)	170 (56.1)	80 (52.0)	67 (52.3)	96 (43.4)
positive	247 (30.7)	107 (24.8)	140 (37.3)	78 (25.7)	57 (37.0)	29 (22.7)	83 (37.6)
unknown	146 (18.1)	87 (20.2)	59 (15.7)	55 (18.2)	17 (11.0)	32 (25)	42 (19)
**Obesity**							
No	634 (78.7)	402 (93.3)	232 (61.9)	292 (96.4)	113 (73.4)	110 (85.9)	119 (53.9)

Yes	172 (21.3)	29 (6.7)	143 (38.1)	11 (3.6)	41 (26.6)	18 (14.1)	102 (46.2)

1

Table 2: prevalence of LED in total and by participant characteristics

Variables	N	LED prevalence (95% CI) n=194
**Total**	806	24.1 (21.2—27.2)
**Age**		
<50	346	15.9 (12.2—20.2)
50-60	239	27.6 (22.0—33.7)
>60	221	33.0 (26.9—39.7)
**Sex**		
Male	431	15.8 (12.5—19.6)
Female	375	33.6 (28.8— 38.6)
**Residence**		
Rural	457	17.5 (14.1—21.3)
Urban	349	32.7 (27.8—37.9)
**Wealth quintiles**		
poorest	108	32.4 (16.2—30.2)
second	150	22.7 (16.2—30.2)
third	139	15.8 (10.2—23.0)
fourth	191	24.1 (18.2—30.8)
wealthiest	218	26.1 (20.4—32.5)
**Marital status**		
Single	209	34.0 (27.6—40.8)
married	587	20.6 (17.4—24.1)

For peer review only - http://bmjopen.bmj.com/site/about/guidelines.xhtml

**Educational achievement**		
0-5 years primary education	164	25 (18.6—32.3)
Standard 6-8	329	25.5 (20.9—30.6)
Secondary	230	21.7 (16.6—27.6)
Post-secondary	83	22.9 (14.4—33.4)
**occupation**		
homemaker	237	37.1 (31.0—43.6)
Farming/ fishing	296	15.5 (11.6—20.2)
employed	273	22.0 (17.2—27.4)
**Indoor fire**		
none	42	7.1 (1.5—19.5)
Fire usually lit Inside the house	39	30.8 (17.0—47.6)
Fire usually lit in a separate kitchen area	725	24.7 (21.6—28.0)
**Any Smoking history**		
no	487	29.4 (25.4—33.6)
yes	319	16.0 (12.1—20.5)
**Diabetes**		
Non-diabetic	502	19.7 (16.3—23.5)
diabetic	273	33.7 (28.1—39.6)
Missing data	31	9.7 (2.0—25.8)
**Dyslipidaemia**		
no	413	22.8 (18.8—27.1)
yes	344	26.5 (21.9—31.5)
Missing data	49	18.4 (8.8—32.0)
**HIV**		
negative	413	23.0 (19.0—27.4)
positive	247	23.9 (18.7—29.7)
unknown	146	27.4 (20.3—35.4)
**hypertension**		
Not-hypertensive	324	21.9 (17.6—26.8)
hypertensive	482	25.5 (21.7—29.7)
**obesity**		

no	634	20.8 (17.7—24.2)
yes	172	36.0 (28.9—43.7)
**Number of risk factors**		
2	201	14.9 (10.3—20.6)
3	342	23.1 (18.7—27.9)
4	204	32.4 (26.0 —39.2)
≥5	59	32.2 (20.6—45.6)
**Prevalence of outcomes by risk factors**		
No diabetes and no hypertension	193	14.0 (25.6—42.4)
Diabetes but no hypertension	131	33.6 (25.6—42.4)
Hypertension but no diabetes	340	22.1 (17.8—26.8)
Hypertension and diabetes	142	33.8 (26.1—42.2)

Figure Legends

Figure 1: Flowcharts of study participation by site

Figure 2: three major risk factors and multi-morbidity across the study population

Figure 3: Coefficient plot of factors associated with lower extremity disease

For peer review only

FIGURE 1: Flowcharts of study participation by site

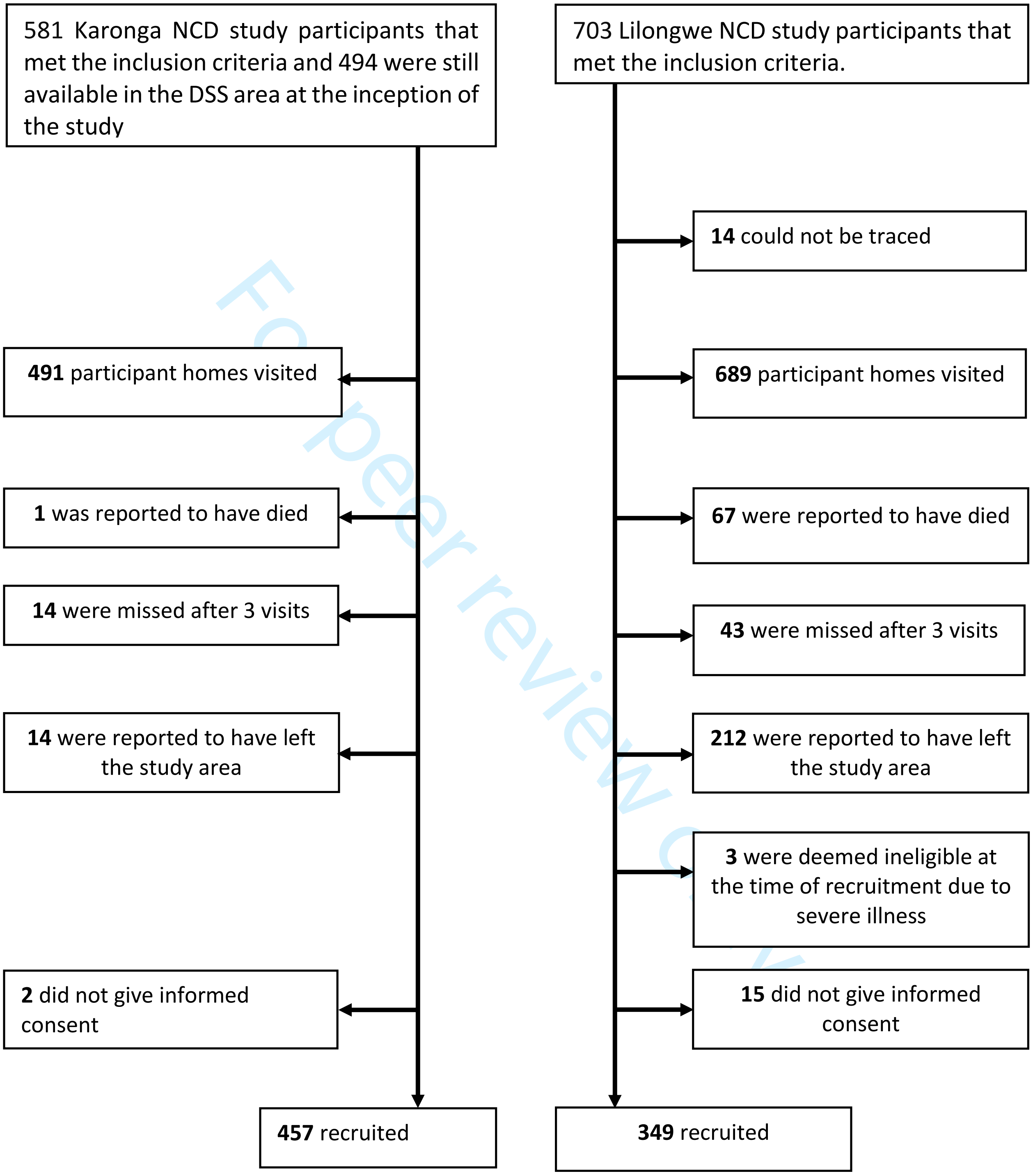

For peer review only

FIGURE 2: three major risk factors and multi-morbidity across the study population

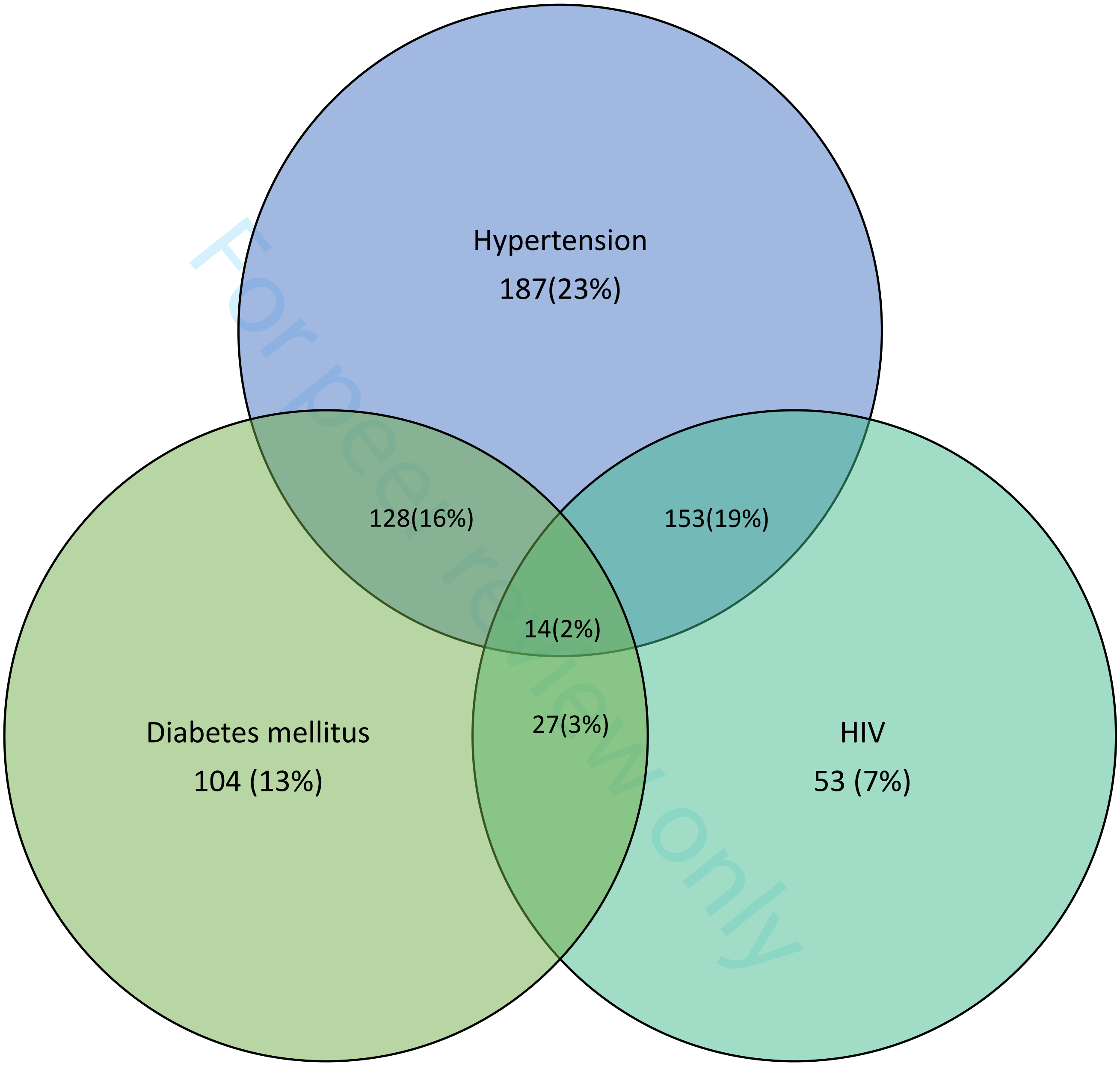

1

For peer review only

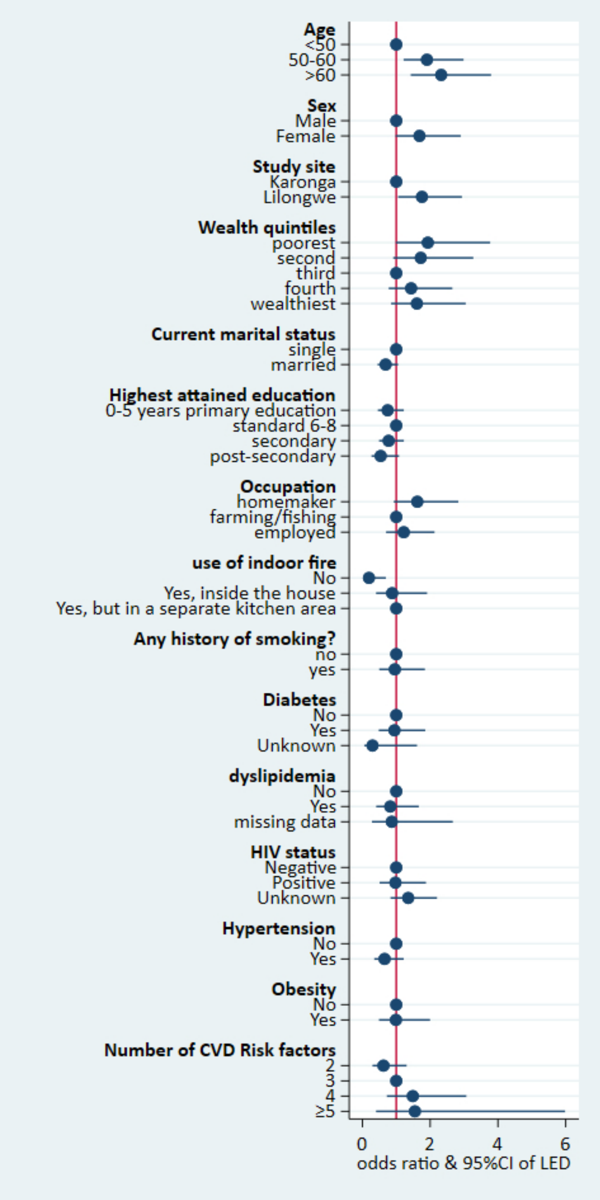

Coefficient plot of factors associated with lower extremity disease

125x250mm (96 x 96 DPI)

Appendices

Table 1: Crude prevalence of factors associated with LED

		OUTCOME						
	N	LED prevalence (95% CI) n=194	Absent pulse prevalence (95% CI) n=54	Amputation Prevalence (95% CI) n=7	Ulcer Prevalence (95% CI) n=13	Claudication Prevalence (95% CI) n=81	Neuropathy Prevalence (95% CI) n=103	ABPI<0.9 Prevalence (95% CI) n=18
**Total**								
	806	24.1 (21.2—27.2)	6.7 (5.1—8.7)	0.9 (0.3—1.8)	1.1 (0.4— 1.8)	10 (8.1—12.3)	12.8 (10.6—15.3)	1.9 (1.0—3.1)
**Age**								
<50	346	15.9 (12.2—20.2)	4.9 (2.9—7.8)	0.3 (0.0—1.6)	2.3 (1.0—4.5)	9.0 (6.2—12.5)	6.1 (3.8—9.1)	0.3 (0.0—1.6)
50-60	239	27.6 (22.0—33.7)	6.7 (3.9—10.6)	2.1 (0.7—4.8)	0.8 (0.1—3.0)	12.6 (8.6—17.4)	15.1 (10.8—20.2)	0.8 (0.1—3.0)
>60	221	33.0 (26.9—39.7)	9.5 (6.0—14.2)	0.5 (0.0—2.5)	1.4 (0.3—3.9)	9.0 (5.6—13.6)	20.8 (15.7—26.8)	5.4 (2.8—9.3)
**Sex**								
Male	431	15.8 (12.5—19.6)	5.8 (3.8—8.4)	0.5 (0.1—1.7)	1.4 (0.5—3.0)	4.2 (2.5—6.5)	10.2 (7.5—13.5)	0.9 (0.3—2.4)
Female	375	33.6 (28.8— 38.6)	7.7 (5.2—10.9)	1.3 (0.4—3.1)	1.9 (0.8— 3.8)	16.8 (13.2—21.0)	15.7 (12.2—19.8)	2.9 (1.5—5.2)
**Residence**								
Rural	457	17.5 (14.1—21.3)	9.0 (6.5—12.0)	1.1 (0.4—2.5)	1.8 (0.8—3.4)	2.2 (1.1— 4.0)	13.8 (10.8—17.3)	1.8 (0.8—3.4)
Urban	349	32.7 (27.8—37.9)	3.7 (2.0—6.3)	0.6 (0.1— 2.1)	1.4 (0.5—3.3)	20.3 (16.2—25.0)	11.5 (8.3—15.3)	2.0 (0.8—4.1)
**Wealth quintiles**								
poorest	108	32.4 (16.2—30.2)	13.0 (7.3—20.8)	0.9 (0.02—5.1)	0.9 (0.02—5.1)	7.5 (2.6—12.9)	25 (17.2—34.3)	5.6 (2.1—11.7)

For peer review only

For peer review only - http://bmjopen.bmj.com/site/about/guidelines.xhtml

second	150	22.7 (16.2—30.2)	5.3 (2.3—10.2)	0.0 (0—2.4)	0.6 (0.02—3.7)	7.3 (3.7—12.7)	14.0 (8.9—20.6)	2.0 (0.4—5.7)
third	139	15.8 (10.2—23.0)	6.5 (3.0—11.9)	0.0 (0—2.6)	1.4 (0.2—5.1)	6.5 (3.0—11.9)	8.6 (4.5—14.6)	0.7 (0.0—3.9)
fourth	191	24.1 (18.2—30.8)	4.7 (2.2—8.8)	2.1 (0.6—5.3)	0.5 (0.0—2.9)	13.1 (8.7—18.7)	11.0 (6.9—16.3)	1.6 (0.3—4.5)
wealthiest	218	26.1 (20.4—32.5)	6.4 (3.6—10.5)	0.9 (0.1—3.3)	3.7 (1.6—7.1)	13.3 (9.1—18.5)	10.1 (6.4—15.9)	0.9 (0.1—3.3)
Marital status								
Single	209	34.0 (27.6—40.8)	10.0 (6.3—15.0)	1.0 (0.1—3.4)	1.9 (0.5—4.8)	12.4 (8.2—17.7)	18.7 (13.6—24.6)	3.8 (1.7—7.4)
married	587	20.6 (17.4—24.1)	5.5 (3.8—7.7)	0.8 (0.3—1.9)	1.5 (0.7—2.8)	9.2 (7.0—11.8)	10.7 (8.4—13.5)	1.2 (0.5—2.4)
Educational achievement								
0-5 years primary education	164	25 (18.6—32.3)	7.9 (4.3—13.2)	0 (0.0—2.2)	1.2 (0.1—4.3)	6.7 (3.4—11.7)	16.5 (11.1—23.0)	4.3 (1.7—8.6)
Standard 6-8	329	25.5 (20.9—30.6)	6.7 (4.2—9.9)	0.6 (0.1—2.2)	1.5 (0.5—3.5)	11.9 (8.6—15.8)	12.5 (9.1—16.5)	2.1 (0.9—4.3)
Secondary	230	21.7 (16.6—27.6)	6.1 (3.4—10.0)	1.7 (0.5—4.4)	2.6 (1.0—5.6)	8.2 (5.0—12.6)	12.2 (8.2—17.1)	0.4 (0.0—2.4)
Post-secondary	83	22.9 (14.4—33.4)	6.0 (2.0—13.5)	1.2 (0.0—6.5)	0.0 (0—4.3)	14.5 (7.7—23.9)	8.4 (3.5—16.6)	0.0 (0.0—4.3)
occupation								
homemaker	237	37.1 (31.0—43.6)	7.2 (4.2—11.2)	1.7 (0.5—4.3)	2.5 (0.9—5.4)	18.6 (13.8—24.1)	15.6 (11.2—20.9)	4.2 (2.0—7.6)
Farming/ fishing	296	15.5 (11.6—20.2)	9.1 (6.1—13.0)	0.3 (0.0—1.9)	1.0 (0.2—2.9)	1.7 (0.6—3.9)	12.8 (9.2—17.9)	1.7 (0.6—3.9)
employed	273	22.0 (17.2—27.4)	3.7 (1..8—6.6)	0.7 (0.0—2.6)	1.5 (0.4—3.7)	11.7 (8.2—16.1)	10.3 (6.9—14.5)	0.0 (0—1.3)
Indoor fire								
none	42	7.1 (1.5—19.5)	4.8 (0.6—16.2)	0.0 (0—8.4)	2.4 (0.0—12.6)	4.8 (0.6—16.2)	0.0 (0—8.4)	0.0 (0—8.4)
Fire usually lit Inside the house	39	30.8 (17.0—47.6)	7.7 (1.6—20.9)	0.0 (0—9.0)	2.6 (0.0—13.5)	17.9 (7.5—33.5)	12.8 (4.3—27.4)	0.0 (0—9.0)
Fire usually lit in a separate	725	24.7 (21.6—28.0)	6.8 (5.0—8.8)	1.0 (0.4—2.0)	1.5 (0.8—2.7)	9.9 (7.9—12.3)	13.5 (11.1—16.2)	2.1 (1.2—3.4)

kitchen area								
**Any Smoking history**								
no	487	29.4 (25.4—33.6)	7.6 (5.4—10.3)	1.2 (0.5—2.7)	1.8 (0.8—3.5)	14.0 (11.0—17.4)	14.4 (11.4—17.8)	2.1 (1.0—3.7)
yes	319	16.0 (12.1—20.5)	5.3 (3.1—8.4)	0.3 (0.0—1.7)	1.3 (0.3—3.2)	4.1 (2.2—6.9)	10.3 (7.2—14.2)	1.6 (0.5—3.6)
**Diabetes**								
Non-diabetic	502	19.7 (16.3—23.5)	6.2 (4.2—8.7)	0 (0—0.7)	1.2 (0.4—2.6)	8.2 (5.9—10.9)	10.2 (7.7—13.1)	1.4 (0.6—2.9)
diabetic	273	33.7 (28.1—39.6)	7.7 (4.8—11.5)	2.6 (1.0—5.2)	2.2 (0.8—4.7)	13.9 (10.0—18.6)	18.7 (14.2—23.8)	2.9 (1.3—5.7)
Missing data	31	9.7 (2.0—25.8)	6.5 (0.8—21.4)	0.0 (0—11.2)	3.2 (0.0–16.7)	6.5 (0.8—21.4)	3.2 (0.1—16.7)	0.0 (0.0—11.2)
**Dyslipidaemia**								
no	413	22.8 (18.8—27.1)	6.5 (4.4—9.4)	0.7 (0.2—2.1)	1.2 (0.4—2.8)	10.4 (7.6—13.8)	10.7 (7.8—14.0)	2.4 (1.2—4.4)
yes	344	26.5 (21.9—31.5)	7.0 (4.5—10.2)	1.2 (0.3—3.0)	1.7 (0.6—3.8)	10.2 (7.2—13.9)	15.7 (12.0—20.0)	1.5 (0.5—3.4)
Missing data	49	18.4 (8.8—32.0)	6.1 (1.3—16.9)	0.0 (0.0—7.3)	4.1 (0.5—14.0)	6.1 (1.3—16.9)	10.2 (3.4—22.2)	0.0 (0.0—7.3)
**HIV**								
negative	413	23.0 (19.0—27.4)	7.5 (5.2—10.5)	0.7 (0.2—2.1)	1.2 (0.4—2.8)	10.0 (7.2—13.2)	12.1 (9.1—15.6)	2.4 (1.2—4.4)
positive	247	23.9 (18.7—29.7)	6.1 (3.4—9.8)	0.4 (0.0—2.2)	2.0 (0.7—4.7)	10.1 (6.7—14.6)	12.6 (8.7—`17.3)	0.8 (0.1—2.9)
unknown	146	27.4 (20.3—35.4)	5.5 (2.4—10.5)	0.0 (0—2.5)	0.7 (0.0—3.8)	10.3 (5.9—16.4)	15.1 (9.7—21.9)	2.7 (0.8—6.9)
**hypertension**								
Not-hypertensive	324	21.9 (17.6—26.8)	4.6 (2.6—7.5)	0.9 (0.2—2.7)	2.2 (0.9—4.4)	9.3 (6.3—13.0)	12.7 (9.2—16.8)	0.6 (0.1—2.2)
hypertensive	482	25.5 (21.7—29.7)	8.1 (5.8—10.9)	0.8 (0.2—2.1)	1.2 (0.5—2.7)	10.6 (8.0—13.7)	12.9 (10.0—16.2)	2.7 (1.4—4.6)
**obesity**								

For peer review only

no	634	20.8 (17.7—24.2)	5.8 (4.1—8.0)	0.6 (0.2—1.6)	1.6 (0.8—2.9)	7.3 (5.4—9.6)	11.8 (9.4—14.6)	1.9 (1.0—3.3)
yes	172	36.0 (28.9—43.7)	9.9 (5.9—15.4)	1.7 (0.4—5.0)	1.7 (0.4—5.0)	20.3 (14.6—27.1)	16.3 (11.1—22.7)	1.7 (0.4—5.0)
Number of risk factors								
2	201	14.9 (10.3—20.6)	3.0 (1.1—6.4)	1.0 (0.1—3.5)	1.1 (0.1–3.5)	7.5 (4.2—12.0)	6.5 (3.4—10.8)	0.5 (0.0—2.7)
3	342	23.1 (18.7—27.9)	6.7 (4.3—9.9)	0.3 (0.0—1.6)	2.0 (0.8—4.2)	8.8 (6.0—12.3)	12.0 (8.7—15.9)	2.6 (1.2—4.9)
4	204	32.4 (26.0 —39.2)	9.3 (5.7—14.2)	0.5 (0.0—2.7)	1.0 (0.5—4.9)	14.7 (10.1—20.3)	18.1 (13.1—24.1)	2.0 (0.5—4.9)
≥5	59	32.2 (20.6—45.6)	10.2 (3.8—20.8)	5.1 (1.1—14.1)	0.0 (0.0—6.1)	10.2 (3.8—20.8)	20.3 (11.0—32.8)	1.7 (0.0—9.1)
Prevalence of outcomes by risk factors								
No diabetes and no hypertension	193	14.0 (25.6—42.4)	4.1 (1.8—8.0)	0.0 (0.0—1.9)	1.6 (0.3—4.4)	4.7 (2.2—8.7)	9.3 (5.6—14.3)	0.0 (0.0—1.9)
Diabetes but no hypertension	131	33.6 (25.6—42.4)	5.3 (2.2—10.7)	2.3 (0.5 –6.5)	3.1 (0.8—7.6)	16.0 (10.2—23.5)	17.6 (11.5—25.2)	1.5 (0.2—5.4)
Hypertension but no diabetes	340	22.1 (17.8—26.8)	7.4 (4.8—10.7)	0.0 (0.0 —1.1)	1.2 (0.3—3.0)	10 (7.0—13.7)	10.0 (7.0—13.7)	2.1 (0.8—4.2)
Hypertension and diabetes	142	33.8 (26.1—42.2)	9.9 (5.5—16.0)	2.8 (0.8 —7.1)	1.4 (0.2 —5.0)	12.0 (7.1—18.5)	19.7 (13.5—27.2)	4.2 (1.6—9.0)

For peer review only

Table 2: full regression model of factors associated with Lower extremity disease

	Odds Ratio	Std. Err.	z	P>z	95% confidence interval	Lr test p value
Age						
<50	1	ref.				0.0012
50-60	1.91	0.4	2.8	0.0	1.22—2.99	
>60	2.33	0.6	3.4	0.0	1.43—3.8	
sex						
Male	1.0	ref.				0.0574
Female	1.69	0.5	1.9	0.1	0.98—2.9	
site						
Karonga	1.0	ref.				0.0296
Lilongwe	1.76	0.5	2.2	0.0	1.05—2.94	
Wealth quintiles						
poorest	1.93	0.7	1.9	0.1	0.99—3.77	0.3322
second	1.72	0.6	1.7	0.1	0.91—3.27	
third	1.0	ref.				
fourth	1.43	0.4	1.2	0.2	0.78—2.66	
wealthiest	1.61	0.5	1.5	0.1	0.85—3.05	
Marital status						
single	1.0	ref.				0.0862
married	0.69	0.1	-1.7	0.1	0.45—1.05	
Highest attained education						
years primary education	0.75	0.2	-1.2	0.2	0.46—1.22	0.2630
standard 6-8	1.0	ref.				
secondary	0.78	0.2	-1.1	0.3	0.49—1.22	
post-secondary	0.54	0.2	-1.7	0.1	0.27—1.08	
Occupation						
homemaker	1.62	0.5	1.7	0.1	0.93—2.83	0.2026
farming/fishing	1.0	ref.				
employed	1.22	0.3	0.7	0.5	0.7—2.13	
Indoor smoke						
No	0.19	0.1	-2.5	0.0	0.05—0.69	0.0105
Yes, inside the house	0.88	0.3	-0.3	0.7	0.4—1.91	
Yes, in a separate kitchen area	1.0	ref.				
any history of smoking						
no	1.0	ref.				0.8995
yes	0.96	0.3	-0.1	0.9	0.5—1.84	
Diabetes						
No	1.0	ref.				0.3532
Yes	0.95	0.3	-0.2	0.9	0.48—1.86	

For peer review only

Unknown	0.3	0.3	-1.4	0.2	0.06—1.61	
**Dyslipidaemia**						
No	1.0	ref.				0.8425
Yes	0.82	0.3	-0.5	0.6	0.41—1.66	
missing data	0.87	0.5	-0.3	0.8	0.28—2.67	
**HIV status**						
Negative	1.0	ref.				0.4490
Positive	0.98	0.3	-0.1	0.9	0.51—1.88	
Unknown	1.35	0.3	1.2	0.2	0.83—2.2	
**hypertension**						
No	1.0	ref.				0.1768
Yes	0.65	0.2	-1.3	0.2	0.35—1.22	
**obesity**						
No	1.0	ref.				0.9754
Yes	0.99	0.4	0.0	1.0	0.49—2.0	
**CVDRF**						
2	0.62	0.2	-1.3	0.2	0.3—1.3	0.4812
3	1.0	ref.				
4	1.49	0.5	1.1	0.3	0.72—3.07	
≥5	1.54	1.1	0.6	0.5	0.4—5.98	

For peer review only

Stratified analysis for lower extremity disease

Table 3: stratification by sex

	Male	Female
**Age**		
<50	(ref)	(ref)
50-60	1.96 (0.91—4.22)	1.97 (1.08—3.6)
>60	2.41 (1.08—5.37)	2 (1.0*—4.01)
**site**		
Karonga	(ref)	(ref)
Lilongwe	1.84 (0.79—4.31)	1.65 (0.82—3.34)
**Wealth quintiles**		
poorest	1.92 (0.6—6.19)	2.04 (0.86—4.88)
second	2.32 (0.87—6.18)	1.4 (0.58—3.39)
third	(ref)	(ref)
fourth	1.74 (0.65—4.64)	1.51 (0.66—3.46)
wealthiest	1.23 (0.42—3.6)	2.03 (0.87—4.72)
**Marital status**		
single	(ref)	(ref)
married	0.39 (0.18—0.86)	0.81 (0.47—1.39)
**Highest attained education**		
0-5 years prima..	1.01 (0.41—2.48)	0.69 (0.36—1.3)
standard 6-8	(ref)	(ref)
secondary	0.86 (0.42—1.73)	0.68 (0.36—1.28)
post-secondary	0.62 (0.19—2.06)	0.43 (0.17—1.09)
**Occupation**		
homemaker	1.09 (0.44—2.72)	2.19 (0.98—4.89)
farming/fishing	(ref)	(ref)
employed	0.91 (0.4—2.06)	1.87 (0.8—4.38)
**Indoor fire**		
No	(empty)	0.28 (0.07—1.17)
Yes, inside the house	0.66 (0.17—2.59)	1.06 (0.39—2.92)
Yes, but in a separate kitchen area.	(ref)	(ref)
**History of smoking**		
no	(ref)	(ref)
yes	0.79 (0.34—1.8)	1.83 (0.52—6.47)
**Diabetes**		
No	(ref)	(ref)
Yes	1.89 (0.71—5.01)	0.38 (0.13—1.18)
Unknown	0.48 (0.05—5.04)	0.18 (0.01—2.69)
**Dyslipidaemia**		

* Rounded up from .9989416

For peer review only

No	(ref)	(ref)
Yes	0.91 (0.32—2.59)	0.48 (0.16—1.49)
missing data	1.05 (0.18—6.21)	0.6 (0.12—2.97)
HIV status		
Negative	(ref)	(ref)
Positive	1.81 (0.74—4.41)	0.38 (0.13—1.13)
Unknown	1.69 (0.8—3.58)	0.93 (0.46—1.85)
hypertension		
No	(ref)	(ref)
Yes	0.52 (0.22—1.24)	0.39 (0.13—1.19)
obesity		
No	(ref)	(ref)
Yes	2.13 (0.64—7.07)	0.5 (0.17—1.48)
CVDRF		
2	0.68 (0.23—1.97)	0.45 (0.14—1.47)
3	(ref)	(ref)
4	1.37 (0.48—3.94)	2.59 (0.82—8.2)
≥5	0.76 (0.09—6.61)	6.48 (0.73—57.42)

For peer review only

Table 4: Stratification by Sex and history of smoking

Variables and variable categories	Male non-smoker	Male smoker	Female non-smoker	Female current smokers[†]
**Age**				
<50	(ref)	(ref)	(ref)	
50-60	2.74 (0.5—14.91)	1.43 (0.53—3.85)	2.1 (1.15—3.85)	
>60	4.53 (0.62—32.88)	1.86 (0.68—5.06)	1.95 (0.96—3.94)	
**Site**				
Karonga	(ref)	(ref)	(ref)	
Lilongwe	0.63 (0.12—3.41)	3.38 (1.07—10.62)	1.82 (0.88—3.78)	
**Wealth Quintile**				
poorest	6.26 (0.26—150.93)	1.58 (0.35—7.09)	1.71 (0.7—4.18)	
second	4.03 (0.18—89)	2.85 (0.9—9.05)	1.38 (0.56—3.43)	
third	(ref)	(ref)	(ref)	
fourth	4.67 (0.28—76.66)	1.27 (0.41—3.99)	1.24 (0.54—2.87)	
wealthiest	14.33 (0.91—225.09)	0.72 (0.16—3.21)	1.62 (0.69—3.81)	
**Marital status**				
single	(ref)	(ref)	(ref)	
married	0.04 (0.0—0.56)	0.58 (0.21—1.58)	0.91 (0.52—1.58)	
**Education**				
years primary education	4.93 (0.17—139.91)	0.88 (0.33—2.35)	0.64 (0.33—1.24)	
standard 6-8	(ref)	(ref)	(ref)	
secondary	3.02 (0.57—15.97)	0.62 (0.24—1.57)	0.78 (0.41—1.49)	
post-secondary	1.35 (0.19—9.71)	0.75 (0.07—8.19)	0.47 (0.19—1.18)	
**Occupation**				
homemaker	1.82 (0.29—11.26)	0.72 (0.2—2.6)	2.09 (0.9—4.88)	
farming/fishing	(ref)	(ref)	(ref)	
employed	0.43 (0.07—2.8)	1.1 (0.38—3.23)	1.68 (0.69—4.08)	
**Indoor smoke**				
No	(empty)	(empty)	(empty)	
Yes, inside the house	(empty)	0.82 (0.17—3.98)	1.02 (0.37—2.8)	
a separate kitchen area	(ref)	(ref)	(ref)	
**diabetes**				
No	(ref)	(ref)	(ref)	
Yes	1.57 (0.11—22.08)	3.79 (0.94—15.32)	0.35 (0.1—1.22)	
Unknown	(empty)	0.58 (0.02—16.66)	0.24 (0.02—3.85)	
**Dyslipidaemia**				
No	(ref)	(ref)	(ref)	
Yes	0.17 (0.01—3.9)	0.79 (0.2—3.14)	0.45 (0.13—1.53)	
missing data	0.28 (0.01—5.91)	0.7 (0.03—14.21)	0.57 (0.1—3.22)	
**HIV status**				
Negative	(ref)	(ref)	(ref)	

[†] No observations

Positive	1.82 (0.12—28.15)	2 (0.65—6.15)	0.41 (0.13—1.33)	
Unknown	2.01 (0.41—9.92)	1.74 (0.65—4.71)	0.99 (0.48—2.02)	
**hypertension**				
No	(ref)	(ref)	(ref)	
Yes	0.65 (0.06—6.93)	0.35 (0.1—1.15)	0.37 (0.11—1.25)	
**obesity**				
No	(ref)	(ref)	(ref)	
Yes	0.59 (0.04—9.2)	6.62 (1.06—41.46)	0.52 (0.16—1.72)	
**CVDRF**				
2	0.66 (0.04—11.7)	0.46 (0.11—1.85)	0.41 (0.11—1.49)	
3	(ref)	(ref)	(ref)	
4	20.29 (0.76—539.61)	0.51 (0.11—2.41)	2.67 (0.76—9.37)	
5	62.07 (0.17—22604.06)	0.26 (0.01—7.99)	6.71 (0.61—73.19)	

For peer review only

Table 5: Stratification by obesity and wealth quintiles

Variables and variable categories	non obese— Wealth quintile 1*	Non obese—wealth quintile 2	Non obese—wealth quintile 3	Non obese—wealth quintile 4	Non obese wealth quintile 5	Obese WQ1*‡	Obese WQ2*§	Obese—wealth quintile 3	Obese—wealth quintile 4	Obese—wealth quintile 5
**Age**										
<50	(ref)	(ref)	(ref)	(ref)	(ref)			1.45E-31 (.—.)	(ref)	(ref)
50-60	43.49 (1.51—1248.98)	4.57 (1.07—19.58)	0.8 (0.11—6.03)	2.63 (0.77—8.93)	0.85 (0.23—3.06)			5.54E-47 (.—.)	1.44 (0.07—30.58)	3.48 (0.5—24.14)
>60	12.11 (0.89—164.53)	4.43 (1.03—19.09)	0.42 (0.04—4.69)	3.82 (1.03—14.23)	2.18 (0.49—9.74)				1.91 (0.06—57.17)	2.65 (0.33—21.42)
**Sex**										
Male	(ref)	(ref)	(ref)	(ref)	(ref)			(ref)	(empty)	(ref)
Female	3.29 (0.44—24.85)	4.32 (0.64—28.94)	0.62 (0.03—13.88)	1.26 (0.32—4.93)	1.67 (0.45—6.18)			8.6E+107 (.—.)	(omitted)	1.9 (0.19—18.76)
**Site**										
Karonga	(ref)	(ref)	(ref)	(ref)	(ref)			(ref)	(empty)	(ref)
Lilongwe	12.72 (0.89—182.11)	3.95 (0.65—24.04)	2.64 (0.35—19.71)	2.49 (0.73—8.5)	0.53 (0.11—2.43)			2.6E+15 (.—.)	(omitted)	5.05 (0.13—202.03)
**Marital status**										
Single	(ref)	(ref)	(ref)	(ref)	(ref)			(ref)	(ref)	(ref)
married	1.39 (0.29—6.64)	0.95 (0.28—3.28)	0.29 (0.04—2.15)	0.5 (0.14—1.7)	0.64 (0.14—2.9)			8.6E+107 (.—.)	0.53 (0.03—8.15)	0.14 (0.02—1.04)
**Highest attained education**										
0—5 years primary education	11.09 (1.5—82.05)	0.89 (0.28—2.81)	0.34 (0.02—4.67)	0.76 (0.15—3.75)	2.09 (0.21—21.31)			8.1E-78 (.—.)	0.26 (0.0—16.1)	0.3 (0.02—5.47)
standard 6-8	(ref)	(ref)	(ref)	(ref)	(ref)			(ref)	(ref)	(ref)
secondary	57.88 (1.61—2080.34)	1.4 (0.3—6.59)	1.42 (0.27—7.35)	0.71 (0.24—2.11)	2.24 (0.63—7.94)			(empty)	0.34 (0.01—8.42)	0.49 (0.07—3.34)

---

* WQ= Wealth Quintile; the number at the end of the abbreviation indicates the stratum of interest

‡ No observations

§ No observations

For peer review only

For peer review only - http://bmjopen.bmj.com/site/about/guidelines.xhtml

post-secondary	42.93 (0.52—3528.5)	(empty)	(empty)	1.37 (0.08—24.48)	1.35 (0.27—6.7)				(empty)	0.37 (0.06—2.51)
**occupation**										
homemaker	0.8 (0.11—5.7)	0.61 (0.12—3.13)	21.78 (0.94—504.37)	0.79 (0.15—4.1)	5.97 (0.83—42.82)			4.7E+61 (.—.)	2.00 (0.04—100.23)	0.73 (0.0—155.92)
farming/fishing	(ref)	(ref)	(ref)	(ref)	(ref)			(ref)	(empty)	(ref)
employed	2.15 (0.29—16.04)	1.43 (0.23—9.04)	13.15 (0.9—192.03)	0.66 (0.17—2.58)	1.33 (0.23—7.83)			1.5E+154 (.—.)	(omitted)	0.19 (0.0—34.71)
**Indoor smoke**										
No	(empty)	0.26 (0.02—3.55)	(empty)	(empty)	(empty)				(empty)	0.75 (0.04—15.21)
Yes, inside the house	(empty)	2.91 (0.27—30.92)	(empty)	3.89 (0.65—23.19)	0.12 (0.01—1.5)				(empty)	1.64 (0.18—15.18)
a separate kitchen area	(ref)	(ref)	(ref)	(ref)	(ref)			(ref)	(ref)	(ref)
**History of Smoke**										
no	(ref)	(ref)	(ref)	(ref)	(ref)			(ref)	(ref)	(ref)
yes	0.29 (0.0—17.25)	3.07 (0.33—28.35)	0.12 (0.0—4.39)	0.94 (0.17—5.11)	0.26 (0.04—1.65)			4.1E+169 (.—.)	(empty)	0.54 (0.01—23.36)
**Diabetes**										
No	(ref)	(ref)	(ref)	(ref)	(ref)			(ref)	(ref)	(ref)
Yes	0.13 (0—16.29)	2.43 (0.29—20.13)	0.17 (0.0—9.5)	2.54 (0.54—12)	1.15 (0.19—7.16)			1.8E+46 (.—.)	0.07 (0.0—14.48)	1.15 (0.09—14.87)
Unknown	(empty)	(empty)	2.1E-08 (0.1—22.1)	7.2E+05 (0.0—.)	(empty)				(empty)	
**Dyslipidaemia**										
No	(ref)	(ref)	(ref)	(ref)	(ref)			(ref)	(ref)	(ref)
Yes	0.06 (0.0—4.64)	0.58 (0.07—4.84)	0.06 (0.0—1.92)	3.03 (0.53—17.14)	0.06 (0.0—1.19)			2.6E+07 (.—.)	0.31 (0.0—22.6)	0.93 (0.03—26.3)
missing data	(empty)	(empty)	7.7E+07 (0.0—.)	2.0E-06 (0.0—.)	0.18 (0.02—2.11)			(empty)	(empty)	0.48 (0—64.61)
**HIV status**										
Negative	(ref)	(ref)	(ref)	(ref)	(ref)			(ref)	(ref)	(ref)
Positive	0.08 (0.0—6.74)	1.15 (0.17—7.63)	0.12 (0.0—4.26)	1.94 (0.37—10.03)	1.41 (0.24—8.31)			1.45E-31 (.—.)	0.24 (0—22.56)	1.98 (0.14—27.98)
Unknown	2.1 (0.33—13.38)	1.66 (0.4—6.81)	1.33 (0.08—22.1)	2.04 (0.5—8.3)	1.63 (0.27—9.77)			1.45E-31 (.—.)	0.39 (0.02—7.7)	1.79 (0.23—13.66)

For peer review only - http://bmjopen.bmj.com/site/about/guidelines.xhtml

**Hypertension**										
No	(ref)	(ref)	(ref)	(ref)	(ref)			(ref)	(ref)	(ref)
Yes	0.11 (0.0—9.9)	0.7 (0.1—4.87)	0.03 (0.0—0.91)	0.85 (0.21—3.47)	0.6 (0.09—4.13)			1.81E+46 (.—.)	0.33 (0.01—19.43)	1.41 (0.14—14.27)
**CVDRF**										
2	0.03 (0.0—1.88)	0.8 (0.1—6.22)	0.0 (0.0—0.29)	1.59 (0.28—9.08)	1.29 (0.17—9.96)					
3	(ref)	(ref)	(ref)	(ref)	(ref)			(ref)	(ref)	(ref)
4	49.06 (0.47—5153.28)	1.87 (0.18—18.98)	2.7 (0.06—112.27)	0.92 (0.18—4.78)	16.97 (1.14—253.16)			1.81E+46 (.—.)	1.17 (0.0—154.19	0.58 (0.02—18.29)
5	(empty)	(empty)	(empty)	(empty)	(empty)			(omitted)	6.75 (0.0—24323.16)	0.48 (0.0—105.76)

For peer review only

Table 6: Stratification by highest attained education and obesity

Variables and variable categories	Non-obese & 0-5 years elementary education	Non-obese & 6-8 elementary education	Non obese & secondary education	Non-obese & post-secondary education[**]	Obese & 0-5 years elementary education[††]	Obese & 6-8 years elementary education	Obese & secondary education	Obese & post-secondary education[‡‡§§]
**Age**								
<50	(ref)	(ref)	(ref)	(ref)	(ref)	(ref)	(ref)	(ref)
50-60	10.76 (1.45—79.81)	1.33 (0.57—3.15)	2.18 (0.66—7.22)	.	2.07e+19 (.—.)	2.96 (0.6—14.49)	12.52 (0.3—525.67)	1.14E-57 (.—.)
>60	5.85 (0.9—38)	1.65 (0.63—4.29)	6.74 (1.83—24.92)	.	264.2 (.—.)	3.12 (0.43—22.52)	5.03 (0.02—1686.17)	1.64E+56 (.—.)
**Sex**								
Male	(ref)	(ref)	(ref)	(ref)	(omitted)	(ref)	(ref)	(ref)
Female	7.38 (0.9—60.6)	2.73 (0.77—9.71)	1.28 (0.35—4.69)	.		0.47 (0.03—8.69)	0.84 (0.02—45.4)	4.96E+56 (.—.)
**Site**								
Karonga	(ref)	(ref)	(ref)	(empty)	(ref)	(ref)	(ref)	(ref)
Lilongwe	5.38 (0.91—31.62)	3.06 (1.12—8.35)	0.88 (0.26—3.04)	(omitted)	3.6E+22 (.—.)	2.01 (0.23—17.74)	1.06 (0.01—97.43)	3.8E+107 (.—.)
**Wealth quintiles**								
poorest	25.05 (1.9—330.22)	1.59 (0.42—6.0)	1.72 (0.23—12.74)	.	1.8E+21 (.—.)	0.3 (0.03—3.47)	(empty)	(empty)
second	7.82 (0.71—86.4)	3.3 (1.02—10.68)	1.56 (0.28—8.67)		3.1E+42 (.—.)	0.37 (0.03—4.01)	13.09 (0.03—5949.07)	(empty)
third	(ref)	(ref)	(ref)	(empty)	(ref)	(ref)	(ref)	(empty)
fourth	2.4 (0.15—37.21)	1.85 (0.64—5.37)	1.96 (0.49—7.89)	(empty)	3.9E-21 (.—.)	0.22 (0.02—2.3)	0.24 (0—38.03)	(empty)
wealthiest	2.09 (0.1—42.23)	0.84 (0.24—2.92)	3.39 (0.75—15.3)	(omitted)	7.8 (.—.)	0.44 (0.04—4.71)	11.69 (0.09—1444.24)	(omitted)
**Marital status**								
single	(ref)	(ref)	(ref)	(ref)	(ref)	(ref)	(ref)	(ref)

[**] Site, wealth quintiles and occupation omitted because of collinearity
[††] Sex, occupation and history of smoking omitted because of collinearity
[‡‡] Wealth and occupation have no observations
[§§] History of smoking and CVDRF omitted because of collinearity

14

For peer review only

married	1.11 (0.28—4.43)	1.32 (0.54—3.23)	0.31 (0.11—0.91)	**0.0 (.—.)**	4.6E-22 (.—.)	0.69 (0.11—4.38)	0.13 (0.01—2.61)	7.38E+17
occupation								
homemaker	2.51 (0.56—11.17)	1.66 (0.54—5.08)	0.69 (0.17—2.84)	**0.0 (.—.)**	4.0 e+19 (.—.)	2.78 (0.29—26.69)	0.3 (0.01—12.69)	1.04E+95 (.—.)
farming/fishing	(ref)	(ref)	(ref)	(empty)	(empty)	(ref)	(empty)	(empty)
employed	1.87 (0.27—12.76)	1.83 (0.67—4.99)	0.5 (0.12—2.03)	**1 (omitted)**	1 **(omitted)**	1.71 (0.15—19.93)	1 (omitted)	(omitted)
Indoor smoke								
No	0.96 (0.04—21.49)	(Empty)	(Empty)	(empty)	(empty)	(empty)	(empty)	4.62E+74 (.—.)
Yes, inside the house	1.93 (0.21—17.91)	1.88 (0.42—8.4)	0.11 (0.01—1.59)	(empty)		0.93 (0.05—18.44)	5.99 (0.04—988.69)	4.72E+94 (.—.)
Yes, in a separate kitchen area	(ref)	(ref)	(ref)	(ref)	(ref)	(ref)	(ref)	(ref)
Smoking history								
no	(ref)	(ref)	(ref)	(ref)	(ref)	(ref)	(ref)	(ref)
yes	10.25 (0.96—109.45)	1.29 (0.29—5.66)	0.25 (0.05—1.3)	**0.0 (.—.)**	(empty)	0.35 (0.01—14.51)	9.43E+07 (0.0—.)	(empty)
Diabetes								
No	(ref)	(ref)	(ref)	(ref)	(ref)	(ref)	(ref)	(ref)
Yes	1.06 (0.1—11.91)	0.88 (0.21—3.71)	1.28 (0.26—6.33)	**0.0 (.—.)**	1.2E-20 (.—.)	0.53 (0.05—5.5)	1.4e+07 (0.0—.)	2.0E+163 (.—.)
Unknown	(empty)	0.22 (0.01—5.99)	(empty)	(empty)		(empty)	(empty)	
Dyslipidaemia								
No	(ref)	(ref)	(ref)	(ref)	(ref)	(ref)	(ref)	(ref)
Yes	1.05 (0.12—9.23)	0.43 (0.11—1.75)	2.43 (0.38—15.56)	**0.0 (.—.)**	1.4E+23 (.—.)	1 (0.05—21.22)	32.63 (0.17—6363.24)	1.5E-127 (.—.)
missing data	0.41 (0.02—7.53)	3.39 (0.18—65.18)	0.14 (0.01—2.05)	(empty)		(empty)	109.64 (0.06—205197.3)	(empty)
HIV status								
Negative	(ref)	(ref)	(ref)	(ref)	(ref)	(ref)	(ref)	(ref)
Positive	0.85 (0.08—9.02)	0.6 (0.14—2.55)	2.5 (0.62—10.07)	.	5.5E+23 (.—.)	0.88 (0.05—17.25)	24.16 (0.59—992.53)	1.6E+105 (.—.)

For peer review only

For peer review only - http://bmjopen.bmj.com/site/about/guidelines.xhtml

Unknown	0.87 (0.15—5.16)	1.34 (0.52—3.45)	2.37 (0.6—9.4)	.	(empty)	0.75 (0.14—3.89)	157.01 (0.5—49479.48)	8.25E-77 (.—.)
**Hypertension**								
No	(ref)	(ref)	(ref)	(ref)	(ref)	(ref)	(ref)	(ref)
Yes	1.58 (0.23—10.99)	0.51 (0.13—1.97)	0.68 (0.13—3.57)	0.0 (.—.)	4.4E-38 (.—.)	0.44 (0.04—5.13)	2.37 (0.06—95.27)	6.1E+132 (.—.)
**CVDRF**								
2	0.53 (0.05—5.26)	0.18 (0.04—0.8)	2.39 (0.45—12.74)	.				(ref)
3	(ref)	(ref)	(ref)	(ref)	(ref)	(ref)	(ref)	2.43E+94 (.—.)
4	2.88 (0.28—29.86)	2.42 (0.57—10.31)	0.62 (0.1—3.8)	.	1.4E-22 (.—.)	0.99 (0.04—23.46)	27.92 (0.04—17774.42)	(omitted)
≥5		(empty)	21.02 (0.37—1193.6)	(empty)	4.4e-07 (.—.)	1.95 (0.01—423.84)	1.44 (0.0—2718.99)	1.14E-57 (.—.)

16

STROBE Statement—Checklist of items that should be included in reports of ***cross-sectional studies***

	Item No	Recommendation	Page No
**Title and abstract**	1	(*a*) Indicate the study's design with a commonly used term in the title or the abstract	1
		(*b*) Provide in the abstract an informative and balanced summary of what was done and what was found	2
**Introduction**			
Background/rationale	2	Explain the scientific background and rationale for the investigation being reported	4
Objectives	3	State specific objectives, including any prespecified hypotheses	5
**Methods**			
Study design	4	Present key elements of study design early in the paper	5
Setting	5	Describe the setting, locations, and relevant dates, including periods of recruitment, exposure, follow-up, and data collection	5
Participants	6	(*a*) Give the eligibility criteria, and the sources and methods of selection of participants	5
Variables	7	Clearly define all outcomes, exposures, predictors, potential confounders, and effect modifiers. Give diagnostic criteria, if applicable	6-7
Data sources/ measurement	8*	For each variable of interest, give sources of data and details of methods of assessment (measurement). Describe comparability of assessment methods if there is more than one group	6
Bias	9	Describe any efforts to address potential sources of bias	11
Study size	10	Explain how the study size was arrived at	7
Quantitative variables	11	Explain how quantitative variables were handled in the analyses. If applicable, describe which groupings were chosen and why	7
Statistical methods	12	(*a*) Describe all statistical methods, including those used to control for confounding	7
		(*b*) Describe any methods used to examine subgroups and interactions	7
		(*c*) Explain how missing data were addressed	
		(*d*) If applicable, describe analytical methods taking account of sampling strategy	7
		(*e*) Describe any sensitivity analyses	
**Results**			
Participants	13*	(a) Report numbers of individuals at each stage of study—eg numbers potentially eligible, examined for eligibility, confirmed eligible, included in the study, completing follow-up, and analysed	8
		(b) Give reasons for non-participation at each stage	
		(c) Consider use of a flow diagram	16
Descriptive data	14*	(a) Give characteristics of study participants (eg demographic, clinical, social) and information on exposures and potential confounders	8
		(b) Indicate number of participants with missing data for each variable of interest	8
Outcome data	15*	Report numbers of outcome events or summary measures	8
Main results	16	(*a*) Give unadjusted estimates and, if applicable, confounder-adjusted estimates and their precision (eg, 95% confidence interval). Make clear which confounders were adjusted for and why they were included	9

For peer review only

		(*b*) Report category boundaries when continuous variables were categorized	
		(*c*) If relevant, consider translating estimates of relative risk into absolute risk for a meaningful time period	
Other analyses	17	Report other analyses done—eg analyses of subgroups and interactions, and sensitivity analyses	9
**Discussion**			
Key results	18	Summarise key results with reference to study objectives	9
Limitations	19	Discuss limitations of the study, taking into account sources of potential bias or imprecision. Discuss both direction and magnitude of any potential bias	2 & 12
Interpretation	20	Give a cautious overall interpretation of results considering objectives, limitations, multiplicity of analyses, results from similar studies, and other relevant evidence	10-12
Generalisability	21	Discuss the generalisability (external validity) of the study results	10-12
**Other information**			
Funding	22	Give the source of funding and the role of the funders for the present study and, if applicable, for the original study on which the present article is based	8

*Give information separately for exposed and unexposed groups.

Note: An Explanation and Elaboration article discusses each checklist item and gives methodological background and published examples of transparent reporting. The STROBE checklist is best used in conjunction with this article (freely available on the Web sites of PLoS Medicine at http://www.plosmedicine.org/, Annals of Internal Medicine at http://www.annals.org/, and Epidemiology at http://www.epidem.com/). Information on the STROBE Initiative is available at www.strobe-statement.org.

For peer review only

For peer review only - http://bmjopen.bmj.com/site/about/guidelines.xhtml
